# Genome of the ramshorn snail *Biomphalaria straminea*—an obligate intermediate host of schistosomiasis

**DOI:** 10.1093/gigascience/giac012

**Published:** 2022-02-15

**Authors:** Wenyan Nong, Yifei Yu, Madeleine E Aase-Remedios, Yichun Xie, Wai Lok So, Yiqian Li, Cheuk Fung Wong, Toby Baril, Sean T S Law, Sheung Yee Lai, Jasmine Haimovitz, Thomas Swale, Shan-shan Chen, Zhen-peng Kai, Xi Sun, Zhongdao Wu, Alexander Hayward, David E K Ferrier, Jerome H L Hui

**Affiliations:** School of Life Science, Simon F.S. Li Marine Science Laboratory, State Key Laboratory of Agrobiotechnology, The Chinese University of Hong Kong, Hong Kong, China; School of Life Science, Simon F.S. Li Marine Science Laboratory, State Key Laboratory of Agrobiotechnology, The Chinese University of Hong Kong, Hong Kong, China; The Scottish Oceans Institute, Gatty Marine Laboratory, School of Biology, University of St. Andrews, St. Andrews, UK; School of Life Science, Simon F.S. Li Marine Science Laboratory, State Key Laboratory of Agrobiotechnology, The Chinese University of Hong Kong, Hong Kong, China; School of Life Science, Simon F.S. Li Marine Science Laboratory, State Key Laboratory of Agrobiotechnology, The Chinese University of Hong Kong, Hong Kong, China; School of Life Science, Simon F.S. Li Marine Science Laboratory, State Key Laboratory of Agrobiotechnology, The Chinese University of Hong Kong, Hong Kong, China; School of Life Science, Simon F.S. Li Marine Science Laboratory, State Key Laboratory of Agrobiotechnology, The Chinese University of Hong Kong, Hong Kong, China; University of Exeter, Exeter, UK; School of Life Science, Simon F.S. Li Marine Science Laboratory, State Key Laboratory of Agrobiotechnology, The Chinese University of Hong Kong, Hong Kong, China; School of Life Science, Simon F.S. Li Marine Science Laboratory, State Key Laboratory of Agrobiotechnology, The Chinese University of Hong Kong, Hong Kong, China; Dovetail Genomics, California, USA; Dovetail Genomics, California, USA; Institute of Agro-food Standard and Testing Technology, Shanghai Academy of Agricultural Sciences, Shanghai, China; School of Chemical and Environmental Engineering, Shanghai Institute of Technology, Shanghai, China; Sun Yat-sen University, Guangdong, China; Sun Yat-sen University, Guangdong, China; University of Exeter, Exeter, UK; The Scottish Oceans Institute, Gatty Marine Laboratory, School of Biology, University of St. Andrews, St. Andrews, UK; School of Life Science, Simon F.S. Li Marine Science Laboratory, State Key Laboratory of Agrobiotechnology, The Chinese University of Hong Kong, Hong Kong, China

## Abstract

**Background:**

Schistosomiasis, or bilharzia, is a parasitic disease caused by trematode flatworms of the genus *Schistosoma*. Infection by *Schistosoma mansoni* in humans results when cercariae emerge into water from freshwater snails in the genus *Biomphalaria* and seek out and penetrate human skin. The snail *Biomphalaria straminea* is native to South America and is now also present in Central America and China, and represents a potential vector host for spreading schistosomiasis. To date, genomic information for the genus is restricted to the neotropical species *Biomphalaria glabrata*. This limits understanding of the biology and management of other schistosomiasis vectors, such as *B. straminea*.

**Findings:**

Using a combination of Illumina short‐read, 10X Genomics linked‐read, and Hi‐C sequencing data, our 1.005 Gb *B. straminea* genome assembly is of high contiguity, with a scaffold N50 of 25.3 Mb. Transcriptomes from adults were also obtained. Developmental homeobox genes, hormonal genes, and stress-response genes were identified, and repeat content was annotated (40.68% of genomic content). Comparisons with other mollusc genomes (including Gastropoda, Bivalvia, and Cephalopoda) revealed syntenic conservation, patterns of homeobox gene linkage indicative of evolutionary changes to gene clusters, expansion of heat shock protein genes, and the presence of sesquiterpenoid and cholesterol metabolic pathway genes in Gastropoda. In addition, hormone treatment together with RT-qPCR assay reveal a sesquiterpenoid hormone responsive system in *B. straminea*, illustrating that this renowned insect hormonal system is also present in the lophotrochozoan lineage.

**Conclusion:**

This study provides the first genome assembly for the snail *B. straminea* and offers an unprecedented opportunity to address a variety of phenomena related to snail vectors of schistosomiasis, as well as evolutionary and genomics questions related to molluscs more widely.

## Background

With >240 million people worldwide estimated to require treatment, the World Health Organization considers schistosomiasis to be the second most prevalent parasitic disease after malaria [1]. As such, schistosomiasis is a global health problem that causes considerable economic and social burdens.

Infection by *Schistosoma mansoni* (NCBI:txid6183) in humans results when cercariae emerge into the water from their freshwater snail intermediate hosts in the genus *Biomphalaria*, and seek out and penetrate submerged body parts through the skin. Once inside the human body, adult worms lay eggs, which are deposited in the blood venules and will cross the intestinal wall to leave the body in the faeces. In addition, eggs that fail to cross the intestinal wall (named “reflux eggs”) circulate to the liver where they grow, emerge, and cause disease. Miracidia larvae hatch from eggs that reach water, then seek out and penetrate a new snail intermediate host. Following this, sporocysts develop in the infected snails, and subsequently free-living cercariae emerge from the snail into the water, completing the parasitic life cycle. Among the 34 described species of *Biomphalaria* snails, 18 species (including *Biomphalaria straminea*) have been demonstrated to be potential vectors for *S. mansoni*. Different geographical locations are dominated by different species of *Biomphalaria*.

**Figure 1:**
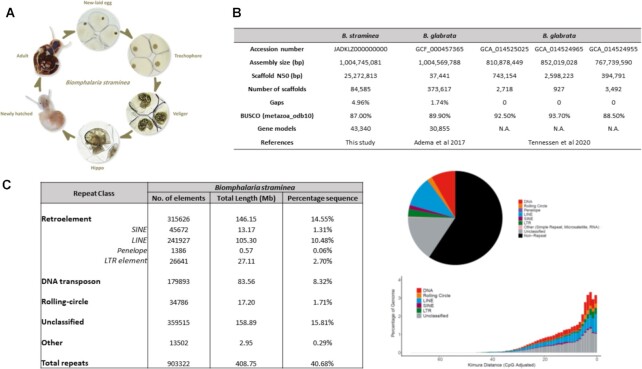
: (A) Life cycle of snail *Biomphalaria straminea*; (B) comparison of snail *Biomphalaria* genome assembly quality [12, 13]; (C) transposable elements in *Biomphalaria straminea*.

The native range of *Biomphalaria* snails is South America and Africa [2, 3]. However, several species have been introduced to other areas, presenting a risk of schistosomiasis infection. The occurrence of *B. straminea* in Asia was first reported at Lam Tsuen valley in Hong Kong during the 1970s ([4]; Fig. 1A), presumably having somehow spread from its native range in South America into Central America and southern China [5]. *B. straminea* have since been identified at a number of locations in Hong Kong and Guangdong Province [4, 6–9]. While *S. mansoni* is not yet endemic in either Hong Kong or mainland China, cases of schistosomiasis caused by the parasite are currently increasing in China. According to the records from the database of the National Notifiable Disease Report System, 355 cases of imported schistosomiasis cases had been reported in 15 provinces in China between 1979 and 2019, including 78 cases infected with *S. mansoni*, 262 cases with *Schistosoma haematobia*, and 15 cases with unidentified *Schistosoma*. Since *B. straminea* had already been discovered in Guangdong province in southern China, it is believed that the imported *S. mansoni* increases the risk of its transmission in China [10, 11].

Whole-genome sequences are valuable resources for obtaining deeper understanding of the biological characteristics of any organism. Despite the importance of the phylum Mollusca, there is a lack of genomic resources [88]. In the case of *B. straminea*, such a resource will affect questions of how they may interact with *S. mansoni* and how similar the genetic mechanisms are between different *Biomphalaria* species, with possible implications for how treatments and management strategies might be transferable. To date, only the genome of *Biomphalaria glabrata* has been sequenced and analysed ([12, 13]; Fig. 1B), and a high-quality genome of *B. straminea* is lacking, hindering further understanding of the species. To address this issue, we provide and analyse a high-quality genome assembly for *B. straminea* together with accompanying transcriptomes.

## Results and Discussion

### Genome quality evaluation

Genomic DNA (gDNA) was extracted from single individuals of *B. straminea* (Fig. 1A). Genome sequences were first assembled using short reads followed by scaffolding with Hi-C data. The genome assembly (without the mitochondrial genome) is 1.005 Gb with a scaffold N50 of 25.3 Mb (Fig. 1B). This high physical contiguity is matched by high completeness, with an 87.0% complete BUSCO score [14] (Fig. 1B). A total of 43,340 gene models, including 3,122 transfer RNA and 40,218 protein-coding genes, were generated by mapping transcriptome data to the genome assembly (Supplementary Information S1). The mean exon length is 262 bp, mean intron length is 1,603 bp, and mean deduced protein length is 377 amino acids. The genome quality generated in this study is comparable to the previously published genome assemblies of another schistosomiasis-carrying vector snail, *B. glabrata* ([12, 13]; Fig. 1B).

### Repeat element analysis

We identified a total repeat content of 40.68% in the genome of *B. straminea* (Fig. 1C), demonstrating that repeats make up a large proportion of total genome size in the species. A considerable proportion of repeats were unclassified (15.81%), suggesting that many of the annotated repeats represent new repeat families (Fig. 1C), which is not unexpected given the relatively sparse attention given to the analysis of repeats in gastropod molluscs to date. Of the remaining repeats, LINE elements and DNA transposons are most abundant (LINEs: 10.48%, DNA transposons: 8.32%), whereas SINEs, LTR elements, and rolling-circle elements are present only in low proportions (LTR elements: 2.70%, rolling-circle elements: 1.71%, SINEs: 1.31%) (Fig. 1C). Consideration of a repeat landscape plot suggests that there has been a long-term ongoing expansion of repeats in *B. straminea*, with a recent spike in activity. The recent spike is evident from the relatively large percentage of repeats in the genome that are separated from their family consensus sequences by short distances, while the long tail of increasing divergence from the consensus is suggestive of a gradual increase in activity over a relatively long period (Fig. 1C). LINEs and DNA transposons have expanded most significantly; however, there has also been a less considerable expansion of LTR and rolling circle elements (Fig. 1C).

### Homeobox-containing gene content and linkage

#### Hox cluster genes

Homeobox genes are transcription factors involved in regulating animal development. Not only are they highly conserved between distantly related lineages, but also many of the genes are linked or clustered in genomes. Besides the most well-known clusters such as the Hox and ParaHox clusters, many homeobox genes are linked including other ANTP class genes in NK and SuperHox clusters, and also amongst other classes of PRD, TALE, and SINE homeobox genes [15–17]. These clusters have been maintained or dispersed differently in different animal lineages. Changes to gene clustering may represent the breakdown of regulatory constraints that normally maintain clusters and are thought to be the mechanism holding together the tightly regulated Hox cluster, for instance. Genomic clustering also reflects the ancient origins of many of these homeobox genes by tandem duplication, e.g., the 4 ANTP clusters in the bilaterian ancestor that arose via subsequent expansions from a single proto-ANTP gene [18]. Among molluscs, a diverse phylum to which gastropods belong, alongside other conchiferans (monoplacophorans, bivalves, scaphopods, and cephalopods), as well as aculiferans (aplacophorans and polyplacophorans), some of the diversity of body plans may be underpinned by changes to developmental genes like homeobox genes. Hox genes have been co-opted to the development of novel morphological structures in cephalopods [89], and this corresponds to a breakdown of the Hox cluster across several large scaffolds, and the loss of a few genes [90]. Other mollusc genomes show a breakdown of homeobox clustering overall, like the Pacific oyster (*Crassostrea gigas* [19]), while a more recent chromosome-level assembly reveals large-scale patterns of linkage in *Magallana hongkongensis* [20]. This genome assembly of *B. straminea* improves our understanding of homeobox gene linkage in comparison to other molluscs, which are lophotrochozoans and, alongside well-studied ecdysozoans like flies, provide a more thorough protostome comparison to vertebrates, which are within the Deuterostomia.

**Figure 2:**
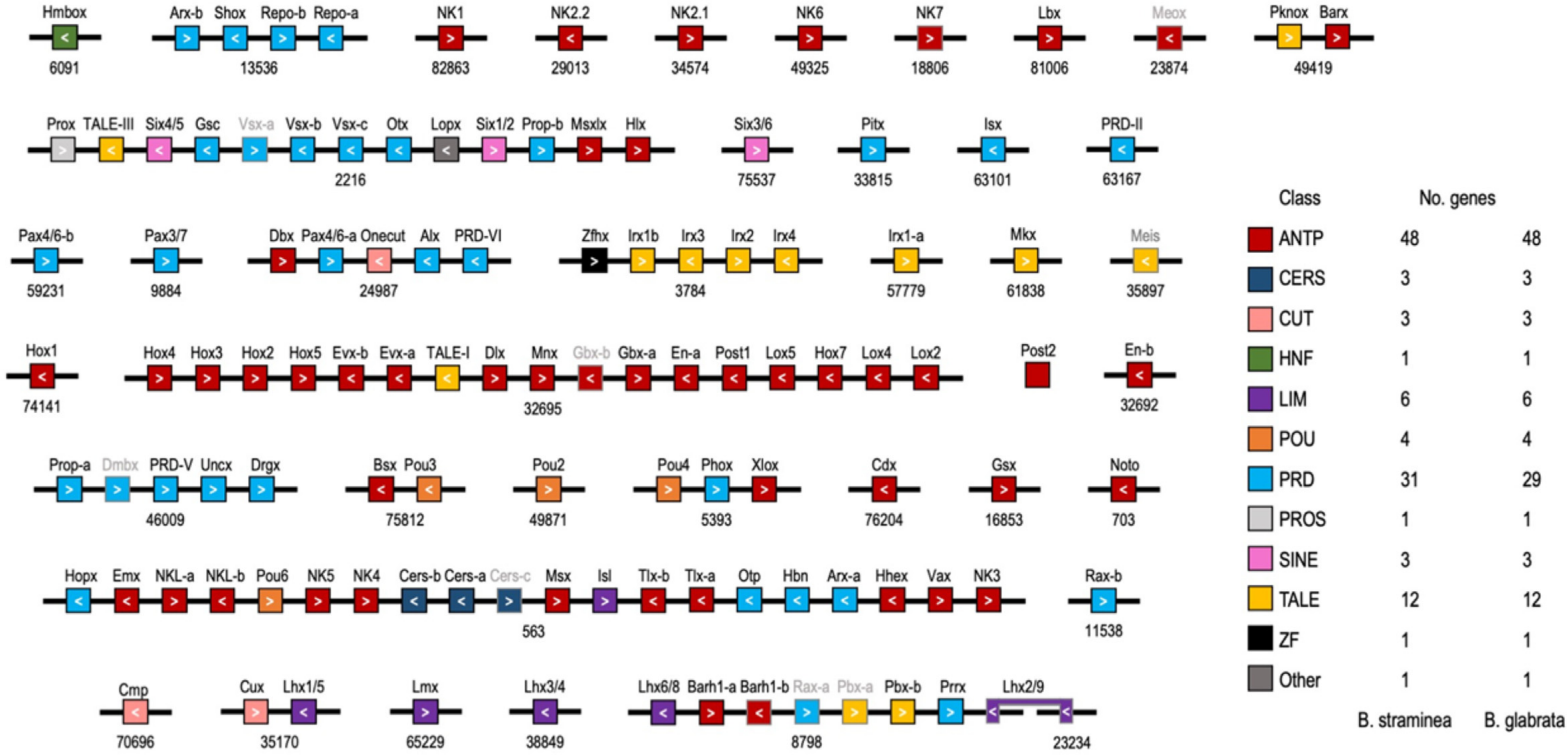
: Distribution of Homeoboxes in the genome of *Biomphalaria straminea*. Class is denoted by colour; arrows show orientation on each scaffold, which are represented by black lines and are numbered underneath. *Post2* is not found in the genomic sequence but is found in the transcriptome so is not shown on a scaffold. Grey gene names and box outlines denote partial homeodomain sequences.

We found 114 homeobox genes in the genome of *B. straminea*, belonging to 11 recognized classes, and 1 lophotrochozoan-specific gene, *Lopx* (Supplementary information S2a; [21]). Many of these genes are clustered (situated on the same chromosome with no or very few non-homeobox genes in between) or linked (on the same chromosome, but with intervening non-homeobox genes) in the genome (Fig. 2). Nine of the 11 Hox genes are found on scaffold 32,695, in an arrangement that suggests several intrachromosomal rearrangements. In an ordered cluster as seen in the gastropod *L. gigantea*, for instance, the Hox genes are situated in the genome in the ancestral bilaterian order from anterior-acting *Hox1* to posterior-acting *Post1*, and no other non-Hox genes are found amongst the Hox genes [22]. Here, however, we find that *Hox2, Hox3*, and *Hox4* are upstream of *Hox5*. In addition, *Hox2–Hox5* are downstream of the posterior half of the cluster, including *Lox5, Hox7, Lox4, Lox2*, and *Post1. Hox1* is found on another scaffold, while the sequence for *Post2* is not in the genomic assembly, although its sequence is found in our transcriptome data. The Hox arrangement in *B. straminea* provides more linkage information than the *B. glabrata* assembly, where the short scaffolds corroborate only fragments of the Hox cluster like the linkage of *Hox4, Hox3*, and *Hox2* but do not confirm the rearrangements in *B. straminea*, such as the linkage of *Hox5* to *Hox2* (Supplementary Information S2b). We do see a difference in the arrangement of the posterior half of the Hox cluster, however, where in *B. glabrata, Lox4, Lox2, Post2*, and *Post1* are linked in that order on scaffold 139, with *Lox4* and *Lox2* in the negative strand and *Post2* and *Post1* on the positive, which is slightly different from many other molluscs in which only *Post1* differs in orientation relative to the remainder of the posterior end of the Hox cluster genes [20, 22]. In *B. straminea*, there has been a rearrangement separating *Post1*, placing it with *Lox5* and *Hox7* and in the same orientation as *Lox4* and *Lox2* (Fig. 2). Thus, the Hox genes of *Biomphalaria* seem highly rearranged relative to the ancestral order and each other. Clearly then, there are no (or minimal) long-range regulatory mechanisms operating across these genes that could have constrained their organization and prevented rearrangement. At most, there may be remains of some form of subcluster mechanisms, such as enhancer sharing, operating over the small regions (i.e., *Hox2–4* and *Lox2–4*) whose similar arrangement may be indicative of constraints conserved across *Biomphalaria* species. Future expression and regulatory element analyses may help resolve this possibility.

#### ParaHox cluster genes

The ParaHox cluster is the evolutionary sister to the Hox cluster [91]. The homeodomains of the 3 ParaHox genes (*Gsx, Xlox*, and *Cdx*) are found on 3 separate scaffolds in *B. straminea* (Fig. 2); however, 3 upstream exons of *Cdx* are on scaffold 5,393, which also has the *Xlox* gene (Supplementary Information S2a). This is in contrast to the genome of *B. glabrata*, where *Gsx* and *Xlox* are linked on scaffold 3 (Supplementary Information S2a and b). Perhaps this pattern reflects maintained linkage between all 3 ParaHox genes in *Biomphalaria* species and only because of the draft level of all the assemblies this is not evident. However, if this is the case, the ParaHox genes are separated by large amounts of sequence and have not retained the ancestral order of *Gsx-Xlox-Cdx. B. glabrata Xlox* is nearly 4 Mb from the start of its scaffold, while in *B. straminea, Xlox* is at a location with another homeobox-containing gene (*Phox*) 15 Mb away on 1 side and the first 3 *Cdx* exons are almost 5 Mb away on the other side of *Xlox*. Thus, although the *Biomphalaria* ParaHox genes may be linked, they cannot be considered to be clustered. This dispersal of ParaHox genes is typical for molluscs in general, with several species also showing loose linkage of some of the genes [20], which contrasts with the relatively tight clustering of these genes in many deuterostomes [23–25] and the likely pan-cluster regulation that may operate in these deuterostomes.

#### ANTP-class homeobox genes

Beyond Hox and ParaHox, there are other linkages among and between the classes of homeobox genes that hint at their ancient evolutionary origins and genomic arrangement in clusters. Despite the many rearrangements to the Hox cluster, many genes linked to Hox clusters in other species are also found on the same scaffold in *B. straminea*, including *Mnx, Gbx-a* and *Gbx-b, En-a, Evx-a* and *Evx-b*, and *Dlx* [15, 18, 20, 26, 27]. These linkages give further support for the hypothesized Super-Hox cluster of non-Hox ANTP-class genes linked to the Hox genes in bilaterians [15].

#### SINE homeobox genes

Another highly conserved cluster besides Hox and ParaHox is the SINE-class cluster, typically composed of the *Six3/6, 1/2*, and *4/5* genes or their protostome orthologues [16]. In *B. straminea, Six4/5* and *Six1/2* are on the same scaffold but with a number of genes between them, and *Six3/6* is on a distinct scaffold (Fig. 2). In *B. glabrata, Six3/6* is linked to *Hlx* (Supplementary Information S2b), the last homeobox gene at the end of the *Six4/5-Six1/2* scaffold in *B. straminea* (Fig. 2). Thus, there is clearly not a SINE-class gene cluster conserved in *B. straminea*, but the linkage of at least some of these genes indicates that the dispersal of this cluster has not yet proceeded to the extent of these genes being separated onto different chromosomes. Also, the location of the *Hlx* gene relative to different *Six* genes indicates a certain degree of genomic rearrangement between the 2 *Biomphalaria* species (i.e., conserved macrosynteny but divergent microsynteny).

#### IRX homeobox genes

Homeobox genes in the IRX family within the TALE class are also observed to be clustered in several lineages, e.g., the 3-gene (*ara, caup*, and *mirr*) cluster in *Drosophila*, 2 clusters of 3 genes in vertebrates, and 4 genes in the limpet *L. gigantea* (*irx4, irx2, irx1*, and *irx3*) [28–30]. These clusters are thought likely to have arisen convergently by independent tandem duplications in the arthropod, vertebrate, and mollusc lineages [31, 28–30]. Both *Biomphalaria* species have 5 IRX-family genes, 1 pair of which seems to be a product of a more recent, possibly *Biomphalaria*-specific, duplication (*Irx1-a* and *Irx1-b*). Perhaps surprisingly, none of the *Biomphalaria Irx* genes, *Irx1* (*a* and *b*), *Irx2, Irx3*, and *Irx4*, show clear orthology to specific gastropod (limpet) or bivalve (oyster) genes in a phylogenetic tree (Supplementary Information S2c). A paucity of phylogenetically informative amino acid changes is the most likely explanation for this lack of resolution. Despite this lack of resolution of *Irx* orthology across species the *B. straminea* genome assembly does provide a new example of *Irx* gene clustering. *Irx3, Irx2*, and *Irx4* are closely clustered in the genome, while *Irx1-b* is 7 Mb away on the same scaffold, also with *Zhx*, a ZF-class gene another 6 Mb further. The 2 *Irx1* paralogues, however, are on separate scaffolds, which may represent either a rearrangement following their duplication, convergence of the sequence of the homeodomain, or thirdly, an assembly artefact. In *B. glabrata*, only the linkage of *Irx4* with *Irx2* is corroborated owing to the shorter scaffold lengths of that assembly. Further work, perhaps using other conserved domains from these genes and with a wider breadth of lophotrochozoan species, could potentially determine whether in fact the 4 *Irx* gene types in *Biomphalaria* species are orthologous to genes in other species’ *Irx* clusters. A multi-gene IRX-family cluster in *Biomphalaria* species with evidence of ≥1 independent expansion (*Irx1-a* and *Irx1-b*) provides an interesting addition to our understanding of IRX-family clusters, and the mechanisms behind gene expansions and subsequent maintenance of clustering in general.

#### PRD- and LIM-class homeobox genes

We also observe linkages amongst PRD-class genes, with clusters on scaffolds 13,536, 2,216, 46,009, and 563 (Fig. 2). The PRD-class cluster that is widely found across various species is the so-called HRO cluster, composed of the genes *Otp, Rx/Rax*, and *Hbn/Arx-like* [16, 17], which ancestrally was likely embedded within a more extensive PRD/LIM-class mega-cluster, including the PRD-class genes *Gsc* and *Otx* and the LIM-class gene *Isl* [16]. In *B. straminea* there is a remnant of the HRO cluster, with *Otp* clustered with *Hbn*, internally on a large scaffold (563) and flanked by other homeobox genes (Fig. 2) including another PRD-class gene (*Arx-a*) now in this *Biomphalaria* PRD-class cluster, but the *Rax* genes are on other scaffolds. Interestingly, the *Isl* gene is also on this large 563 scaffold in *B. straminea*, consistent with descent from the hypothesized PRD/LIM-class mega-cluster [16]. *B. glabrata* provides an interesting contrast as the HRO cluster is now complete (with *Otp, Hbn*,and*Rax-b*), in contrast to *B. straminea*, and again *Arx-a* is also in the *Biomphalaria* cluster (Fig. 2; Supplementary Fig. S2b). Why the PRD-class HRO cluster would remain intact in 1 species of *Biomphalaria* but not the other remains to be resolved. Also, whether the inclusion of the *Arx-a* gene in this cluster in these snails is found elsewhere in the animal kingdom and is of any functional significance also remains a topic for future work. Overall, the PRD-class gene clustering provides a mixed signal, of both conservation of remnants of ancient clustering alongside rearrangements between closely related, con-generic species.

#### Duplicated homeobox genes

There are several duplications shared between the 2 species, which we infer to be at least ancestral to the genus. These include paralogues of *Arx, Pax4/6, Irx1, En, Evx, Abox, Barhl, Pbx*, and *Tlx*, as well as 3 paralogues of *Vsx* and *Cers*. Notably, the 2 paralogues each of *Vsx* and *Cers* genes remain clustered in the genome, reflecting their likely origin by tandem duplication. This is also seen for *En, Tlx, Evx*, and *Abox. B. straminea* is the only species of the 2 with 2 paralogues of *Gbx*, although 1 has an apparently odd arrangement that would mean it is unlikely to be a functional gene, if this arrangement were real. The homeodomain is split across 2 exons, the first of which is in 1 orientation, while there are 2 copies of the second exon in the opposite orientation, indicating that the second *Gbx* gene may be a pseudogene or an assembly artefact (Supplementary Information S2a).

#### Giga-cluster homeobox genes

An overarching framework for understanding the genomic organization of homeobox-containing genes comes from hypotheses about their ancient linkage patterns following their presumed origins largely via tandem duplications. This ancestral clustering goes beyond the class-specific clusters already described above and is captured by the Giga-cluster hypothesis [16]. High-quality genome assemblies, such as that described here for *B. straminea*, are key resources for testing this hypothesis and potentially expanding it. Several instances of linkage of different classes of homeobox gene are present in the *B. straminea* assembly, most notably on scaffolds 563, 8,789, 2,216, and 24,987 (Fig. 2). Scaffold 2,216 is interesting for the linkage of the SINE-class genes *Six4/5* and *Six1/2* with some of the members of the ancestral PRD/LIM-class Mega-cluster (i.e., the PRD-class genes *Gsc* and *Otx*) that has undergone some dispersal in the *Biomphalaria* lineage (as described above). Also, some of the other members of this dispersed PRD/LIM Mega-cluster (*Isl, Otp, Hbn*) are on scaffold 563, which are now linked with many members of the dispersed NK-cluster (e.g., *NK5, NK4, Msx, Tlx-a* and *-b*, and *NK3*) as well as a member of the ancestral SuperHox cluster (i.e., *Hhex*) [15, 16]. Other members of the SuperHox cluster are still linked with the true Hox genes (EuHox genes) on scaffold 32,695. These linkages of genes from different homeobox classes along with the further new instances of interclass linkage on scaffold 8,798 (Fig. 2) are all consistent with the Giga-cluster hypothesis [16]. However, how much of all of these linkages represent ancestral associations (i.e., descended from primary clustering) versus instances of coming together in the genome convergently in evolution (i.e., secondary clustering) should be resolvable with comparisons to further high-quality genome sequences as well as a better understanding of the dynamics of genome evolution and rearrangements (reviewed in [16]).

### Synteny analysis of *B. straminea* with other molluscs

The homeobox analyses described above provide instances of linkages that indicate varied synteny conservation across various mollusc and animal clades, even between the 2 *Biomphalaria* species now sequenced. The *B. straminea* genome shows considerable conserved linkage within and between classes of homeobox, and the maintenance of certain conserved clusters or linkages observed throughout wider lineages (i.e., instances of remnants of the Hox, ParaHox, SuperHox, and Giga-clusters [16]). In comparison to *B. glabrata*, in which less linkage can be observed because of shorter scaffold lengths, there is some conserved synteny. A few differences between the species may be due to species-specific genomic rearrangements resulting in the disruption of gene order, but the alternative possibility of assembly artefacts cannot be excluded entirely at present without further work. Of particular interest for further study is the major rearrangement of the Hox cluster in *B. straminea*. Perhaps more thorough sequencing of *B. glabrata* or assemblies of additional *Biomphalaria* species could determine whether this is shared in the genus or is a novelty of *B. straminea*. Regardless of this, the impact of this rearrangement on Hox gene expression and function is of interest. Hox cluster rearrangements could indicate the loss of shared regulatory elements that constrain Hox clusters in other lineages and may reflect changes to Hox gene expression, perhaps underpinning developmental changes in these snails. Similarly, the impacts of the dispersal of the ParaHox cluster on gene expression will be interesting to resolve. The patterns of clustering, linkage, and rearrangement of homeobox genes are good markers for genome organization, and these results show that key differences between the species may represent higher levels of genomic divergence than expected for these 2 snails. Here we observe specific cases of differences between our new *B. straminea* genome and that of *B. glabrata* within the context of ancestral linkages, and this pattern may be a good indicator of wider differences between the genetics and molecular processes operating in the 2 species.

To examine the syntenic relationships more generally between *Biomphalaria* and mollusc genomes, we constructed Oxford dot plots, comparing the chromosomal positions of orthologous genes between published mollusc genomes, as available from GenBank for gastropod, bivalve, and cephalopod molluscs. As shown in Fig. 4, the relationship of pseudo-chromosomes (2n = 36 [12]) and scaffolds between *B. straminea* and molluscs of other classes were conserved in most cases. Previous phylogenetic tree constructions for different *Biomphalaria* species suggested a monophyletic clade of African species with the remaining lineages being neotropical species [2, 3]. Based on this phylogenetic relationship, our data show that the neotropical species have not undergone any significant interchromosomal rearrangements from their last common ancestor after separation to different geographical regions. One-to-one synteny block could be identified between *B. straminea* and the eupulmonata gastropod, *Achatina immaculata*. However, in the comparison of *B. straminea* to the more evolutionarily distant species, a few one-to-many blocks were found. These patterns indicated that some chromosome duplication and alteration occurred from the most recent common ancestor of *B. straminea, B. glabrata*, and *A. immaculata* (the ancestor of Hygrophila and Eupulmonata). Furthermore, species with closer evolutionary distance shared more similar synteny patterns against *B. straminea* (e.g., between *Pomacea canaliculata* and *Marisa cornuarietis*, as well as between *C. gigas* and *M. hongkongensis*, which share more similar synteny blocks), suggesting the dynamic changes of chromosome arrangements in different molluscs. In *Octopus sinensis*, the gene order and synteny blocks to *B. straminea* were largely lost, suggesting that more duplication, translocation, and rearrangement events occurred since the divergence of *O. sinensis* (Cephalopoda) and the common ancestor of Gastropoda and Bivalvia [32].

### Ecdysteroid genes

**Figure 3:**
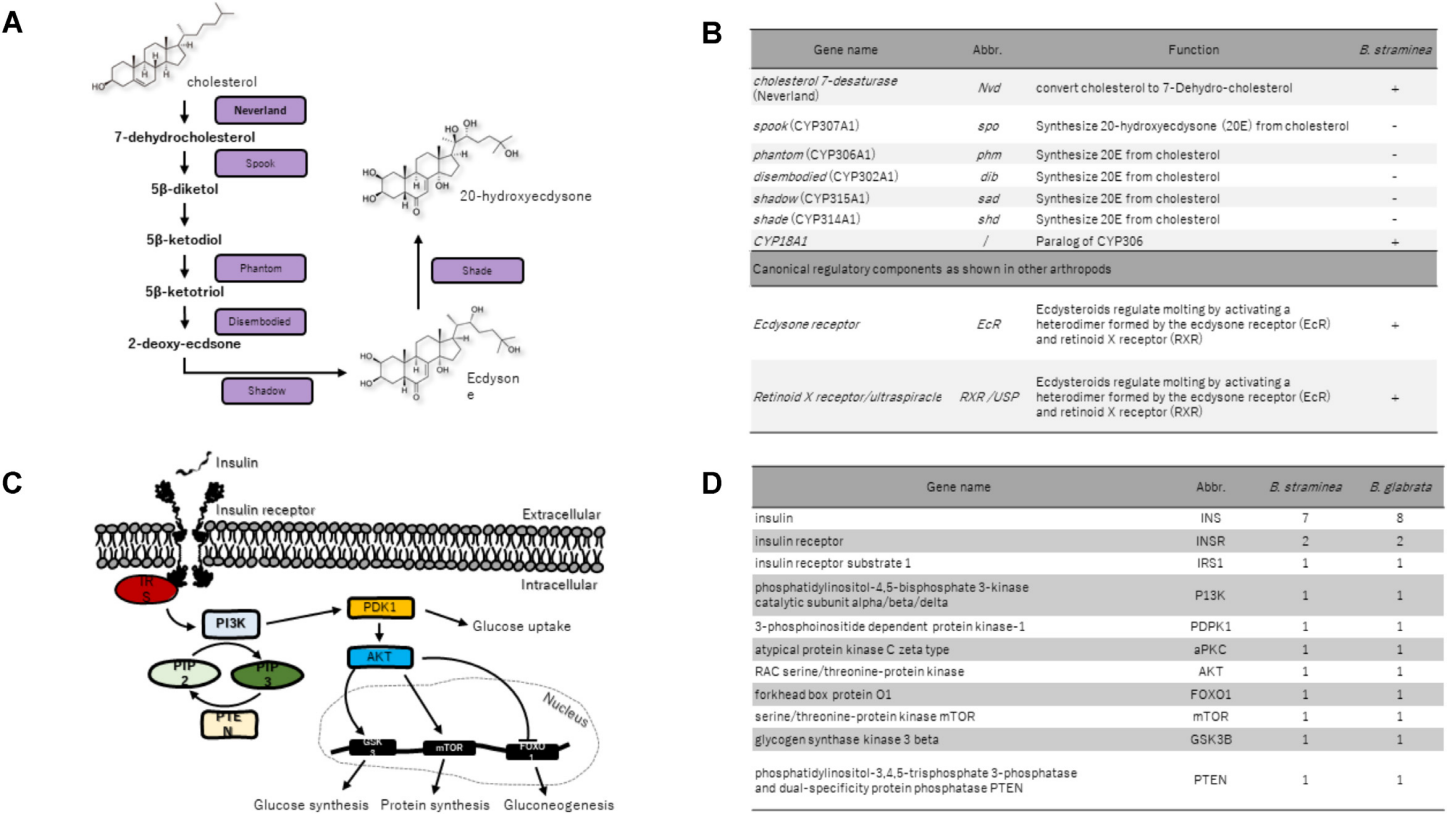
: (A) Schematic diagram of biosynthetic pathway of ecdysteroids; (B) presence and absence of ecdysteroid pathway genes in *B. straminea*; (C) schematic diagram of biosynthetic pathway of insulin; (E) number of gene copies of insulin pathway genes in *B. straminea*.

Ecdysteroids play important roles in regulating growth (in particular molting and metamorphosis) and sexual maturation of insects and other arthropods [33, 34]. Although it has long been known that gastropods contain ecdysteroids and that β-ecdysone could stimulate host location activities in *S. mansoni* miracidia and enhance growth and egg production in *B. glabrata* [35, 36], the biosynthetic pathway genes for ecdysteroids have not been systematically studied in mollusc genomes to date. As shown in Fig. 3A and B, typical genes involved in this pathway including *CYP307A1, CYP306A1, CYP302A1, CYP315A1*, and *CYP314A1* are all absent from the *B. straminea* genome assembly and transcriptome data. Nevertheless, the receptors including EcR, RXR/USP, and oxygenase-like protein Nvd that are essential regulators of cholesterol metabolism are present in *B. straminea* and other mollusc genomes (Fig. 3A and B; Supplementary Information S3). We thus treated *B. straminea* with 10^–6 ^M ecdysteroid 20-hydroxyecdysone for 24 hours but did not observe any significant expression changes in the downstream genes *E74, FOXO*, and *Nvd*. Similar hormone treatments have been shown to elicit the downstream genes in insects in previous studies [37–39]. It is unclear whether only certain forms of ecdysteroids may induce endogenous ecdysteroid pathway genes under particular conditions, and this warrants further investigation. This is the first systematic analysis of ecdysteroid pathway genes in a mollusc genome, thus providing the foundations for future work to determine how ecdysteroids have their effect in these animals.

**Figure 4:**
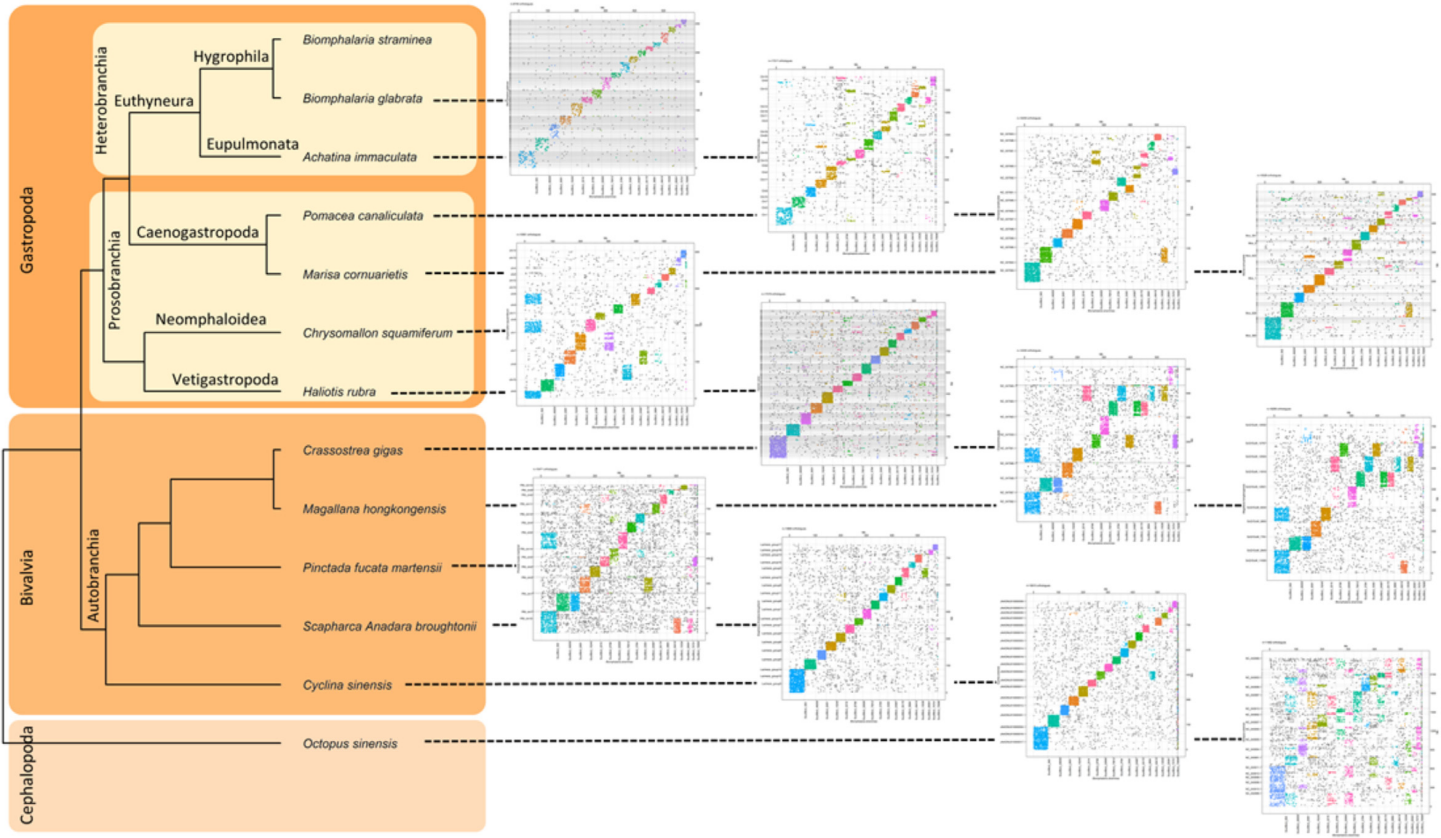
: Synteny between *B. straminea* and 12 mollusc genomes. The species tree is constructed using 2,047 orthogroups with ≥12 of 13 mollusc genomes having single-copy genes in each orthogroup. In the Oxford dot plot, each dot represents a pair of orthologous genes between *B. straminea* and the specific mollusc. Horizontal and vertical dashed lines represent chromosome or scaffold boundaries. Orthologous genes are coloured according to their position in *B. straminea* scaffolds. Significance of synteny blocks is computed using 1-tailed Fisher exact test, and synteny blocks with Benjamini & Hochberg–corrected *P* > 0.05 are indicated in grey.

### Insulin signalling pathway genes

Peptide hormones involved in growth and reproduction have been suggested as candidates for the development of novel methods of schistosomiasis control via manipulation of snail numbers [40]. Insulin is another understudied hormonal pathway in molluscs despite its potential functional roles. For instance, in the pond snail *Lymnaea stagnalis*, a decrease of insulin in the central nervous system correlated with better associative learning behaviour [41], while insulin-related peptides with potential roles in sexual reproduction have been identified in the oyster *C. gigas* [42]. In both *B. straminea* and *B. glabrata* genomes, we were able to identify all key signalling pathway genes (Fig. 3C and D, Supplementary Information S4). This establishes a foundation on which to further explore the functions of these hormones in molluscs.

### Widespread gene turnover between *Biomphalaria* snails and other molluscs

#### Gene gains and losses in mollusc genomes

**Figure 5:**
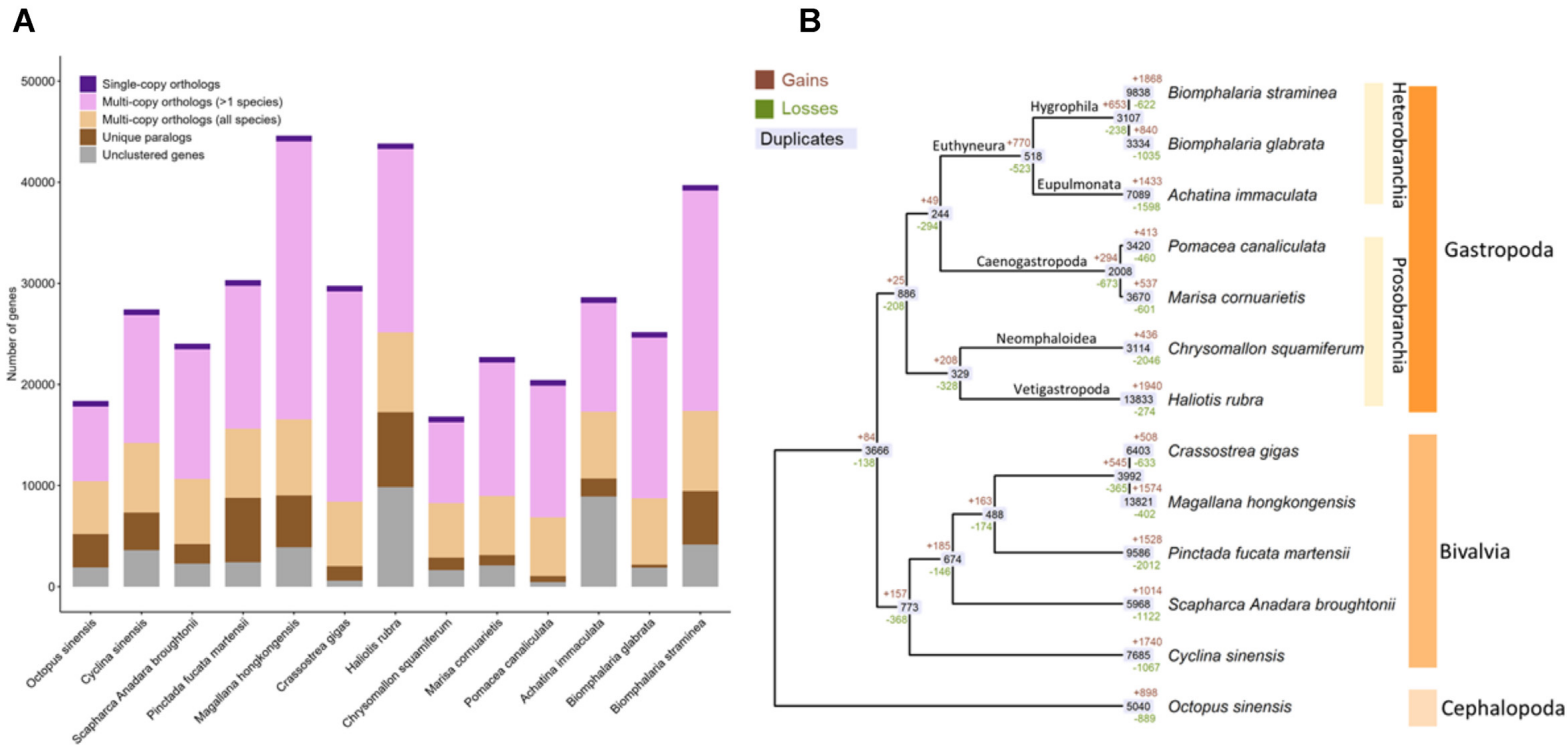
: Summaries of gene families in *B. straminea* and 12 molluscs. (A) Gene family clustering; only the longest isoform for each gene was used. (B) Gene family expansion and contraction between mollusc genomes. Brown and green colour indicate the number of significantly (*P* < 0.05) expanded or contracted gene families at each node, respectively.

A phylogenomic tree was constructed using 2,047 orthogroups with ≥12 of 13 mollusc genomes having single-copy genes in each orthogroup (Supplementary Information S6). Gene family analysis among these genomes revealed the expansion of 1,868 orthogroups and contraction of 622 orthogroups in *B. straminea*, but in *B. glabrata*, the expansion of 840 orthogroups and contraction of 1,035 orthogroups (Fig. 5). These data highlight the importance of having the *B. straminea* genomic resource and potentially suggest that specific control strategies might be needed for *B. straminea* rather than treating it as identical to *B. glabrata*.

#### Expansion of heat shock protein family among mollusc lineages

**Figure 6:**
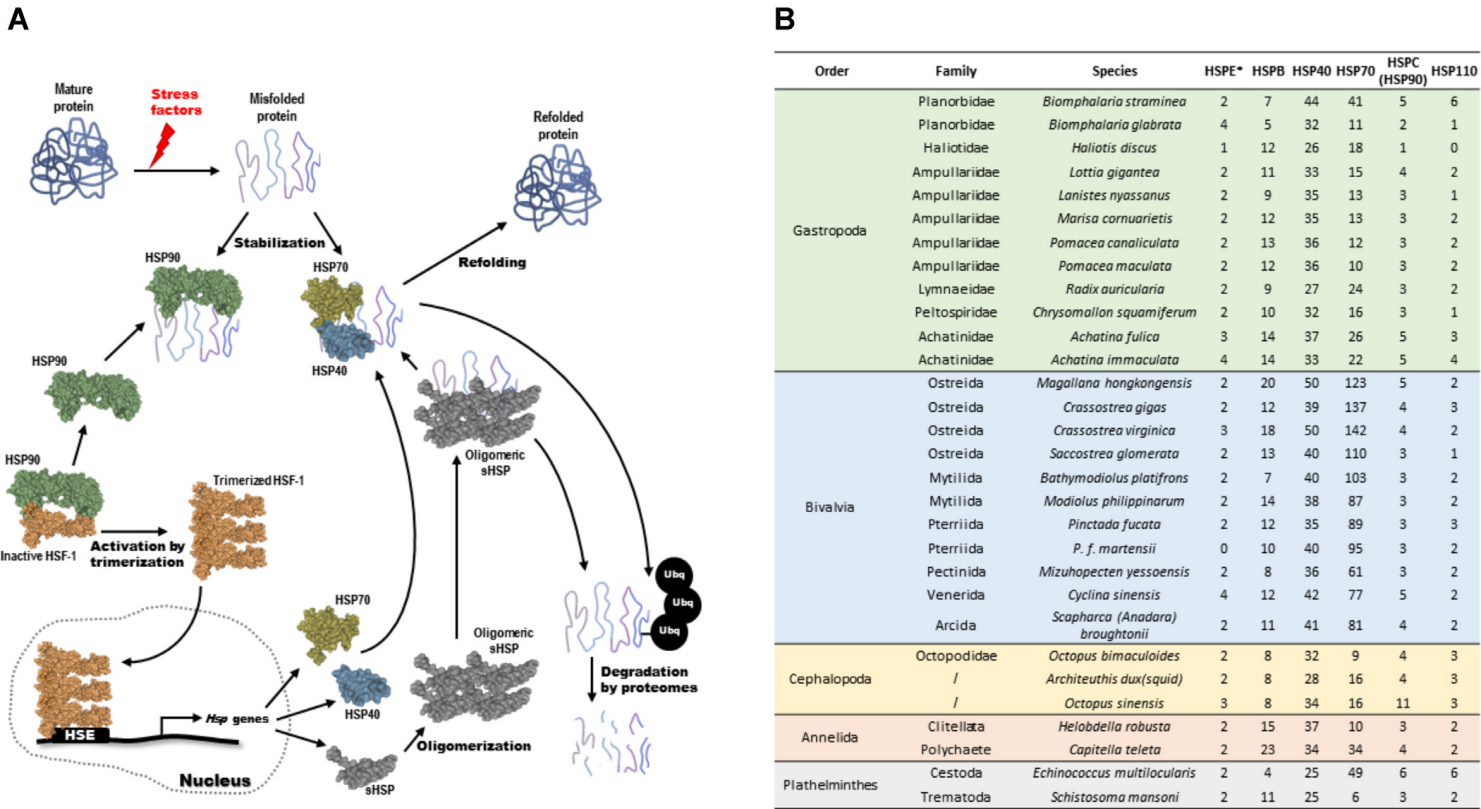
: (A) Schematic diagram showing the heat shock protein actions; (B) number of gene copies of heat shock proteins in different mollusc genomes.

Heat shock proteins are important stress-responsive candidates involved in protein folding for molluscs, activated in response to such things as changing pH, oxygen level, and temperature. In some mollusc genomes, such as that of the Pacific oyster *C. gigas*, an expansion of heat shock protein 70 (HSP70) has been observed in the genome and hypothesized to be important to the animals’ adaptation to changes in ambient environmental factors or pressures [43]. We thus identified the heat shock protein family genes in *Biomphalaria* and compared these to other lophotrochozoans to understand their evolution in different lineages (Fig. 6). Among the different heat shock protein families in the investigated set of gastropods, bivalves, cephalopods, annelids, and platyhelminths, a dramatic expansion is seen specifically in the HSP70 family in the bivalve molluscs (Fig. 6; Supplementary Information S7). Our data and analyses agree with previous studies (e.g., [43]), suggesting that the expansion of HSP70 is linked to the life history of molluscs having a sessile stage. This survey also provides the foundation for future work on the expression and function of particular HSP genes/proteins and their activity in these parasite vectors, which may contribute to their adaptive ability as invasive species and possibly to the recent range expansion of *B. straminea*.

#### Differential sesquiterpenoid and cholesterol genes in certain mollusc lineages

**Figure 7:**
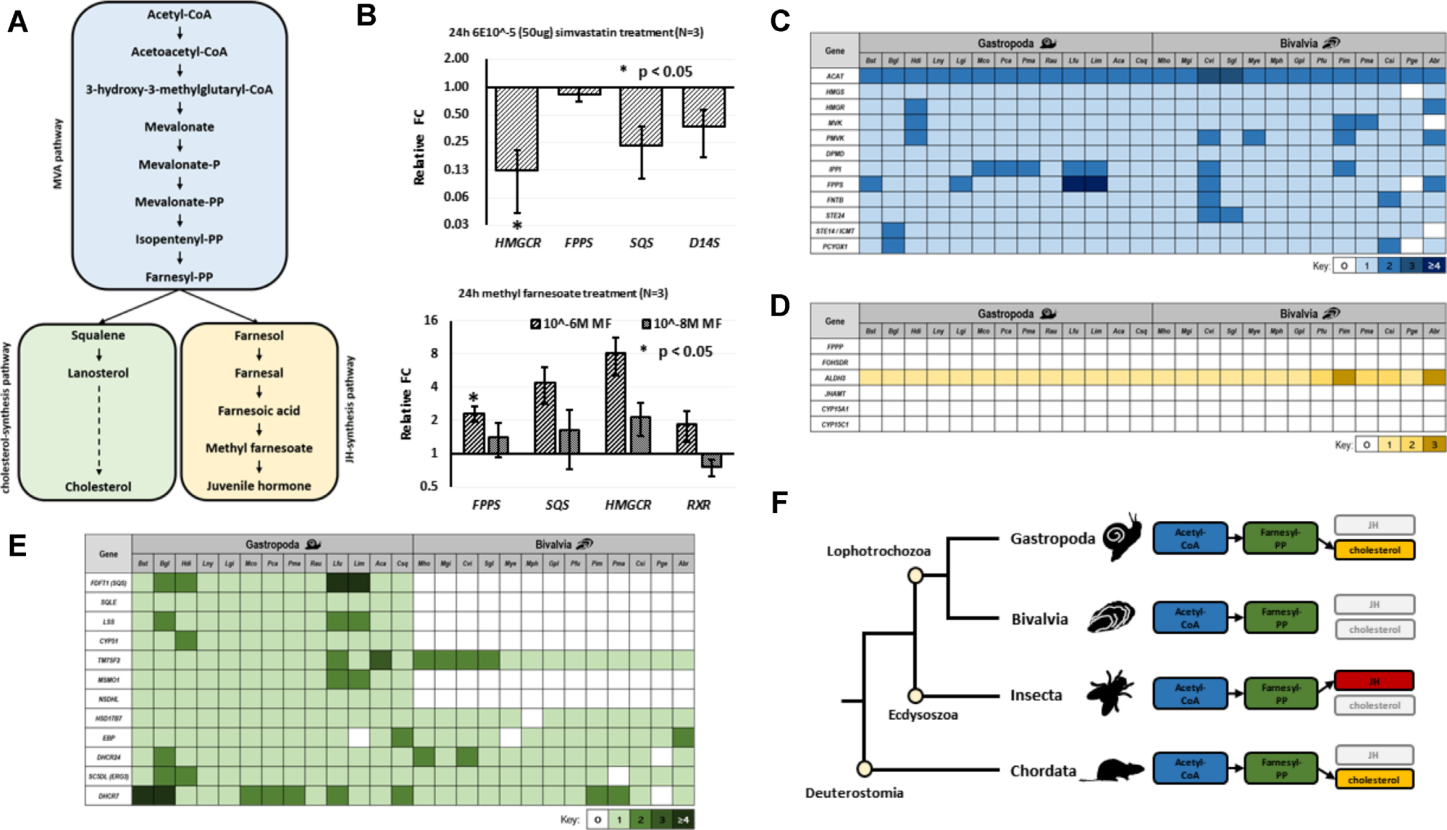
: (A) Schematic diagram showing the mevalonate pathway, and the downstream sesquiterpenoid and *de novo* cholesterol synthesis pathways. (B) Expression of genes upon 6 × 10^–5^M simvastatin, 10^–6^ and 10^–8^ M methyl farnesoate treatment for 24 hours; *P < 0.05. FC: fold change. The bars represent the relative expression level, and the error bars are shown for independently repeated experiments. (C) Heat map of mevalonate pathway orthologues identified in gastropod and bivalve genomes. (D) Heat map of sesquiterpenoid synthesis pathway orthologues identified in gastropod and bivalve genomes. (E) Heat map of *de novo* cholesterol synthesis pathway orthologues identified in gastropod and bivalve genomes. (F) Schematic diagram showing the evolution of sesquiterpenoid pathway genes in bilaterians.

Sesquiterpenoid hormones were once considered specific to insects and crustaceans, where they control development and reproduction [33, 44, 45]. However, recent analyses have shown that the sesquiterpenoid system is also present in myriapods, annelids, and cnidarians [31, 34, 46, 47]. Conversely, vertebrates can only produce cholesterol but not sesquiterpenoids [48, 49], and a recent study revealed the canonical cholesterol biosynthesis pathway in sponges, placozoans, and deuterostomes, suggesting that cnidarians and protostomes experienced massive losses of these genes ([50]; Fig. 7A). Treatment of *B. straminea* with 10^–6 ^M simvastatin and methyl farnesoate changed the expression of sesquiterpenoid pathway genes *HMGCR*and *FPPS*, suggesting a sesquiterpenoid responsive system (Fig. 7B and C). Comparison of sesquiterpenoid pathway genes in mollusc genomes further identified differential utilization of biogenesis pathways in bivalves and gastropods, where only gastropods but not the bivalves are able to produce cholesterol similar to vertebrates (Fig. 7D and F). This is the first systematic study showing the differential sesquiterpenoid and cholesterol synthesis pathways possessed by different mollusc lineages.

## Conclusion

This study presents the first high-quality genome assembly for a schistosomiasis-transmitting snail in China and Asia. The snail *Biomphalaria straminea* is important scientifically, as well as having considerable medical relevance. Our work provides gene and transposable element annotations, and detailed analyses of a variety of gene families, including the homeobox, ecdysteroid, insulin, heat shock protein, and sesquiterpenoid pathway genes, suggesting extensive molecular differences between *B. straminea* and *B. glabrata*, as well as among other molluscan taxa. More generally, our high-quality *B. straminea* genome provides a useful reference point for further understanding of the biology, ecology, and evolution of molluscs.

## Methods

### Sample collection and genome sequencing

One week prior to the experiment, ∼100 ramshorn snails were collected in a freshwater stream in Tai Po New Territories, Hong Kong (GPS: 22.50206300747975, 114.15354682258841). The collected animals were maintained in a laboratory aquarium and fed with lettuce 3 days a week. Samples for genome sequencing originate from single individuals for each sequencing method (Fig. 1A). gDNA was extracted using the PureLink Genomic DNA Mini Kit (Invitrogen, Waltham, Massachusetts, United States) following the manufacturer's protocol. Extracted gDNA was subjected to quality control using a Nanodrop spectrophotometer (Thermo Scientific, Waltham, Massachusetts, United States) and gel electrophoresis. Qualifying samples were sent to Novogene and Dovetail Genomics for library preparation and sequencing. The resulting library was sequenced on an Illumina HiSeq X platform (Illumina HiSeq X, RRID:SCR_016385) to produce 2 × 150 paired-end sequences. The length-weighted mean molecule length is 22.2 kb, and the raw data can be found at NCBI's SRA (SRR12963913).

### Dovetail Omni-C library preparation and sequencing

For each Dovetail Omni-C library, chromatin was fixed with formaldehyde and extracted. Fixed chromatin was digested with DNAse I, and chromatin ends were repaired and ligated to a biotinylated bridge adapter followed by proximity ligation of adapter-containing ends. After proximity ligation, crosslinks were reversed and the DNA was purified. Purified DNA was treated to remove biotin that was not internal to ligated fragments. Sequencing libraries were generated using NEBNext Ultra enzymes and Illumina-compatible adapters. Biotin-containing fragments were isolated using streptavidin beads before PCR enrichment of each library. The library was sequenced on an Illumina HiSeqX platform to produce 128 million 150-bp read pairs, and the raw data can be found at NCBI's SRA (SRR12963914).

### Transcriptome sequencing

Total RNA from different tissues was isolated using a combination method of cetyltrimethylammonium bromide (CTAB) pre-treatment [51] and mirVana™ miRNA Isolation Kit (Ambion, Austin, Texas, United States) following the manufacturer's protocol. The extracted total RNA was subjected to quality control using a Nanodrop spectrophotometer (Thermo Scientific, Waltham, Massachusetts, United States), gel electrophoresis, and an Agilent 2100 Bioanalyzer (Agilent RNA 6000 Nano Kit). Qualifying samples underwent library construction and sequencing at Novogene; polyA-selected RNA-Sequencing libraries were prepared using the TruSeq RNA Sample Prep Kit v2. Insert sizes and library concentrations of final libraries were determined using an Agilent 2100 bioanalyzer instrument (Agilent DNA 1000 Reagents) and real-time quantitative PCR (TaqMan Probe), respectively. Details of the sequencing data can be found in Supplementary Information S1.

### Genome assembly

Chromium whole-genome sequencing reads were used to construct a *de novo* assembly using Supernova (v 2.1.1) with default parameters (raw coverage = 68.32×). The Supernova output pseudohap assembly and Dovetail OmniC library reads were used as input data for HiRise, a software pipeline designed specifically for using proximity ligation data to scaffold genome assemblies [52]. Dovetail OmniC library sequences were aligned to the draft input assembly using BWA [53]. The separations of Dovetail OmniC read pairs mapped within draft scaffolds were analysed by HiRise to produce a likelihood model for genomic distance between read pairs, and the model was used to identify and break putative misjoins, to score prospective joins, and make joins above a threshold.

### Gene model prediction

Gene models were predicted as described in the Hong Kong oyster (*M. hongkongensis*) genome [20]. Briefly, the gene models were trained and predicted using funannotate (v1.7.4 [54]) [55] with the following parameters: “–repeats2evm –protein_evidence uniprot_sprot.fasta –genemark_mode ET –busco_seed_species metazoa –optimize_augustus –busco_db metazoa –organism other –max_intronlen 350 000.” The gene models from several prediction sources including GeneMark, high-quality Augustus predictions (HiQ), PASA, Augustus, GlimmerHM, and SNAP were passed to EvidenceModeler and generated the gene model annotation files, followed by PASA to update the EVM consensus predictions, and add untranslated region annotations and models for alternatively spliced isoforms. Protein-coding genes were searched with BLASTp (BLASTp, RRID:SCR_001010) against the nr and swissprot databases by diamond (v0.9.24) [56] with parameters “–more-sensitive –evalue 1e-3,” and mapped by HISAT2 version 2.1.0 (HISAT2, RRID:SCR_015530) with transcriptome reads [57]. Gene models with no similarity to any known protein and no messenger RNA support were removed from the final version.

### Repetitive element annotation

Repetitive elements were identified using the transposable element annotation pipeline Earl Grey [58] as follows. First, elements were identified using RepeatMasker v.4.1 (RepeatMasker, RRID:SCR_012954) [84], using a sensitive (-s) search and ignoring low-complexity repeats (-nolow). Subsequently, a *de novo* repeat library was constructed using RepeatModeler v.1.0.11 (RepeatModeler, RRID:SCR_015027) [59], including RECON v.1.08 (RECON, RRID:SCR_021170) [60] and RepeatScout v.1.0.5 (RepeatScout, RRID:SCR_014653) [61]. Identified novel repeats were analysed using a “BLAST, Extract, Extend” process to characterize elements along their entire length [62]; consensus sequences and classifications for each repeat family were generated, and the resulting *de novo* repeat library was used to identify repetitive elements in RepeatMasker. All plots were generated using Rstudio ver. 1.2.1335 with R ver. 3.5.1 [63] and ggplot2 ver. 3.2.1 (ggplot2, RRID:SCR_014601) [85].

### Gene family annotation and gene tree building

Gene family sequences were first obtained from NCBI for selected species, including *B. glabrata* and other lophotrochozoans. The sequences were then used to retrieve the corresponding genes from the *B. straminea* genome using the tBLASTn algorithm on a local server, with an E-value of <10^–3^. The identity of each retrieved gene was then checked by reciprocal searches against the Genbank nr database at NCBI with BLASTx. For phylogenetic analyses of gene families, DNA sequences were first translated into amino acid sequences and aligned to other reference sequences (extracted from NCBI) using Clustal W. Gapped sites were removed from alignments using MEGA 7.0 (MEGA, RRID:SCR_000667), and phylogenetic trees (neighbour-joining) were constructed using MEGA 7.0, where each phylogenetic node was analysed using 1,000 bootstrap replicates. For homeobox-containing genes, homeodomains were annotated using tBLASTn searches with HomeoDB sequences, and sequences from representative lophotrochozoan families, including the expanded Spiralia TALEs [21]. We also removed redundant hits based on their unique locations in the genome sequence, and manually detected any likely artefactual duplicates that were not carried forward into the protein sequence alignments (Supplementary Table S2). Alignments of each class were made using MUSCLE (MUSCLE, RRID:SCR_011812) [64], with homeodomain sequences from human (*Homo sapiens*, deuterostome), amphioxus (*Branchiostoma floridae*, a cephalochordate deuterostome), the ecdysozoans fruitfly (*Drosophila melanogaster*) and red flour beetle (*Tribolium castaneum*), and the lophotrochozoans oyster (*Crassostrea gigas*, bivalve), limpet (*Lottia gigantea*, gastropod), brachiopod (*Lingula anatina*), and annelids *Platynereis dumerilii* and *Capitella teleta*, where available from other studies [19, 21] and HomeoDB [86], [87]. The best substitution models were tested with ModelFinder, and maximum likelihood phylogenies were constructed with IQ-TREE (IQ-TREE, RRID:SCR_017254) with 1,000 bootstrap replicates [65].

### Identification of orthologous genes and gene families

Orthologues and orthogroups in *B. straminea* and 12 other animal proteomes were inferred using OrthoFinder v. 2.5.2 (OrthoFinder, RRID:SCR_017118) [66] with default values and “-M msa” activated. To cover the gene families, the longest protein of each gene was taken as the representative in OrthoFinder analysis. Gene duplication events were then identified. Duplication ratios per node/tip were calculated by dividing the number of duplications observed in each node/tip by the total number of gene trees containing that node. CAFE5 was used to infer gene gain and loss rates [80]. Orthogroups from the output of OrthoFinder were regarded as gene families and fed to CAFE5. A divergence tree was inferred using r8s [82] from the species tree generated by OrthoFinder. We tested several gamma rate categories (-*k*), and *k* = 1 showed the best likelihood.

### Functional terms enrichment analysis

Orthogroups were assigned Gene Ontology (GO), EuKaryotic Orthologous Groups (KOG), KEGG, and KEGG Orthology (KO) terms by inheriting the terms from genes found within the groups. The functional term annotations were performed using eggNOG (eggNOG, RRID:SCR_002456) [67]. Functional enrichment was tested for using the function “compareCluster()” in R package “clusterProfiler” v.3.16.1 [68] under the environment of R 4.0.4 [81]. Significantly enriched terms were determined with pvalueCutoff = 0.05, pAdjustMethod = “BH,” and qvalueCutoff = 0.2. Data were visualized using R packages ggplot2 (Wickham 2016), ggtree [69], and “pathview” [70].

### Macrosynteny analysis

Single-copy orthologues anchored by mutual best Diamond blastp v0.9.14.115 [76] hits (–evalue 0.001) between *B. straminea* and 12 other animals with chromosome-level or near chromosome-level assemblies were used in macrosynteny analysis. Oxford synteny plots were generated following previously described methods [83] using the R package ggplot2 (Wickham 2016).

### Drug and hormone treatment and RT-qPCR

Experimental adult animals of ∼1 cm with reproductive capability were isolated from the culture and were rinsed in double-distilled water to remove any contaminants. Three individuals per set were placed in a glass container, with a well of 3.5 cm in diameter and 0.8 cm in depth, filled with 2 mL of double-distilled water with either 10^–6^ or 10^–8^ M of methyl farnesoate (MF) (Sigma), 6 × 10^–5^ M of simvastatin (Sigma), or 10^–6^ M of 20-hydroxyecdysone (Abcam Biochemicals) in separate set-ups. The chemicals were first dissolved in acetone and diluted to the target concentration in the treatment container. The control set-up contained the same number of individuals and was treated with the same concentration of acetone in corresponding experiments. Each replicate of snails was exposed for 24 hours to these treatments without any feeding. Post-treated animals were rinsed with double-distilled water and shells were removed prior to whole-body total RNA extraction. The RNA from each experiment was isolated using TRIzol reagent following the manufacturer's protocol. Purified RNA was dissolved in nuclease-free water. The complementary DNA (cDNA) synthesis was performed using the iScript gDNA Clear cDNA Synthesis Kit (BioRad) following the manufacturer's protocol. The cDNA was used in subsequent quantitative real-time PCR. The amplification conditions were as follows: initial denaturation at 95°C for 30 s, followed by 40 cycles of 95°C denaturation for 15 s, 57°C primer annealing for 15 s, and 72°C extension for 15 s. Primer details are listed in Supplementary File S8. The primers were tested by conventional PCR with *B. straminea* cDNA prior to experiments to ensure their specificity. Each sample was analysed in replicates. The expression of each target gene transcript was normalized to the housekeeping gene, myoglobin (*Myo*), as adopted in previous studies [71–74]. The subsequent fold induction analyses were calculated using the ΔΔCt method.

## Data Availability

The raw genome and RNA sequencing data have been deposited in the SRA under Bioproject No. PRJNA673593. The final chromosome assembly was submitted to NCBI Assembly under accession No. JADKLZ000000000. All data can also be found in the GigaScience Database [75].

## Additional Files

**Supplementary Information S1**. Sequencing data.

**Supplementary Information S2**. (a) Tables of homeobox gene sequences in *B. straminea, B. glabrata*, a synteny comparison of homeobox genes, and comparison of ParaHox gene linkage. (b) Distribution of homeoboxes in the genome of *Biomphalaria glabrata*. (c) Alignments and phylogenies of each class of homeobox sequences.

**Supplementary Information S3**. Ecdysteroid genes.

**Supplementary Information S4**. Insulin pathway genes.

**Supplementary Information S5**. Synteny information.

**Supplementary Information S6**. Gene expansion and contraction.

**Supplementary Information S7**. Heat shock protein family genes.

**Supplementary Information S8**. Cholesterol genes and primers.

**Supplementary Information S9**. Phylogenetic trees.

**Supplementary Information S10**. Tables.

giac012_GIGA-D-21-00243_Original_Submission

giac012_GIGA-D-21-00243_Revision_1

giac012_GIGA-D-21-00243_Revision_2

giac012_Response_to_Reviewer_Comments_Revision_1

giac012_Reviewer_1_Report_Original_Submission Jacob Tennessen -- 8/28/2021 Reviewed

giac012_Reviewer_2_Report_Original_Submission Coen Adema -- 9/5/2021 Reviewed

giac012_Reviewer_2_Report_Revision_1 Coen Adema -- 1/5/2022 Reviewed

giac012_Supplemental_Files

## Abbreviations

BLAST: Basic Local Alignment Search Tool; BUSCO: Benchmarking Universal Single-Copy Orthologs; BWA: Burrows-Wheeler Aligner; cDNA: complementary DNA; gDNA: genomic DNA; kb: kilobase pairs; KEGG: Kyoto Encyclopedia of Genes and Genomes; LINE: long interspersed nuclear element; LTR: long terminal repeat; Mb: megabase pairs; NCBI: National Center for Biotechnology Information; PASA: Program to Assemble Spliced Alignments; SINE: short interspersed nuclear element; SRA: Sequence Read Archive.

## Competing Interests

The authors declare that they have no competing interests.

## Funding

This work was supported by the Hong Kong Research Grant Council Collaborative Research Fund(C4015-20EF), General Research Fund (14100919), NSFC/RGC Joint Research Scheme (N_CUHK401/21), and The Chinese University of Hong Kong Direct Grant (4053433, 4053489). Y.Y., W.L.S., C.F.W., S.T.S.L., and Y.L. were supported by the Ph.D. studentships of The Chinese University of Hong Kong. A.H. is supported by a Biotechnology and Biological Sciences Research Council (BBSRC) David Phillips Fellowship (BB/N020146/1). T.B. is supported by a studentship from the Biotechnology and Biological Sciences Research Council-funded South West Biosciences Doctoral Training Partnership (BB/M009122/1). M.E.A.R. is supported by a Ph.D. studentship from the School of Biology and St Andrews University.

## Conflict of interest

The authors declare no conflict of interests.

## Authors’ Contributions

J.H.L.H., D.E.K.F., A.H., Z.W., S.X., Z.P.K., and S.S.C. conceived the study. J.H.L.H., D.E.K.F., and A.H. supervised the study. W.N., J.H., and T.S. assembled the genome. W.N. carried out the gene model prediction and comparison. Y.Y. carried out the heat shock protein analyses. Y.X. carried out the gene gain and loss and synteny analyses. W.L.S. and C.F.W. carried out the sesquiterpenoid analyses. Y.Y., W.L.S., and S.Y.L. carried out the ecdysteroid analyses. M.E.A.R. and Y.L. carried out the homeobox gene analyses. T.B. carried out the transposable element analyses. S.T.S.L. carried out the insulin analyses. W.N., Y.Y., Y.X., W.L.S., M.E.A.R., T.B., A.H., D.E.K.F., and J.H.L.H. wrote the first draft of manuscript. All authors approved the final version of the manuscript.
