# Supplementary material for: Genome of the ramshorn snail Biomphalaria straminea—an obligate intermediate host of schistosomiasis
Source: Gigascience. 2022 Feb 15;11:giac012. doi: 10.1093/gigascience/giac012 (PMC8848322; doi:10.1093/gigascience/giac012)
Supplement: giac012_GIGA-D-21-00243_Revision_1 [file giac012_giga-d-21-00243_revision_1.pdf]

GigaScience

Genome of the ramshorn snail Biomphalaria straminea - an obligate intermediate host of schistosomiasis

--Manuscript Draft--

**Manuscript Number:**	GIGA-D-21-00243R1
**Full Title:**	Genome of the ramshorn snail Biomphalaria straminea - an obligate intermediate host of schistosomiasis
**Article Type:**	Research
**Funding Information:**	Hong Kong Research Grant Council (Collaborative Research Fund (C4015-20EF), General Research Fund (14100919)) — Dr. Jerome Hui
**Abstract:**	Background<br>Schistosomiasis or bilharzia is a parasitic disease caused by trematode flatworms of the genus Schistosoma. Infection of Schistosoma mansoni in humans results when cercariae emerge into water from freshwater snails in the genus Biomphalaria, and seek out and penetrate human skin. The snail Biomphalaria straminea is native to South America and is now also present in Central America and China, and represents a potential vector host for spreading schistosomiasis. To date, genomic information for the genus is restricted to the neotropical species Biomphalaria glabrata. This limits understanding of the biology and management of other schistosomiasis vectors, such as B. straminea.<br>Findings<br>Using a combination of Illumina short-read, 10X Genomics linked-read, and Hi-C sequencing data, our 1.005 Gbp B. straminea genome assembly is of high contiguity, with a scaffold N50 of 25.3 Mbp. Transcriptomes from adults were also obtained. Developmental homeobox genes, hormonal genes, and stress-response genes were identified, and repeat content was annotated (40.68% of genomic content). Comparisons with other mollusc genomes (including Gastropoda, Bivalvia and Cephalopoda) revealed syntenic conservation, patterns of homeobox gene linkage indicative of evolutionary changes to gene clusters, expansion of heat shock protein genes, and the presence of sesquiterpenoid and cholesterol metabolic pathway genes in Gastropoda. In addition, hormone treatment together with RT-qPCR assay reveal a sesquiterpenoid hormone responsive system in B. straminea, illustrating this renowned insect hormonal system is also present in the lophotrochozoan lineage.<br>Conclusion<br>This study provides the first genome assembly for the snail B. straminea and offers an unprecedented opportunity to address a variety of biology related to snail vectors of schistosomiasis, as well as evolutionary and genomics questions related to molluscs more widely.
**Corresponding Author:**	Jerome Hui<br><br>HONG KONG
**Corresponding Author Secondary Information:**	
**Corresponding Author's Institution:**	
**Corresponding Author's Secondary Institution:**	
**First Author:**	Wenyan Nong
**First Author Secondary Information:**	
**Order of Authors:**	Wenyan Nong
	Yifei Yu
	Madeleine E. Aase-Remedios

Powered by Editorial Manager® and ProduXion Manager® from Aries Systems Corporation

	Yichun Xie
	Wai Lok So
	Yiqian Li
	Cheuk Fung Wong
	Toby Baril
	Sean TS Law
	Sheung Yee Lai
	Jasmine Haimovitz
	Thomas Swale
	Shan-shan Chen
	Zhen-peng Kai
	Xi Sun
	Zhongdao Wu
	Alexander Hayward
	David Ferrier
	Jerome Hui
**Order of Authors Secondary Information:**	
**Response to Reviewers:**	The file is attached.
**Additional Information:**	
**Question**	**Response**
Are you submitting this manuscript to a special series or article collection?	No
**Experimental design and statistics**<br><br>Full details of the experimental design and statistical methods used should be given in the Methods section, as detailed in our Minimum Standards Reporting Checklist. Information essential to interpreting the data presented should be made available in the figure legends.<br><br>Have you included all the information requested in your manuscript?	Yes
**Resources**<br><br>A description of all resources used, including antibodies, cell lines, animals and software tools, with enough information to allow them to be uniquely identified, should be included in the	Yes

Methods section. Authors are strongly encouraged to cite Research Resource Identifiers (RRIDs) for antibodies, model organisms and tools, where possible.<br><br>Have you included the information requested as detailed in our Minimum Standards Reporting Checklist?	
**Availability of data and materials**<br><br>All datasets and code on which the conclusions of the paper rely must be either included in your submission or deposited in publicly available repositories (where available and ethically appropriate), referencing such data using a unique identifier in the references and in the “Availability of Data and Materials” section of your manuscript.<br><br>Have you have met the above requirement as detailed in our Minimum Standards Reporting Checklist?	Yes

Manuscript

Click here to access/download;Manuscript;Snail genome (Changes highlighted) (1).docx

Genome of the ramshorn snail *Biomphalaria straminea* - an obligate intermediate host of schistosomiasis

Wenyan Nong[1,^], Yifei Yu[1,^], Madeleine E. Aase-Remedios[3,^], Yichun Xie[1,^], Wai Lok So[1,^], Yiqian Li[1,^], Cheuk Fung Wong[1,^], Toby Baril[2,^], Sean T. S. Law[1,^], Sheung Yee Lai[1], Jasmine Haimovitz[4], Thomas Swale[4], Shan-shan Chen[5], Zhen-peng Kai[6], Sun Xi[7], Zhongdao Wu[7], Alexander Hayward[2,*], David E.K. Ferrier[3,*], Jerome H.L. Hui[1,*]

1. School of Life Science, Simon F.S. Li Marine Science Laboratory, State Key Laboratory of Agrobiotechnology, The Chinese University of Hong Kong, Hong Kong, China

2. University of Exeter, United Kingdom

3. The Scottish Oceans Institute, Gatty Marine Laboratory, School of Biology, University of St. Andrews, United Kingdom

4. Dovetail Genomics, United States of America

5. Institute of Agro-food Standard and Testing Technology, Shanghai Academy of Agricultural Sciences, Shanghai, China

6. School of Chemical and Environmental Engineering, Shanghai Institute of Technology, Shanghai, China

7. Sun Yat-sen University, Guangdong, China

^ = contributed equally;

*=correspondence:

alex.hayward@exeter.ac.uk; dekf@st-andrews.ac.uk; jeromehui@cuhk.edu.hk

Abstract

Background

Schistosomiasis or bilharzia is a parasitic disease caused by trematode flatworms of the genus *Schistosoma*. Infection of *Schistosoma mansoni* in humans results when cercariae emerge into water from freshwater snails in the genus *Biomphalaria*, and seek out and penetrate human skin. The snail *Biomphalaria straminea* is native to South America and is now also present in Central America and China, and represents a potential vector host for spreading schistosomiasis. To date, genomic information for the genus is restricted to the neotropical species *Biomphalaria glabrata*. This limits understanding of the biology and management of other schistosomiasis vectors, such as *B. straminea*.

Findings

Using a combination of Illumina short- read, 10X Genomics linked- read, and Hi- C sequencing data, our 1.005 Gbp *B. straminea* genome assembly is of high contiguity, with a scaffold N50 of 25.3 Mbp. Transcriptomes from adults were also obtained. Developmental homeobox genes, hormonal genes, and stress-response genes were identified, and repeat content was annotated (40.68% of genomic content). Comparisons with other mollusc genomes (including Gastropoda, Bivalvia and Cephalopoda) revealed syntenic conservation, patterns of homeobox gene linkage indicative of evolutionary changes to gene clusters, expansion of heat shock protein genes, and the presence of sesquiterpenoid and cholesterol metabolic pathway genes in Gastropoda. In addition, hormone treatment together with RT-qPCR assay reveal a sesquiterpenoid hormone responsive system in *B. straminea,* illustrating this renowned insect hormonal system is also present in the lophotrochozoan lineage.

Conclusion

This study provides the first genome assembly for the snail *B. straminea* and offers an unprecedented opportunity to address a variety of biology related to snail vectors of schistosomiasis, as well as evolutionary and genomics questions related to molluscs more widely.

Background

With over 240 million people worldwide estimated to require treatment, the World Health Organisation considers schistosomiasis to be the second most prevalent parasitic disease after malaria (https://www.who.int/health-topics/schistosomiasis). As such, schistosomiasis is a global health problem that causes considerable economic and social burdens.

Infection by *Schistosoma mansoni* in human results when cercariae emerge into the water from their freshwater snail intermediate hosts in the genus *Biomphalaria*, and seek out and penetrate submerged body parts through the skin. Once inside the human body, adult worms lay eggs, which are deposited in the blood venules and will cross the intestinal wall to leave the body in the faeces. In the meantime, eggs that fail to cross the wall (named "reflux eggs") circulate to the liver where they grow, emerge, and cause pathology. Miracidia larvae hatch from eggs that reach water, then seek out and penetrate a new snail intermediate host. Following this, sporocysts develop in the infected snails, and subsequently free-living cercariae emerge from the snail into the water, completing the parasitic life cycle. Among the 34 described species of *Biomphalaria* snails, 18 species (including *B. straminea*) have been demonstrated to be the potential vectors for *S. mansoni*. Different geographical locations are dominated by different species of *Biomphalaria*.

The native range of *Biomphalaria* snails is South America and Africa (Campbell et al 2000; DeJong 2001). However, several species have been introduced to other areas, presenting a risk of schistosomiasis infection. The occurrence of *B. straminea* in Asia was first reported at Lam Tsuen valley in Hong Kong during the 1970s (Meier-Brook 1974; Figure 1A), presumably having somehow spread from its native range in South America into Central America and southern China (Yang et al., 2018). *B. straminea* have since been identified at a number of locations in Hong Kong and Guangdong Province (Attwood et al 2015; Dudgeon and Yipp 1983; Meier-Brook 1974; Woodruff et al 1985; Zeng et al 2017). While *S. mansoni* is not yet endemic in either Hong Kong or mainland China, cases of schistosomiasis caused by the parasite are currently increasing in China. According to the records from the database of the National Notifiable Disease Report System (NNDRS), 355 cases of imported schistosomiasis cases had been reported in 15 provinces in China between 1979 and 2019, including 78 cases infected with *S. mansoni*, 262 cases with *S. haematobia*, and 15 cases with unidentified *Schistosoma*. Since *B. straminea* had already been discovered in Guangdong province in southern China, it is believed that the imported *S. mansoni* increases the risk of its transmission in China. (Zhu and Xu 2014; Wang et al 2020).

Whole genome sequences are valuable resources for obtaining deeper understanding of the biology of any organism. Despite the importance of the phylum Mollusca, there is a lack of genomic resources (Davison and Neiman 2021). In the case of *B. straminea*, such a resource will impact questions of how they may interact with *S. mansoni* and how similar the genetic mechanisms are between different *Biomphalaria* species, with possible implications for how treatments and management strategies might be transferable. To date, only the genome of *Biomphalaria glabrata* has been sequenced and analysed (Adema et al 2017; Tennessen et al 2020; Figure 1B), and a high-quality genome of *B. straminea* is lacking, hindering further understanding

of the species. To address this issue, we provide and analyse a high-quality genome assembly for *B. straminea* together with accompanying transcriptomes.

Results and Discussion

Genome quality evaluation

Genomic DNA was extracted from single individuals of *B. straminea* (Figure 1A). Genome sequences were first assembled using short-reads followed by scaffolding with Hi-C data. The genome assembly (without the mitochondrial genome) is 1.005 Gbp with a scaffold N50 of 25.3 Mbp (Figure 1B). This high physical contiguity is matched by high completeness, with an 87.0% complete BUSCO score (Simao et al 2015)(Figure 1B). A total of 43,340 gene models, including 3,122 tRNA and 40,218 protein-coding genes, were generated by mapping transcriptome data to the genome assembly (S1. Sequencing data). The mean exon length is 262 bp, mean intron length is 1,603 bp, and mean deduced protein length is 377 aa. The genome quality generated in this study is comparable to the previously published genome assemblies of another schistosomiasis carrying vector snail, *B. glabrata* (Adema et al 2017; Tennessen et al 2020; Figure 1B).

Repeat element analysis

We identified a total repeat content of 40.68% in the genome of *B. straminea* (Figure 1C), demonstrating that repeats make up a large proportion of total genome size in the species. A considerable proportion of repeats were unclassified (15.81%), suggesting that many of the annotated repeats represent new repeat families (Figure 1C), which is not unexpected given the relatively sparse attention given to the analysis of repeats in gastropod molluscs to date. Of the remaining repeats, LINE elements and DNA transposons are most abundant (LINEs: 10.48%, DNA transposons: 8.32%), whereas SINEs, LTR elements, and rolling-circle elements are present only in low proportions (LTR elements: 2.7%, rolling-circle elements: 1.71%, SINEs: 1.31%) (Figure 1C). Consideration of a repeat landscape plot suggests that there has been a long-term ongoing expansion of repeats in *B. straminea,* with a recent spike in activity. The recent spike is

evident from the relatively large percentage of repeats in the genome that are separated from their family consensus sequences by short distances, while the long tail of increasing divergence from consensus is suggestive of a gradual increase in activity of a relatively long time period (Figure 1C). LINEs and DNA transposons have expanded most significantly, however, there has also been a less considerable expansion of LTR and Rolling circle elements (Figure 1C).

Homeobox-containing gene content and linkage

a) Hox cluster genes

Homeobox genes are transcription factors involved in regulating animal development. Not only are they highly conserved between distantly related lineages, but also many of the genes are linked or clustered in genomes. Besides the most well-known clusters like the Hox and ParaHox clusters, many homeobox genes are linked including other ANTP class genes in NK and SuperHox clusters, and also amongst other classes of PRD, TALE, and SINE homeobox genes (Butts et al., 2008; Mazza et al., 2010; Ferrier, 2016). These clusters have been maintained or dispersed differently in different animal lineages. Changes to gene clustering may represent the breakdown of regulatory constraints which normally maintain clusters and are thought to be the mechanism holding together the tightly regulated Hox cluster, for instance. Genomic clustering also reflects the ancient origins of many of these homeobox genes by tandem duplication, e.g., the four ANTP clusters in the Bilaterian ancestor that arose via subsequent expansions from a single Proto-ANTP gene (Hui et al., 2012). Among molluscs, a diverse phylum to which gastropods belong, alongside other conchiferans (monoplacophorans, bivalves, scaphopods, and cephalopods), as well as aculiferans (aplacophorans and polyplacophorans), some of the diversity of body plans may be underpinned by changes to developmental genes like homeobox genes. Hox genes have been co-

opted to the development of novel morphological structures in cephalopods (Lee et al., 2003), and this corresponds to a breakdown of the Hox cluster across several chromosomes, and the loss of a few genes (Albertin et al., 2015). Other mollusc genomes show a breakdown of homeobox clustering overall, like the Pacific oyster (*Crassostrea gigas*; Paps et al., 2015), while a more recent chromosome-level assembly reveals large-scale patterns of linkage in *Magallana hongkongensis* (Li et al., 2020). This genome assembly of *B. straminea* improves our understanding of homeobox gene linkage in comparison to other molluscs, which are lophotrochozoans and, alongside and well-studied ecdysozoans like flies, provide a more thorough protostome comparison to vertebrates, which are within the Deuterostomia.

We found 114 homeobox genes in the genome of *B. straminea*, belonging to eleven recognized classes and one lophotrochozoan-specific gene, *Lopx* (Supplementary information S2a; Barton-Owen et al., 2018). Many of these genes are clustered (situated on the same chromosome with no or very few non-homeobox genes in between) or linked (on the same chromosome, but with intervening non-homeobox genes) in the genome (Figure 2). Nine of the eleven Hox genes are found on scaffold 32695, in an arrangement that suggests several intrachromosomal rearrangements. In an ordered cluster as seen in the gastropod, *L. gigantea*, for instance, the Hox genes are situated in the genome in the ancestral bilaterian order from anterior-acting *Hox1* to posterior-acting *Post1*, and no other non-Hox genes are found amongst the Hox genes (Simakov et al., 2013). Here, however, we find that *Hox2*, *Hox3*, and *Hox4* are downstream of *Hox5*. In addition, *Hox2-Hox5* are downstream of the posterior half of the cluster, including *Lox5*, *Hox7*, *Lox4*, *Lox2*, and *Post1*. *Hox1* is found on another scaffold, while the sequence for *Post2* is not in the genomic assembly, though its sequence is found in our transcriptome data. The Hox arrangement in *B. straminea* provides more linkage information than the *B. glabrata* assembly,

where the short scaffolds corroborate only fragments of the Hox cluster like the linkage of *Hox4*, *Hox3*, and *Hox2*, but do not confirm the rearrangements in *B. straminea*, such as the linkage of *Hox5* to *Hox2* (Supplementary information S2b). We do see a difference in the arrangement of the posterior half of the Hox cluster, however, where in *B. glabrata*, *Lox4*, *Lox2*, *Post2*, and *Post1* are linked in that order on scaffold 139, with *Lox4* and *Lox2* in the negative strand and *Post2* and *Post1* on the positive, which is slightly different from many other molluscs in which only *Post1* differs in orientation relative to the remainder of the posterior end of the Hox cluster genes (Simakov et al., 2013; Li et al., 2020). In *B. straminea*, there has been a rearrangement separating *Post1*, placing it with *Lox5* and *Hox7* and in the same orientation as *Lox4* and *Lox2* (Figure 2). Thus, the Hox genes of *Biomphalaria* seem highly rearranged relative to the ancestral order and each other. Clearly then, there are no (or minimal) long-range regulatory mechanisms operating across these genes that could have constrained their organization and prevented rearrangement. At most, there may be remains of some form of sub-cluster mechanisms, such as enhancer sharing, operating over the small regions (i.e. *Hox2-4* and *Lox2-4*) whose similar arrangement may be indicative of constraints conserved across *Biomphalaria* species. Future expression and regulatory element analyses may help resolve this possibility.

b) ParaHox cluster genes

The ParaHox cluster is the evolutionary sister to the Hox cluster (Brooke et al., 1998). The homeodomains of the three ParaHox genes (*Gsx, Xlox* and *Cdx*) are found on three separate scaffolds in *B. straminea* (Figure 2), however, three upstream exons of *Cdx* are on scaffold 5393, which also has the *Xlox* gene (Supplementary information S2a). This is in contrast to the genome of *B. glabrata*, where *Gsx* and *Xlox* are linked on scaffold 3 (Supplementary information S2a-b).

Perhaps this pattern reflects maintained linkage between all three ParaHox genes in *Biomphalaria* species and only because of the draft level of all the assemblies this is not evident. However, if this is the case, the ParaHox genes are separated by large amounts of sequence and have not retained the ancestral order of *Gsx-Xlox-Cdx*. *B. glabrata Xlox* is nearly 4 Mb from the start of its scaffold, while in *B. straminea*, *Xlox* is at a location with another homeobox-containing gene (*Phox*)15 Mb away on one side and the first three *Cdx* exons are almost 5 Mb away on the other side of *Xlox*. Thus, although the *Biomphalaria* ParaHox genes may be linked, they cannot be considered to be clustered. This dispersal of ParaHox genes is typical for molluscs in general, with several species also showing loose linkage of some of the genes (Li et al., 2020), which contrasts with the relatively tight clustering of these genes in many deuterostomes (Osborne et al., 2009; Ikuta et al., 2013; Zhang et al., 2017) and the likely pan-cluster regulation that may operate in these deuterostomes.

c) ANTP-class homeobox genes

Beyond Hox and ParaHox, there are other linkages among and between the classes of homeobox genes that hint at their ancient evolutionary origins and genomic arrangement in clusters. Despite the many rearrangements to the Hox cluster, many genes linked to Hox clusters in other species are also found on the same scaffold in *B. straminea*, including *Mnx*, *Gbx-a* and *Gbx-b*, *En-a*, *Evx-a* and *Evx-b*, and *Dlx* (Castro and Holland, 2003; Chourrout et al., 2006; Butts et al., 2008; Hui et al., 2012; Li et al., 2020). These linkages give further support for the hypothesized Super-Hox cluster of non-Hox ANTP-class genes linked to the Hox genes in bilaterians (Butts et al., 2008).

d) SINE homeobox genes

Another highly conserved cluster besides Hox and ParaHox is the SINE-class cluster, typically composed of the *Six3/6*, *1/2*, and *4/5* genes or their protostome orthologues (Ferrier, 2016). In *B. straminea*, *Six4/5* and *Six1/2* are on the same scaffold, but with a number of genes between them, and *Six3/6* is on a distinct scaffold (Figure 2). In *B. glabrata*, *Six3/6* is linked to *Hlx* (Figure S2b), the last homeobox gene at the end of the *Six4/5*-*Six1/2* scaffold in *B. straminea* (Figure 2). Thus, there is clearly not a SINE-class gene cluster conserved in *B. straminea*, but the linkage of at least some of these genes indicates that the dispersal of this cluster has not yet proceeded to the extent of these genes being separated onto different chromosomes. Also, the location of the *Hlx* gene relative to different *Six* genes indicates a certain degree of genomic rearrangement between the two *Biomphalaria* species (i.e. conserved macrosynteny, but divergent microsynteny).

e) IRX homeobox genes

Homeobox genes in the IRX family within the TALE class, are also observed to be clustered in several lineages, for instance the three-gene (*ara*, *caup*, and *mirr*) cluster in *Drosophila*, two three-gene clusters in vertebrates, and four genes in the limpet *L. gigantea* (*irx4*, *irx2*, *irx1*, and *irx3*) (Irimia et al. 2008; Takatori et al., 2008; Kerner et al. 2009). These clusters are thought likely to have arisen convergently by independent tandem duplications in the arthropod, vertebrate, and mollusc lineages (Irimia et al., 2008; Takatori et al., 2008; Kerner et al., 2009; Chipman et al., 2014). Both *Biomphalaria* species have five IRX-family genes, one pair of which appears to be a product of a more recent, possibly *Biomphalaria*-specific, duplication (*Irx1-a* and *Irx1-b*). Perhaps surprisingly, none of the *Biomphalaria Irx* genes, *Irx1* (*a* and *b*), *Irx2*, *Irx3*, and *Irx4*, show clear orthology to specific gastropod (limpet) or bivalve (oyster) genes in a phylogenetic tree

(Supplementary information S2c). A paucity of phylogenetically-informative amino-acid changes is the most likely explanation for this lack of resolution. Despite this lack of resolution of *Irx* orthology across species the *B. straminea* genome assembly does provide a new example of *Irx* gene clustering. *Irx3, Irx2,* and *Irx4* are closely clustered in the genome, while *Irx1-b* is 7 Mb away on the same scaffold, also with *Zhx*, a ZF-class gene another 6 Mb further. The two *Irx1* paralogues, however, are on separate scaffolds, which may represent either a rearrangement following their duplication, convergence of the sequence of the homeodomain, or thirdly, an assembly artefact. In *B. glabrata*, only the linkage of *Irx4* with *Irx2* is corroborated due to the shorter scaffold lengths of that assembly. Further work, perhaps using other conserved domains from these genes and with a wider breadth of lophotrochozoan species could potentially determine whether in fact the four *Irx* gene types in *Biomphalaria* species are orthologous to genes in other species' *Irx* clusters. A multi-gene IRX-family cluster in *Biomphalaria* species with evidence of at least one independent expansion (*Irx1-a* and *Irx1-b*) provides an interesting addition to our understanding of IRX-family clusters, and the mechanisms behind gene expansions and subsequent maintenance of clustering in general.

f) PRD- and LIM- class homeobox genes

We also observe linkages amongst PRD-class genes, with clusters on scaffolds 13536, 2216, 46009, and 563 (Figure 2). The broadly distributed PRD-class cluster is the so-called HRO cluster, composed of the genes *Otp, Rx/Rax* and *Hbn/Arx-like* (Mazza et al., 2010; Ferrier, 2016), which ancestrally was likely embedded within a more extensive PRD/LIM-class mega-cluster, including the PRD-class genes *Gsc* and *Otx* and the LIM-class gene *Isl* (Ferrier, 2016). In *B. straminea* there is a remnant of the HRO cluster, with *Otp* clustered with *Hbn,* internally on a large

scaffold (563) and flanked by other homeobox genes (Figure 2) including another PRD-class gene (*Arx-a*) now in this *Biomphalaria* PRD-class cluster, but the *Rax* genes are on other scaffolds. Interestingly, the *Isl* gene is also on this large 563 scaffold in *B. straminea*, consistent with descent from the hypothesized PRD/LIM-class mega-cluster (Ferrier, 2016). *B. glabrata* provides an interesting contrast as the HRO cluster is now complete (with *Otp, Hbn and Rax-b*) in contrast to *B. straminea*, and again *Arx-a* is also in the *Biomphalaria* cluster (Figure 2; Supplementary Figure S2b). Why the PRD-class HRO cluster would remain intact in one species of *Biomphalaria* but not the other remains to be resolved. Also, whether the inclusion of the *Arx-a* gene in this cluster in these snails is found elsewhere in the animal kingdom and is of any functional significance also remains a topic for future work. Overall, the PRD-class gene clustering provides a mixed signal, of both conservation of remnants of ancient clustering alongside rearrangements between closely related, con-generic species.

g) Duplicated homeobox genes

There are several duplications shared between the two species, which we infer to be at least ancestral to the genus. These include paralogues of *Arx*, *Pax4/6*, *Irx1*, *En*, *Evx*, *Abox*, *Barhl*, *Pbx*, and *Tlx*, as well as three paralogues of *Vsx* and *Cers*. Notably, the three paralogues each of *Vsx* and *Cers* genes remain clustered in the genome, reflecting their likely origin by tandem duplication. This is also seen for *En*, *Tlx*, *Evx*, and *Abox*. *B. straminea* is the only species of the two with two paralogues of *Gbx*, though one has an apparently odd arrangement that would mean it is unlikely to be a functional gene, if this arrangement were real. The homeodomain is split across two exons, the first of which is in one orientation, while there are two copies of the second exon in the opposite

orientation, indicating the second Gbx gene may be a pseudogene or an assembly artefact (Supplementary information S2a).

h) Giga-cluster homeobox genes

An overarching framework for understanding the genomic organization of homeobox-containing genes comes from hypotheses about their ancient linkage patterns following their presumed origins largely via tandem duplications. This ancestral clustering goes beyond the class-specific clusters already described above and is captured by the Giga-cluster hypothesis (Ferrier, 2016). High-quality genome assemblies, such as the one described here for *B. straminea,* are key resources for testing this hypothesis and potentially expanding it. Several instances of linkage of different classes of homeobox gene are present in the *B. straminea* assembly, most notably on scaffolds 563, 8789, 2216 and 24987 (Figure 2). Scaffold 2216 is interesting for the linkage of the SINE-class genes *Six4/5* and *Six1/2* with some of the members of the ancestral PRD/LIM-class Mega-cluster (i.e. the PRD-class genes *Gsc* and *Otx*) that has undergone some dispersal in the *Biomphalaria* lineage (as described above). Also, some of the other members of this dispersed PRD/LIM Mega-cluster (*Isl, Otp, Hbn*) are on scaffold 563, which are now linked with many members of the dispersed NK-cluster (e.g. *NK5, NK4, Msx, Tlx-a* and *-b,* and *NK3*) as well as a member of the ancestral SuperHox cluster (i.e. *Hhex*) (Butts et a., 2008; Ferrier, 2016). Other members of the SuperHox cluster are still linked with the true Hox genes (EuHox genes) on scaffold 32695. These linkages of genes from different homeobox classes along with the further new instances of inter-class linkage on scaffolds 8798 (Figure 2) are all consistent with the Giga-cluster hypothesis (Ferrier, 2016). However, how much of all of these linkages represent ancestral associations (i.e. descended from primary clustering) versus instances of coming together in the

genome convergently in evolution (i.e. secondary clustering) should be resolvable with comparisons to further high-quality genome sequences as well as a better understanding of the dynamics of genome evolution and rearrangements (reviewed in Ferrier, 2016).

Synteny analysis of B. straminea with other molluscs

The homeobox analyses described above provide instances of linkages that indicate varied synteny conservation across various mollusc and animal clades, even between the two *Biomphalaria* species now sequenced. The *B. straminea* genome shows considerable conserved linkage within and between classes of homeobox, and the maintenance of certain conserved clusters or linkages observed throughout wider lineages (i.e. instances of remnants of the Hox, ParaHox, SuperHox, and Giga-clusters (Ferrier, 2016)). In comparison to *B. glabrata*, in which less linkage can be observed because of shorter scaffold lengths, there is some conserved synteny. A few differences between the species may be due to species-specific genomic rearrangements resulting in the disruption of gene order, but the alternative possibility of assembly artefacts cannot be excluded entirely at present without further work. Of particular interest for further study is the major rearrangement of the Hox cluster in *B. straminea*. Perhaps more thorough sequencing of *B. glabrata* or assemblies of additional *Biomphalaria* species could determine if this is shared in the genus, or if it is a novelty of *B. straminea*. Regardless of this, the impact of this rearrangement on Hox gene expression and function is of interest. Hox cluster rearrangements could indicate the loss of shared regulatory elements that constrain Hox clusters in other lineages and may reflect changes to Hox gene expression, perhaps underpinning developmental changes in these snails. Similarly, the impacts of the dispersal of the ParaHox cluster on gene expression will be interesting to resolve.

The patterns of clustering, linkage, and rearrangement of homeobox genes are good markers for genome organization, and these results show that key differences between the species may represent higher levels of genomic divergence than expected for these two snails. Here we observe specific cases of differences between our new *B. straminea* genome and that of *B. glabrata* within the context of ancestral linkages, and this pattern may be a good indicator of wider differences between the genetics and molecular processes operating in the two species.

To examine the syntenic relationships more generally between *Biomphalaria* and mollusc genomes, we constructed Oxford dot-plots, comparing the chromosomal positions of orthologous genes between published mollusc genomes, as available from GenBank for gastropod, bivalve and cephalopod molluscs. As shown in Figure 4, the relationship of pseudo-chromosomes (2n=36, Adema et al 2017) and scaffolds between *B. straminea* and molluscs of other classes were conserved in most cases. Previous phylogenetic tree constructions for different *Biomphalaria* species suggested a monophyletic clade of African species with the remaining lineages being neotropical species (Campbell et al 2000; DeJong 2001). Based on this phylogenetic relationship, our data show that the neotropical species have not undergone any significant inter-chromosomal rearrangements from their last common ancestor after separation to different geographical regions. One-to-one synteny block could be identified between *B. straminea* and the eupulmonata gastropod, *Achatina immaculata*. However, in the comparison of *B. straminea* to the more evolutionary distant species, a few one-to-many blocks were found. These patterns indicated that some chromosome duplication and alteration occurred from the most recent common ancestor of *B. straminea*, *B. glabrata* and *A. immaculata* (the ancestor of Hygrophila and Eupulmonata). Further, species with closer evolutionary distance shared more similar synteny patterns against *B. straminea* (for example, between *Pomacea canaliculata* and *Marisa cornuarietis*, as well as

between *Crassostrea gigas* and *M. hongkongensis*, which they shared more similar synteny blocks), suggesting the dynamic changes of chromosomes arrangement in different molluscs. In *Octopus sinensis*, the gene order and synteny blocks to *B. straminea* were largely lost suggesting more duplication, translocation and rearrangement events occurred since the divergence of *O. sinensis* (Cephalopoda) and the common ancestor of Gastropoda and Bivalvia (Smith et al 2011).

Ecdysteroid genes

Ecdysteroids play important roles in regulating growth (in particular molting and metamorphosis) and sexual maturation of insects and other arthropods (Cheong et al 2015; Qu et al 2015). Although it has long been known that gastropods contain ecdysteroids, and that beta-ecdysone could stimulate host location activities in *S. mansoni* miracidia and enhance growth and egg production in *B. glabrata* (Bayne 1972; Shiff and Dossaji 1991), the biosynthetic pathway genes for ecdysteroids have not been systemically studied in mollusc genomes to date. As shown in Figure 3A-B, typical genes involved in this pathway including *CYP307A1*, *CYP306A1*, *CYP302A1*, *CYP315A1* and *CYP314A1* are all absent from the *B. straminea* genome assembly and transcriptome data. Nevertheless, the receptors including EcR, RXR/USP and oxygenase-like protein Nvd that are essential regulators of cholesterol metabolism are revealed in *B. straminea* and other mollusc genomes (Figure 3A-B; Supplementary information S3). We thus treated *B. straminea* with 10^{-6} M ecdysteroid 20-hydroxyecdysone for 24 hours but did not observe any significant expression changes in the downstream genes *E74*, *FOXO*, and *Nvd* . Similar hormone treatments have been shown to elicit the downstream genes in insects in previous studies (Hossain et al, 2013; Sekimoto et al 2007; Ji et al 2021). It is unclear whether only certain forms of ecdysteroids may induce endogenous ecdysteroid pathway genes under particular conditions and

this warrants further investigation. This is the first systematic analyses of ecdysteroid pathway genes in a mollusc genome, thus providing the foundations for future work to determine how ecdysteroids have their effect in these animals.

Insulin signaling pathway genes

Peptide hormones involved in growth and reproduction have been suggested as candidates for the development of novel methods of schistosomiasis control via manipulation of snail numbers (Acker et al 2019). Insulin is another understudied hormonal pathway in molluscs despite its potential functional roles. For instance, in the pond snail *Lymnaea stagnalis*, a decrease of insulin in the central nervous system correlated with better associative learning behaviour (Totani et al 2019), while insulin-related peptides with potential roles in sexual reproduction have been identified in the oyster *Crassostrea gigas* (Cherif-Feidel et al 2019). In both *B. straminea* and *B. glabrata* genomes, we were able to identify all key signalling pathway genes (Figure 3C-D, Supplementary S4). This establishes a foundation on which to further explore the functions of these hormones in molluscs.

Widespread gene turnover between Biomphalaria snails and other molluscs

a) Gene gains and losses in mollusc genomes

A phylogenomic tree was constructed using 2,047 orthogroups with at least 12 out of 13 mollusc genomes having single-copy genes in each orthogroup (Supplementary information 6). Gene family analysis among these genomes revealed the expansion of 1,869 orthogroups and

contraction of 623 orthogroups in *B. straminea*, while in *B. glabrata*, the expansion of 840 orthogroups and contraction of 1,035 orthogroups (Figure 5). This data highlights the importance of having the *B. straminea* genomic resource, and potentially suggested that specific control strategies might be needed for *B. straminea* rather than treating it as identical to *B. glabrata*.

b) Expansion of heat shock protein family among mollusc lineages

Heat shock proteins are important stress-responsive candidates involved in protein folding for molluscs, activated in response to such things as changing pH, oxygen level, and temperature. In some mollusc genomes, such as that of the Pacific oyster *Crassostrea gigas*, an expansion of heat shock protein 70 (HSP70) has been observed in the genome and hypothesized to be important to its adaptation to changes in ambient environmental factors or pressures (Zhang et al 2012). We thus identified the heat shock protein family genes in *Biomphalaria* and compared these to other lophotrochozoans to understand their evolution in different lineages (Figure 6). Among the different heat shock protein families in the investigated set of gastropods, bivalves, cephalopods, annelids, and platyhelminthes, a dramatic expansion is seen specifically in the HSP70 family in the bivalve molluscs (Figure 6; Supplementary information S7). Our data and analyses agree with previous studies (e.g. Zhang et al 2012), suggesting that the expansion of HSP70 is linked to the life history of molluscs having a sessile stage. This survey also provides the foundation for future work on the expression and function of particular HSP genes/proteins and their activity in these parasite vectors, which may contribute to their adaptive ability as invasive species, and possibly contributing to the recent range expansion of *B. straminea*.

c) Differential sesquiterpenoid and cholesterol genes in certain mollusc lineages

Sesquiterpenoid hormones were once considered specific to insects and crustaceans where they control development and reproduction (Cheong et al 2015; Qu et al 2018; Tsang et al 2020). However, recent analyses have shown that the sesquiterpenoid system is also present in myriapods, annelids, and cnidarians (Chipman et al 2014; Qu et al 2015; Schenk et al 2016; Nong et al 2020). Conversely, vertebrates can only produce cholesterol but not sesquiterpenoids (Tobe and Bendena 1999; Hui et al 2013), and a recent study revealed the canonical cholesterol biosynthesis pathway in sponges, placozoans and deuterostomes, suggesting cnidarians and protostomes experienced massive losses of these genes (Zhang et al 2019; Figure 7A). Treatment of 10^{-6} M simvastatin and methyl farnesoate on the snail *B. straminea* changed the expression of sesquiterpenoid pathway genes HMGCR and FPPS, suggesting a sesquiterpenoid responsive system (Figure 7B-C). Comparison of sesquiterpenoid pathway genes in mollusc genomes further identified differential utilization of biogenesis pathways in bivalves and gastropods, where only gastropods but not the bivalves are able to produce cholesterol similar to vertebrates (Figure 7D-F). This is the first systematic study showing the differential sesquiterpenoid and cholesterol pathways taken by different mollusc lineages.

Conclusion

This study presents the first high quality genome assembly for a schistosomiasis-transmitting snail in China and Asia. The snail *Biomphalaria straminea* is important scientifically as well as having considerable medical relevance. Our work provides gene and transposable element annotations, and detailed analyses of a variety of gene families, including the homeobox, ecdysteroid, insulin,

heat shock protein, and sesquiterpenoid pathway genes, suggesting extensive molecular differences between *B. straminea* and *B. glabrata* as well as among other molluscan taxa. More generally, our high-quality *B. straminea* genome provides a useful reference point for further understanding of the biology, ecology and evolution of molluscs.

Methods

Sample collection and genome sequencing

One week prior to the experiment, approximately 100 ramshore snails were collected in a freshwater stream in Tai Po New Territories, Hong Kong (GPS: 22.50206300747975, 114.15354682258841). The collected animals were kept and maintained in the laboratory

aquarium and fed with lettuce three days a week. Samples for genome sequencing originate from a single individual is used for preparing each sequencing method (Figure 1A). Genomic DNA (gDNA) was extracted using the PureLink Genomic DNA Mini Kit (Invitrogen) following the manufacturer's protocol. Extracted gDNA was subjected to quality control using a Nanodrop spectrophotometer (Thermo Scientific) and gel electrophoresis. Qualifying samples were sent to Novogene, and Dovetail Genomics for library preparation and sequencing. The resulting library was sequenced on an Illumina HiSeq X platform to produce 2 × 150 paired-end sequences. The length-weighted mean molecule length is 22.2 kb, and the raw data can be found at NCBI's Small Read Archive (SRR12963913).

Dovetail Omni-C library preparation and sequencing

For each Dovetail Omni-C library, chromatin was fixed with formaldehyde and extracted. Fixed chromatin was digested with DNAse I, and chromatin ends were repaired and ligated to a biotinylated bridge adapter followed by proximity ligation of adapter containing ends. After proximity ligation, crosslinks were reversed and the DNA was purified. Purified DNA was treated to remove biotin that was not internal to ligated fragments. Sequencing libraries were generated using NEBNext Ultra enzymes and Illumina-compatible adapters. Biotin-containing fragments were isolated using streptavidin beads before PCR enrichment of each library. The library was sequenced on an Illumina HiSeqX platform to produce 128 million 150 bp read pairs, and the raw data can be found at NCBI's Small Read Archive (SRR12963914).

Transcriptome sequencing

Total RNA from different tissues were isolated using a combination method of cetyltrimethylammonium bromide (CTAB) pre-treatment (Jordon-Thaden et. al. 2015) and mirVana™ miRNA Isolation Kit (Ambion) following the manufacturer's protocol. The extracted total RNA was subjected to quality control using a Nanodrop spectrophotometer (Thermo Scientific), gel electrophoresis, and an Agilent 2100 Bioanalyzer (Agilent RNA 6000 Nano Kit). Qualifying samples underwent library construction and sequencing at Novogene; polyA-selected RNA-Sequencing libraries were prepared using the TruSeq RNA Sample Prep Kit v2. Insert sizes and library concentrations of final libraries were determined using an Agilent 2100 bioanalyzer instrument (Agilent DNA 1000 Reagents) and real-time quantitative PCR (TaqMan Probe) respectively. Details of the sequencing data can be found in Supplementary information S1.

Genome assembly

Chromium WGS reads were used to construct a *de novo* assembly using Supernova (v 2.1.1) with default parameters (raw coverage = 68.32x). The Supernova output pseudohap assembly and Dovetail OmniC library reads were used as input data for HiRise, a software pipeline designed specifically for using proximity ligation data to scaffold genome assemblies (Putnam et al, 2016). Dovetail OmniC library sequences were aligned to the draft input assembly using bwa (https://github.com/lh3/bwa). The separations of Dovetail OmniC read pairs mapped within draft scaffolds were analyzed by HiRise to produce a likelihood model for genomic distance between read pairs, and the model was used to identify and break putative misjoins, to score prospective joins, and make joins above a threshold.

Gene model prediction

Gene models were predicted as described in the Hong Kong oyster (*Magallana hongkongensis*) genome (Li et al. 2020). Briefly, the gene models were trained and predicted using funannotate (v1.7.4,https://github.com/nextgenusfs/funannotate) (Palmer & Stajich, 2020) with the following parameters: "--repeats2evm --protein_evidence uniprot_sprot.fasta --genemark_mode ET --busco_seed_species metazoa --optimize_augustus --busco_db metazoa --organism other --max_intronlen 350000". The gene models from several prediction sources including GeneMark, high-quality Augustus predictions (HiQ), pasa, Augustus, GlimmerHM and snap were passed to Evidence Modeler and generated the gene model annotation files, followed by PASA to update the EVM consensus predictions, and add UTR annotations and models for alternatively spliced isoforms. Protein-coding genes were searched with BLASTp against the nr and swissprot databases by diamond (v0.9.24) (Buchfink et al., 2014) with parameters "--more-sensitive --evalue 1e-3", and mapped by HISAT2 (version 2.1.0) with transcriptome reads. Gene models with no similarity to any known protein and no mRNA support were removed from the final version.

Repetitive elements annotation

Repetitive elements were identified using the transposable element annotation pipeline earlGrey (Baril et al 2021) as follows. Firstly, elements were identified using RepeatMasker v.4.1 (Smit et al., 2013), using a sensitive (-s) search and ignoring low-complexity repeats (-nolow). Subsequently, a *de novo* repeat library was constructed using RepeatModeler v.1.0.11 (Smit et al., 2015), including RECON v.1.08 (Bao., et al 2002) and RepeatScout v.1.0.5 (Price et al., 2005).

Identified novel repeats were analysed using a 'BLAST, Extract, Extend' process to characterise elements along their entire length (Platt et al., 2016)[23]; Consensus sequences and classifications for each repeat family were generated, and the resulting *de novo* repeat library was utilised to identify repetitive elements in RepeatMasker. All plots were generated using Rstudio ver. 1.2.1335 with R ver. 3.5.1 (Team, 2013) and ggplot2 ver. 3.2.1 (Wickham, 2016).

Gene family annotation and gene tree building

Gene family sequences were first obtained from NCBI of neighboring species, including *B. glabrata* and other lophotrochozoans. The sequences were then used to retrieve the corresponding genes from the *B. straminea* genome using the tBLASTn algorithm on a local server, with an E-value smaller of 10^{-3}. The identity of each retrieved gene was then checked by reciprocal searches against the Genbank nr database at NCBI with BLASTx. For phylogenetic analyses of gene families, DNA sequences were first translated into amino-acid sequences and aligned to other reference sequences (extracted from NCBI) using Clustal W. Gapped sites were removed from alignments using MEGA 7.0, and phylogenetic trees (neighbor-joining) were constructed using MEGA 7.0, where each phylogenetic node was analysed using 1000 bootstrap replicates. For homeobox-containing genes, homeodomains were annotated using tBLASTn searches with HomeoDB sequences, and sequences from representative lophotrochozoan families, including the expanded Spiralia TALEs (Barton-Owen et al., 2018). We also removed redundant hits based on their unique locations in the genome sequence, and manually detected any likely artefactual duplicates which were not carried forward into the protein sequences alignments (Supplementary Table S2). Alignments of each class were made using MUSCLE (Edgar, 2004), with homeodomain sequences from human (*Homo sapiens*, deuterostome), amphioxus

(*Branchiostoma floridae*, a cephalochordate), the ecdysozoans fruitfly (*Drosophila melanogaster*), and red flour beetle (*Tribolium castaneum*), and the lophotrochozoans oyster (*Crassostrea gigas*, bivalve), limpet (*Lottia gigantea*, gastropod),brachiopod (*Lingula anatina*,), andannelids *Platynereis dumerilii* and *Capitella teleta*, where available from other studies (Paps et al., 2015; Barton-Owen et al., 2018) and HomeoDB (Ying-Fu et al., 2011; 2008). The best substitution models were tested with ModelFinder, and Maximum Likelihood phylogenies were constructed with IQ-TREE with 1000 bootstrap replicates (Nguyen et al., 2015).

Identification of orthologous genes and gene families

Orthologues and orthogroups in *B. straminea* and 12 other animal proteomes were inferred using OrthoFinder v. 2.5.2 [28] with default values and '-M msa' activated. To cover the gene families, the longest protein of each gene was taken as the representative in OrthoFinder analysis. Gene duplication events were then identified. Duplication ratios per node/tip were calculated by dividing the number of duplications observed in each node/tip by the total number of gene trees containing that node. CAFE5 was used to infer gene gain and loss rates [29]. Orthogroups from output of OrthoFinder were regarded as gene families and fed to CAFE5. A divergence tree was inferred using r8s [30] from the species tree generated by OrthoFinder. We tested several gamma rate categories (-k) and k=1 showed the best likelihood.

Functional terms enrichment analysis

Orthogroups were assigned Gene Ontology (GO), EuKaryotic Orthologous Groups (KOG), Kyoto Encyclopedia of Genes and Genomes (KEGG), and KEGG Orthology (KO) terms

by inheriting the terms from genes found within the groups. The functional term annotations were performed using eggNOG [31]. Functional enrichment was tested for using function ‘compareCluster()’ in R package ‘clusterProfiler’ v.3.16.1 [32] under the environment of R 4.0.4 [33]. Significantly enriched terms were determined with pvalueCutoff = 0.05, pAdjustMethod = "BH", and qvalueCutoff = 0.2. Data was visualised using R packages ‘ggplot2’ [25], ‘ggtree’ [34] and ‘pathview’ [35].

Macrosynteny analysis

Single-copy orthologues anchored by mutual best Diamond blastp v0.9.14.115[17] hits (--evalue 0.001) between *B. straminea* and 12 other animals with chromosome-level or near chromosome-level assemblies were used in macrosynteny analysis. Oxford synteny plots were generated following previously described methods [36] using R packages ‘ggplot2’ [25].

Drug and hormone treatment and RT-qPCR

Experimental adult animals of ~1cm with reproductive capability were isolated from the culture and were rinsed in double-distilled water to remove any contaminants. Three individuals per set were placed in a glass container, with a well of 3.5cm in radius and 0.8cm in depth, filled with 2ml of double-distilled water with either 10^{-6}M or 10^{-8}M of methyl farnesoate (MF) (Sigma), $6x10^{-5}$M of simvastatin (Sigma) or 10^{-6}M of 20-hydroxyecdysone (AbcamBiochemicals) in separate setups. The chemicals were first dissolved in acetone and diluted to the target concentration in the treatment container. The control setup contained the same number of

individuals and was treated with the same concentration of acetone in corresponding experiments. Each replicate of snails was exposed for 24 hours to these treatments without any feeding. Post-treated animals were rinsed with double-distilled water and shells were removed for whole body total RNA extraction. The RNA from each experiment was isolated using TRIzol reagent following the manufacturer's protocol. Purified RNA was dissolved in nuclease-free water. The cDNA synthesis was performed using the iScript gDNA Clear cDNA Synthesis Kit (BioRad) following the manufacturer's protocol. The cDNA was used in subsequent quantitative real time PCR. The amplification conditions were as follows: initial denaturation at 95 °C for 30 s, followed by 40 cycles of 95 °C denaturation for 15s, 57 °C primer annealing for 15s and 72°C extension for 15s. Primer details are listed in Supplementary File S8. The primers were tested by conventional PCR with *B. straminea* cDNA prior to experiments to ensure its specificity. Each sample was analyzed in replicates. The expression of each target gene transcript was normalized to the housekeeping gene, myoglobin (Myo), as adopted in previous studies (Jiang et al 2006; Arican-Goktas et al 2014; Queiroz et al 2017; Pinaud et al 2021). The subsequent fold induction analyses were calculated using the ΔΔCt method.

Ethics Statement

N/A

Availability of Supporting Data and Materials

The raw genome and RNA sequencing data have been deposited in the SRA under Bioproject number PRJNA673593. The final chromosome assembly was submitted to NCBI Assembly under accession number JADKLZ000000000 in NCBI. All data is available from the corresponding author upon reasonable request.

Figure legends

Figure 1. A) Life cycle of snail *Biomphalaria straminea*; B) Comparison of snail *Biomphalaria* genome assembly quality; C) Transposable elements in *Biomphalaria straminea*.

Figure 2. Distribution of Homeoboxes in the genome of *Biomphalaria straminea.* Class is denoted by colour, arrows show orientation on each scaffold, which are represented by black lines and are numbered underneath. *Post2* is not found in the genomic sequence but is found in the transcriptome, so is not shown on a scaffold. Grey gene names and box outlines denote partial homeodomain sequences.

Figure 3. A) Schematic diagram of biosynthetic pathway of ecdysteroids; B) Presence and absence of ecdysteroid pathway genes in *B. straminea*; C) Expression of genes upon 10^{-6}M 20-hydroxyecdysone treatment for 24 hours (n=13-15); D) Schematic diagram of biosynthetic pathway of insulin; E) Number of gene copies of insulin pathway genes in *B. straminea*.

Figure 4. Synteny between *B. straminea* and other 12 mollusc genomes. The species tree is constructed using 2,047 orthogroups with at least 12 out of 13 mollusc genomes having single-copy genes in each orthogroup. In the Oxford dot plot, each dot represents a pair of orthologous

genes between *B. straminea* and the specific mollusc. Horizontal and vertical dashed lines represent chromosome or scaffold boundaries. Orthologous genes are colored according to their position in *B. straminea* scaffolds. Significance of synteny blocks is computed using one-tailed Fisher's exact test, and the color of synteny blocks with Benjamini & Hochberg corrected p over 0.05 are turned into grey.

Figure 5. Summaries of gene families in *B. straminea* and other 12 mollusc. A) Gene family clustering, only the longest isoform for each gene was used; B) Gene family expansion and contraction between mollusc genomes. Brown and green color indicate the number of significantly ($p<0.05$) expanded or contracted gene families at each node, respectively.

Figure 6. A) Schematic diagram showing the heat shock proteins actions; B) Number of gene copies of heat shock proteins in different mollusc genomes. The purple box highlights the expansion of HSP70 in certain mollusc lineages.

Figure 7. A) Schematic diagram showing the mevalonate pathway, and the downstream sesquiterpenoid and *de novo* cholesterol synthesis pathways. B) Expression of genes upon $6x10^{-5}$M simvastatin, 10^{-6}M and 10^{-8}M methyl farnesoate treatment for 24 hours; *= $p < 0.05$. C) Heatmap of mevalonate pathway orthologues identified in gastropod and bivalve genomes. D) Heatmap of sesquiterpenoid synthesis pathway orthologues identified in gastropod and bivalve genomes. E) Heatmap of *de novo* cholesterol synthesis pathway orthologues identified in gastropod and bivalve genomes. F) Schematic diagram showing the evolution of sesquiterpenoid pathway genes in bilaterians.

Additional Files.

Supplementary information S1. Sequencing data.

Supplementary information S2. a) Tables of homeobox genes sequences in *B. straminea*, *B. glabrata*, a synteny comparison of homeobox genes, and comparison of ParaHox gene linkage. b) Distribution of Homeoboxes in the genome of *Biomphalaria glabrata*. c) Alignments and phylogenies of each class of Homeobox sequences.

Supplementary information S3. Ecdysteroid genes.

Supplementary information S4. Insulin pathway genes.

Supplementary information S5. Synteny information

Supplementary information S6. Gene expansion and contraction.

Supplementary information S7. Heat shock protein family genes.

Supplementary information S8. Cholesterol genes and primers.

Supplementary information S9. Phylogenetic trees.

Supplementary information S10. Tables.

Abbreviations.

BLAST: Basic Local Alignment Search Tool; BUSCO: Benchmarking Universal Single-Copy Orthologs; kb: kilobase pairs; Mb: megabase pairs; NCBI: National Center for Biotechnology Information; TE: transposable element

Competing Interests

The authors declare that they have no competing interests.

Funding

This work was supported by the Hong Kong Research Grant Council Collaborative Research Fund (C4015-20EF), General Research Fund (14100919), NSFC/RGC Joint Research Scheme (N_CUHK401/21), and The Chinese University of Hong Kong Direct Grant (4053433, 4053489). YY, WLS, CFW, STSL, and YL were supported by the PhD studentships of The Chinese University of Hong Kong. AH is supported by a Biotechnology and Biological Sciences Research Council (BBSRC) David Phillips Fellowship (BB/N020146/1). TB is supported by a studentship from the Biotechnology and Biological Sciences Research Council-funded South West Biosciences Doctoral Training Partnership (BB/M009122/1). MEAR is supported by a PhD studentship from the School of Biology and St Andrews University.

Authors' Contributions

JHLH, DEKF, AH, ZW, SX, ZPK, SSC conceived the study. JHLH, DEKF, AH supervised the study. WN, JH, TS assembled the genome. WN carried out the gene model prediction and comparison. YY carried out the heat shock proteins analyses. YX carried out the gene gain and loss and synteny analyses. WLS and CFW carried out the sesquiterpenoid analyses. YY, WLS and SYL carried out the ecdysteroid analyses. MEAR and YL carried out the homeobox gene analyses. TB carried out the transposable element analyses. STSL carried out the insulin analyses. WN, YY, YX, WLS, MEAR, TB, AH, DEKF, JHLH wrote the first draft of manuscript. All authors approved the final version of the manuscript.

Acknowledgements

The authors would like to thank Elaine Huang and Ho Yin Yip for collection and maintenance of snails. We thank Thomas Barton-Owen for help and advice on homeobox searches. The authors would also like to thank comments from the anonymous reviewers in improving the manuscript.

References

Acker MJ, Habib MR, Beach GA, et al.. An immunohistochemical analysis of peptidergic neurons apparently associated with reproduction and growth in *Biomphalaria alexandrina*. *Gen Comp Endocrinol*. 2019;280:1-8.

Adema CM, Hillier LDW, Jones CS, et al.. Whole genome analysis of a schistosomiasis-transmitting freshwater snail. *Nat Commun*; 2017;8:1-12.

Altschul SF, Gish W, Miller W, et al.. Basic local alignment search tool. *J Mol Biol*. 1990;215:403-410.

Arican-Goktas HD, Ittiprasert W, Bridger JM. et al.. Differential spatial repositioning of activated genes in *Biomphalaria glabrata* snails infected with *Schistosoma mansoni*. *PLoS neglected tropical diseases*. 2014;8: e3013.

Attwood SW, Huo GN & Qiu JW. Update on the distribution and phylogenetics of *Biomphalaria* (Gastropoda: Planorbidae) populations in Guangdong Province, China. *Acta tropica*. 2015;141: 258-270.

Bao Z & Eddy SR. Automated *De Novo* Identification of Repeat Sequence Families in Sequenced Genomes. *Genome research*. 2002;12:1269-1276.

Baril T, Imrie R & Hayward A. Earl Grey. Zenodo. 2021.

Barton-Owen TB., Szabó R, Somorjai IML, et al.. A revised spiralian homeobox gene classification incorporating new polychaete transcriptomes reveals a diverse TALE class and a divergent hox gene. *Genome Biol. Evol*. 2018;10:2151-2167.

Bayne CJ. On the reported occurrence of an ecdysone-like steroid in the freshwater snail, *Biomphalaria glabrata* (Pulmonata; Basommatophora), intermediate host of *Schistosoma mansoni*. *Parasitology*. 1972;64:501-509.

Buchfink B, Xie C & Huson DH. Fast and sensitive protein alignment using DIAMOND. Nat Methods 2014 121. *Nature methods*. 2015;12:59-60.

Butts T, Holland PWH & Ferrier DEK. The Urbilaterian Super-Hox cluster. *Trends Genet*. 2008;24:259-262.

Campbell G, Jones CS, Lockyer AE, et al.. Molecular evidence supports an African affinity of the Neotropical freshwater gastropod, *Biomphalaria glabrata*, Say 1818, an intermediate host for *Schistosoma mansoni*. *Proc R Soc B Biol Sci. Royal Society*. 2000;267:2351-2358.

Castro LFC & Holland PWH. Chromosomal mapping of ANTP class homeobox genes in amphioxus: piecing together ancestral genomes. *Evol. Dev*. 2003;5:459–465.

Cheong SPS, Huang J, Bendena WG, et al.. Evolution of ecdysis and metamorphosis in arthropods: The rise of regulation of juvenile hormone. *Integr Comp Biol*. 2015;55: 878-890.

Cherif—Feildel M, Heude Berthelin C, Adeline B, et al.. Molecular evolution and functional characterisation of insulin related peptides in molluscs: Contributions of *Crassostrea gigas* genomic and transcriptomic-wide screening. Gen Comp Endocrinol. 2019;271:15-29.

Chipman AD, Ferrier DEK, Brena C, et al.. The First Myriapod Genome Sequence Reveals Conservative Arthropod Gene Content and Genome Organisation in the Centipede *Strigamia maritima*. *PLoS Biol*. 2014;12:e1002005.

Chourrout D, Delsuc F, Chourrout P, et al.. Minimal ProtoHox cluster inferred from bilaterian and cnidarian Hox complements. *Nature*. 2006;442:684-687.

DeJong RJ, Morgan JA, Paraense WL, et al.. Evolutionary relationships and biogeography of *Biomphalaria* (Gastropoda: Planorbidae) with implications regarding its role as host of the human bloodfluke, *Schistosoma mansoni*. *Mol. Biol. Evol.*. 2001;18:2225-2239.

Dudgeon D & Yipp MW. A report on the gastropod fauna of aquarium fish farms in Hong Kong, with special reference to an introduced human schistosome host species, *Biomphalaria straminea* (Pulmonata: Planorbidae). *Malacol Rev*. 1983;16:93-94.

Edgar RC. MUSCLE: Multiple sequence alignment with high accuracy and high throughput. *Nucleic Acids Res*. 2004;32:1792-1797.

Emms DM & Kelly S. OrthoFinder: Phylogenetic orthology inference for comparative genomics. *Genome Biol*. 2019;20:1-14.

Ferrier DEK. Evolution of Homeobox Gene Clusters in Animals: The Giga-Cluster and Primary vs. Secondary Clustering. *Front. Ecol. Evol*. 2016;4:1-13.

Hossain MS, Liu Y, Zhou S, et al.. 20-Hydroxyecdysone-induced transcriptional activity of FoxO upregulates brummer and acid lipase-1 and promotes lipolysis in *Bombyx* fat body. *Insect biochemistry and molecular biology*. 2013;43:829-838.

Huerta-Cepas J, Szklarczyk D, Heller D, et al.. eggNOG 5.0: a hierarchical, functionally and phylogenetically annotated orthology resource based on 5090 organisms and 2502 viruses. *Nucleic acids research*. 2019;47:309-314.

Hui JHL, Bendena WG & Tobe SS. Future perspectives for research on the biosynthesis of juvenile hormones and related sesquiterpenoids in arthropod endocrinology and ecotoxicology. Juv Horm Juvenoids Model Biol Eff Environ Fate. CRC Press, Boca Raton, 15-30, 2013.

Hui JHL, McDougall C, Monteiro AS, et al.. Extensive chordate and annelid macrosynteny reveals ancestral homeobox gene organization. Mol. Biol. Evol. 2012;29:157-165.

Ikuta T, Chen YC, Annunziata R, et al.. Identification of an intact ParaHox cluster with temporal colinearity but altered spatial colinearity in the hemichordate *Ptychodera flava*. *BMC Evol. Biol.*, 2013;13:1-10.

Irimia M, Maeso I, & Garcia-Fernandez J. Convergent Evolution of Clustering of Iroquois Homeobox Genes across Metazoans. *Mol. Biol. Evol*. 2008;25:1521-1525.

Ji C, Zhang N, Jiang H, et al.. 20-hydroxyecdysone regulates expression of methioninesulfoxide reductases through transcription factor FOXO in the red flour beetle, *Tribolium castaneum*. *Insect Biochemistry and Molecular Biology*, 2021;131:103546.

Jiang Y, Loker ES & Zhang SM. *In vivo* and *in vitro* knockdown of FREP2 gene expression in the snail *Biomphalaria glabrata* using RNA interference. *Developmental & Comparative Immunology*. 2006;30: 855-866.

Jordon-Thaden IE, Chanderbali AS, Gitzendanner MA, et al.. Modified CTAB and TRIzol Protocols Improve RNA Extraction from Chemically Complex Embryophyta. Appl Plant Sci. 2015;3.

Kerner P, Ikmi A, Coen D, et al.. Evolutionary history of the iroquois/Irx genes in metazoans. *BMC Evol. Biol*. 2009;9:1–14.

Kim D, Paggi JM, Park C, et al.. Graph-based genome alignment and genotyping with HISAT2 and HISAT-genotype. Nature biotechnology. 2019;37:907-915.

Kumar S, Stecher G & Tamura K. MEGA7: Molecular Evolutionary Genetics Analysis Version 7.0 for Bigger Datasets. *Mol Biol Evol*. 2016;33: 1870-1874.

Li H & Durbin R. Fast and accurate short read alignment with Burrows-Wheeler transform. *Bioinformatics*. 2009,25:1754-1760.

Li Y, Nong W, Baril T, et al.. Reconstruction of ancient homeobox gene linkages inferred from a new high-quality assembly of the Hong Kong oyster (*Magallana hongkongensis*) genome. *BMC Genomics*. 2020;21:1-17.

Luo W & Brouwer C. Pathview: An R/Bioconductor package for pathway-based data integration and visualization. *Bioinformatics*. 2013;29:1830-1831.

Mazza ME, Pang K, Reitzel AM, et al.. A conserved cluster of three PRD-class homeobox genes (homeobrain, rx and orthopedia) in the Cnidaria and Protostomia. *Evodevo*. 2010;1:3.

Meier-Brook C. A snail intermediate host of *Schistosoma mansoni* introduced into Hong Kong. *Bull World Health Organ*. 1974;51:661.

Mendes FK, Vanderpool D, Fulton B, e al.. CAFE 5 models variation in evolutionary rates among gene families. Robinson P, editor. Bioinformatics. Oxford University Press (OUP); 2020.

Nguyen L-T, Schmidt HA, von Haeseler A, et al.. IQ-TREE: A Fast and Effective Stochastic Algorithm for Estimating Maximum-Likelihood Phylogenies. *Mol. Biol. Evol*. 2015;32:268-274.

Nong W, Cao J, Li Y, et al.. Jellyfish genomes reveal distinct homeobox gene clusters and conservation of small RNA processing. *Nat Commun*. 2020;11:1-11.

Osborne PW, Benoit G, Laudet V, et al.. Differential regulation of ParaHox genes by retinoic acid in the invertebrate chordate amphioxus (*Branchiostoma floridae*). Developmental Biology. 2009;327:252-262.

Palmer JM & Stajich J. nextgenusfs/funannotate: funannotate v1.7.4 (Version 1.7.4). Zenodo.

Paps J, Xu F, Zhang G, et al.. Reinforcing the egg-timer: Recruitment of novel Lophotrochozoa homeobox genes to early and late development in the Pacific oyster. Genome Biol. Evol. 2015;7:677-688.

Pinaud S, Tetreau G, Poteaux P, et al.. New insights into biomphalysin gene family diversification in the vector snail *Biomphalaria glabrata*. *Frontiers in immunology*. 2021;12:918.

Platt RN, Blanco-Berdugo L & Ray DA. Accurate transposable element annotation is vital when analyzing new genome assemblies. *Genome Biol Evol*. 2016;8:403-410.

Price AL, Jones NC & Pevzner PA. *De novo* identification of repeat families in large genomes. *Bioinformatics*. 2005;21:351-358.

Putnam NH, Connell BO, Stites JC, et al.. Chromosome-scale shotgun assembly using an *in vitro* method for long-range linkage. *Genome research*. 2016;26:342-350.

Qu Z, Bendena WG, Tobe SS, et al.. Juvenile hormone and sesquiterpenoids in arthropods: Biosynthesis, signaling, and role of MicroRNA. *J. Steroid Biochem. Mol. Biol*. 2018;184:69-76.

Qu Z, Kenny NJ, Lam HM, et al.. How Did Arthropod Sesquiterpenoids and Ecdysteroids Arise? Comparison of Hormonal Pathway Genes in Noninsect Arthropod Genomes. *Genome Biol Evol*. 2015;7:1951-1959.

Queiroz FR, Silva LM, Jeremias WDJ, et al.. Differential expression of small RNA pathway genes associated with the *Biomphalaria glabrata*/*Schistosoma mansoni* interaction. *PLoS one*. 2017;12: e0181483

R Core Team. R: a language and environment for statistical computing. Vienna, Austria; 2013.

Sanderson MJ. r8s: Inferring absolute rates of molecular evolution and divergence times in the absence of a molecular clock. Bioinformatics. Oxford Academic; 2003.

Schenk S, Krauditsch C, Frühauf P, et al.. Discovery of methylfarnesoate as the annelid brain hormone reveals an ancient role of sesquiterpenoids in reproduction. *Elife*. 2016;5:e17126.

Sekimoto T, Iwami M & Sakurai S. 20- Hydroxyecdysone regulation of two isoforms of the Ets transcription factor E74 gene in programmed cell death in the silkworm anterior silk gland. *Insect molecular biology*. 2007;16:581-590.

Shiff CJ & Dossaji SF. Ecdysteroids as regulators of host and parasite interactions: a study of interrelationships between *Schistosoma mansoni* and the host snail, *Biomphalaria glabrata*. *Trop Med Parasitol*. 1991;42:11-6.

Simakov O, Marlétaz F, Cho S-J, et al.. Insights into bilaterian evolution from three spiralian genomes. *Nature*. 2013;493:526-531.

Simakov O, Marlétaz F, Yue JX, et al.. Deeply conserved synteny resolves early events in vertebrate evolution. *Nat Ecol Evol*. 2020;4:820-830.

Simão FA, Waterhouse RM, Ioannidis P, et al.. BUSCO: Assessing genome assembly and annotation completeness with single-copy orthologs. *Bioinformatics*. 2015;31:3210-3212.

Smit A & Hubley R. RepeatModeler Open-1.0. http://repeatmasker.org. 2015.

Smit AFA, Hubley RR & Green PR. RepeatMasker Open-4.0. http://repeatmasker.org. 2015.

Smith SA, Wilson NG, Goetz FE, et al.. Resolving the evolutionary relationships of molluscs with phylogenomic tools. *Nature*. 2011;480:364-367.

Takatori N, Butts T, Candiani S, et al.. Comprehensive survey and classification of homeobox genes in the genome of amphioxus, *Branchiostoma floridae*. *Dev. Genes Evol*. 2008;218:579-590.

Team RC. R: A language and environment for statistical computing. Vienna, Austria; 2013.

Tennessen JA, Bollmann SR, Peremyslova E, et al.. Clusters of polymorphic transmembrane genes control resistance to schistosomes in snail vectors. *Elife*. 2020.

Tobe SS & Bendena WG. The regulation of juvenile hormone production in arthropods. Functional and evolutionary perspectives. *Ann N Y Acad Sci*. 1999;897:300-310.

Totani Y, Aonuma H, Oike A, et al.. Monoamines, insulin and the roles they play in associative learning in pond snails. *Front. Behav. Neurosci*. 2019;13:65.

Tsang SSK, Law STS, Li C, et al.. Diversity of Insect Sesquiterpenoid Regulation. *Front. Genet*. 2020;11:1027.

Wang L, Wu X, Li X, et al.. Imported Schistosomiasis: A New Public Health Challenge for China. *Front Med*. 2020;7.

Wickham H. ggplot2: Elegant graphics for data analysis. New York: Springer-Verlag. 2019.

Woodruff DS, Mulvey M & Yipp MW. The continued introduction of intermediate host snails to *Schistosoma mansoni* into Hong Kong. Bull World Heal Organ. 1985;63:621.

Yang Y, Cheng W, Wu X, et al.. Prediction of the potential global distribution for *Biomphalaria straminea*, an intermediate host for *Schistosoma mansoni*. *PLOS Negl Trop Dis*. 2018;12:e0006548

Yu G, Smith DK, Zhu H, et al.. ggtree: an r package for visualization and annotation of phylogenetic trees with their covariates and other associated data. *Methods Ecol Evol*. 2017;8:28-36.

Yu G, Wang L-G, Han Y, et al.. clusterProfiler: an R package for comparing biological themes among gene clusters. *Omics: a journal of integrative biology*. 2012;16:284-287.

Zeng X, Yiu WC, Cheung KH, et al.. Distribution and current infection status of *Biomphalaria straminea* in Hong Kong. 2017;10:1-12

Zhang G, Fang X, Guo X, et al.. The oyster genome reveals stress adaptation and complexity of shell formation. *Nature*. 2012;490:49-54.

Zhang H, Ravi V, Tay BH, et al.. Lampreys, the jawless vertebrates, contain only two ParaHox gene clusters. *PNAS*. 2017;114: 9146-9151.

Zhang T, Yuan D, Xie J, et al.. Evolution of the Cholesterol Biosynthesis Pathway in Animals. *Mol Biol Evol*. 2019;36:2548-2556.

Zhong Y & Holland PWH. HomeoDB2: functional expansion of a comparative homeobox gene database for evolutionary developmental biology. *Evol. Dev*. 2011;13:567–568.

Zhong Y-F, Butts T, & Holland PWH. HomeoDB: a database of homeobox gene diversity. *Evol. Dev*. 2008;10:516-518.

Zhu R & Xu J. Epidemic situation of oversea imported schistosomiasis in China and thinking about its prevention and control. *Chinese journal of schistosomiasis control*. 2014;26:111-114.

Figure 1

Click here to access/download;Figure;Fig1.TIF

A)

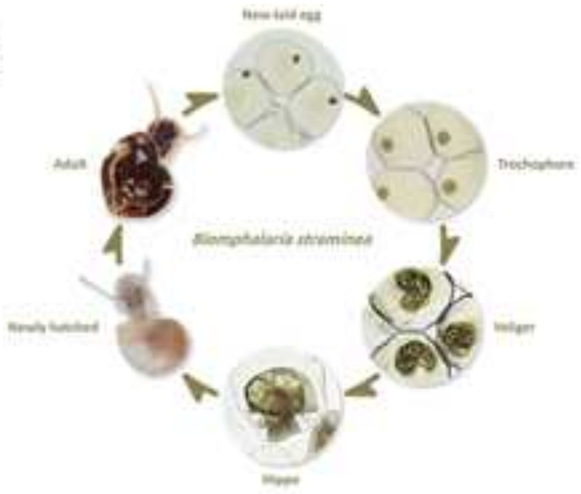

B)

	B. straminea	*B. glabrata*	*B. glabrata*		
Accession number	JADKLZ000000000	GCF_000457365	GCA_014525025	GCA_014524965	GCA_014524955
Assembly size (bp)	1,004,745,081	1,004,569,788	810,878,449	852,019,028	767,739,590
Scaffold N50 (bp)	25,272,813	37,441	743,154	2,598,223	394,791
Number of scaffolds	84,585	373,617	2,718	927	3,492
Gaps	4.96%	1.74%	0	0	0
BUSCO (metazoa_odb10)	87.00%	89.90%	92.50%	93.70%	88.50%
Gene models	43,340	30,855	N.A.	N.A.	N.A.
References	This study	Adema et al 2017	Tennessen et al 2020		

C)

Repeat Class	*Biomphalaria straminea*		
	No. of elements	Total Length (Mb)	Percentage sequence
**Retroelement**	315626	146.15	14.55%
SINE	45672	13.17	1.31%
LINE	241927	105.30	10.48%
Penelope	1386	0.57	0.06%
LTR element	26641	27.11	2.70%
**DNA transposon**	179893	83.56	8.32%
**Rolling-circle**	34786	17.20	1.71%
**Unclassified**	359515	158.89	15.81%
**Other**	13502	2.95	0.29%
**Total repeats**	903322	408.75	40.68%

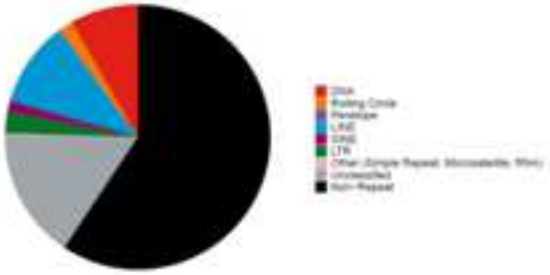

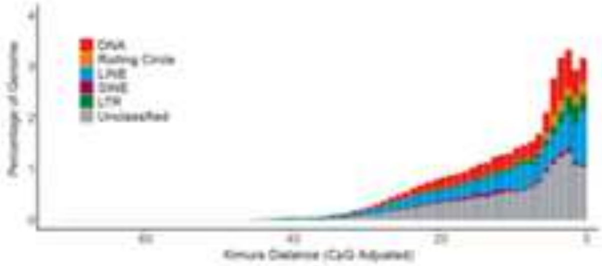

Fig 1

Figure 2 Click here to access/download;Figure;Fig2.TIF

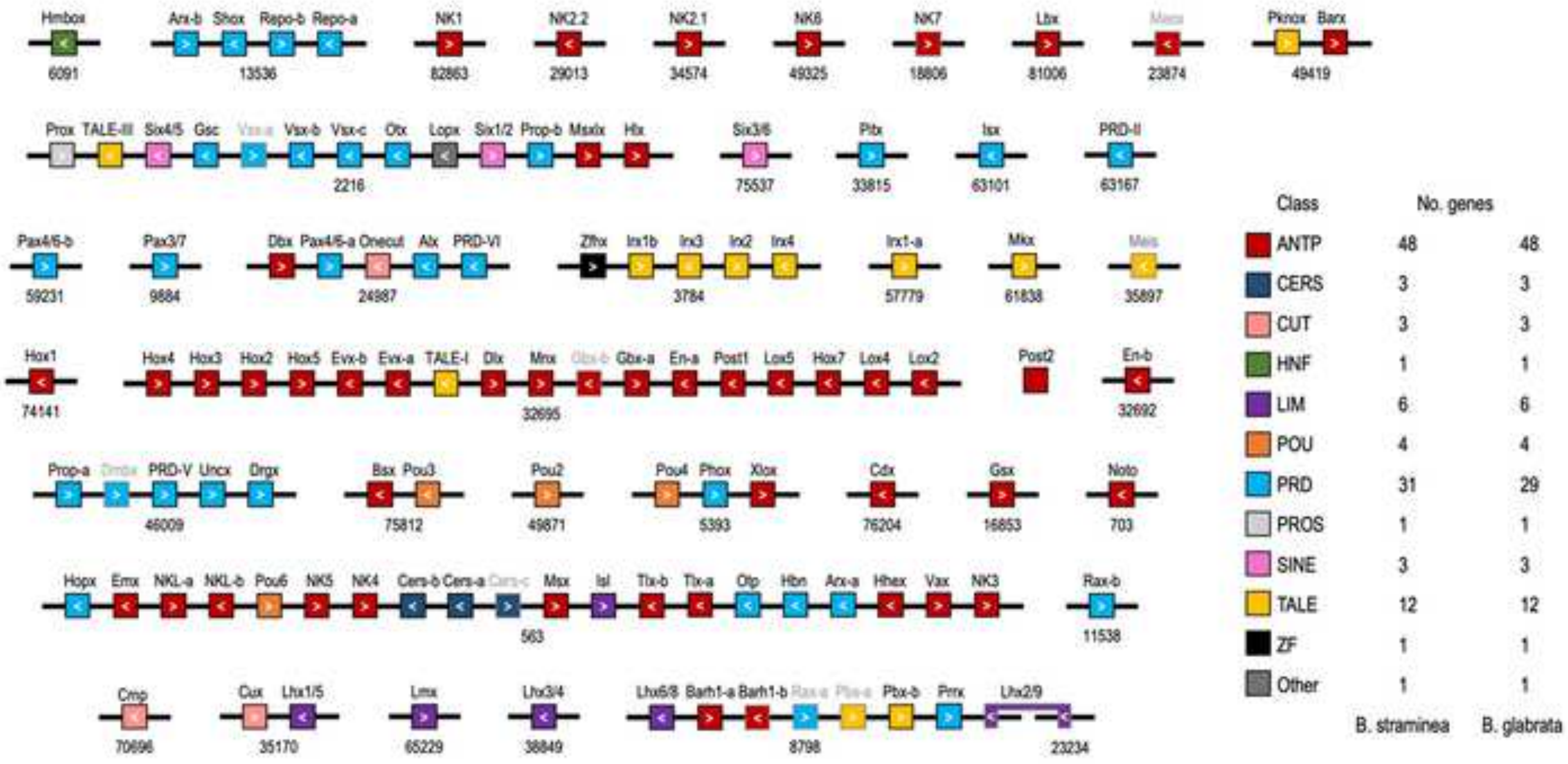

Fig 2

Figure 3

Click here to access/download;Figure;Fig3.TIF

A)

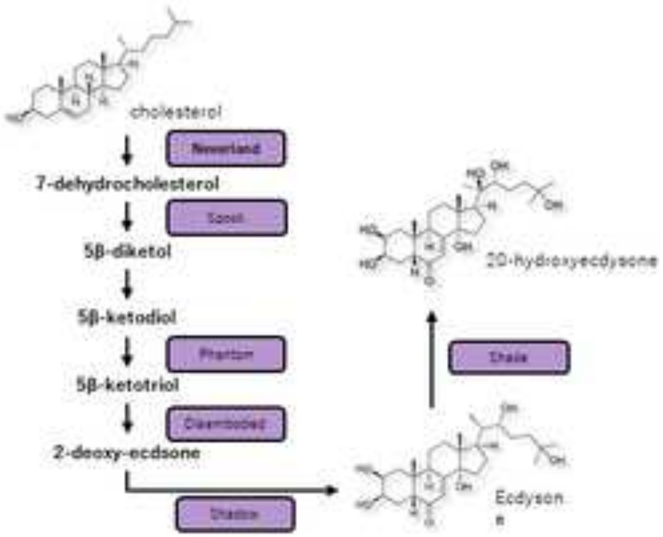

B)

Gene name	Abbr.	Function	*B. straminea*
cholesterol 7-desaturase (Neverland)	*Nvd*	convert cholesterol to 7-Dehydro-cholesterol	+
spook (CYP307A1)	*spo*	Synthesize 20-hydroxyecdysone (20E) from cholesterol	+
phantom (CYP306A1)	*phm*	Synthesize 20E from cholesterol	+
disembodied (CYP302A1)	*dib*	Synthesize 20E from cholesterol	-
shadow (CYP315A1)	*sad*	Synthesize 20E from cholesterol	-
shade (CYP314A1)	*shd*	Synthesize 20E from cholesterol	-
CYP18A1	/	Paralog of CYP306	+
Canonical regulatory components as shown in other arthropods			
Ecdysone receptor	*EcR*	Ecdysteroids regulate molting by activating a heterodimer formed by the ecdysone receptor (EcR) and retinoid X receptor (RXR)	+
Retinoid X receptor/ultraspiracle	*RXR/USP*	Ecdysteroids regulate molting by activating a heterodimer formed by the ecdysone receptor (EcR) and retinoid X receptor (RXR)	+

C)

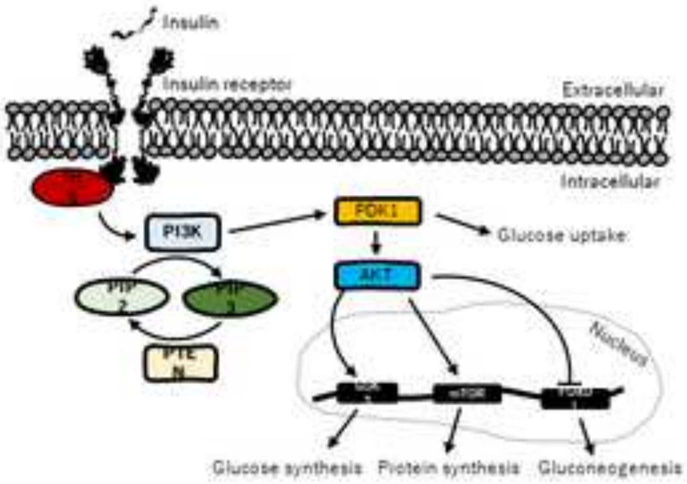

D)

Gene name	Abbr.	*B. straminea*	*B. glabrata*
insulin	INS	7	8
insulin receptor	INSR	2	2
insulin receptor substrate 1	IRS1	1	1
phosphatidylinositol-4,5-bisphosphate 3-kinase catalytic subunit alpha/beta/delta	PI3K	1	1
3-phosphoinositide dependent protein kinase-1	PDPK1	1	1
atypical protein kinase C zeta type	aPKC	1	1
RAC serine/threonine-protein kinase	AKT	1	1
forkhead box protein O1	FOXO1	1	1
serine/threonine-protein kinase mTOR	mTOR	1	1
glycogen synthase kinase 3 beta	GSK3B	1	1
phosphatidylinositol-3,4,5-trisphosphate 3-phosphatase and dual-specificity protein phosphatase PTEN	PTEN	1	1

Fig 3

Click here to access/download;Figure;Fig4.TIF

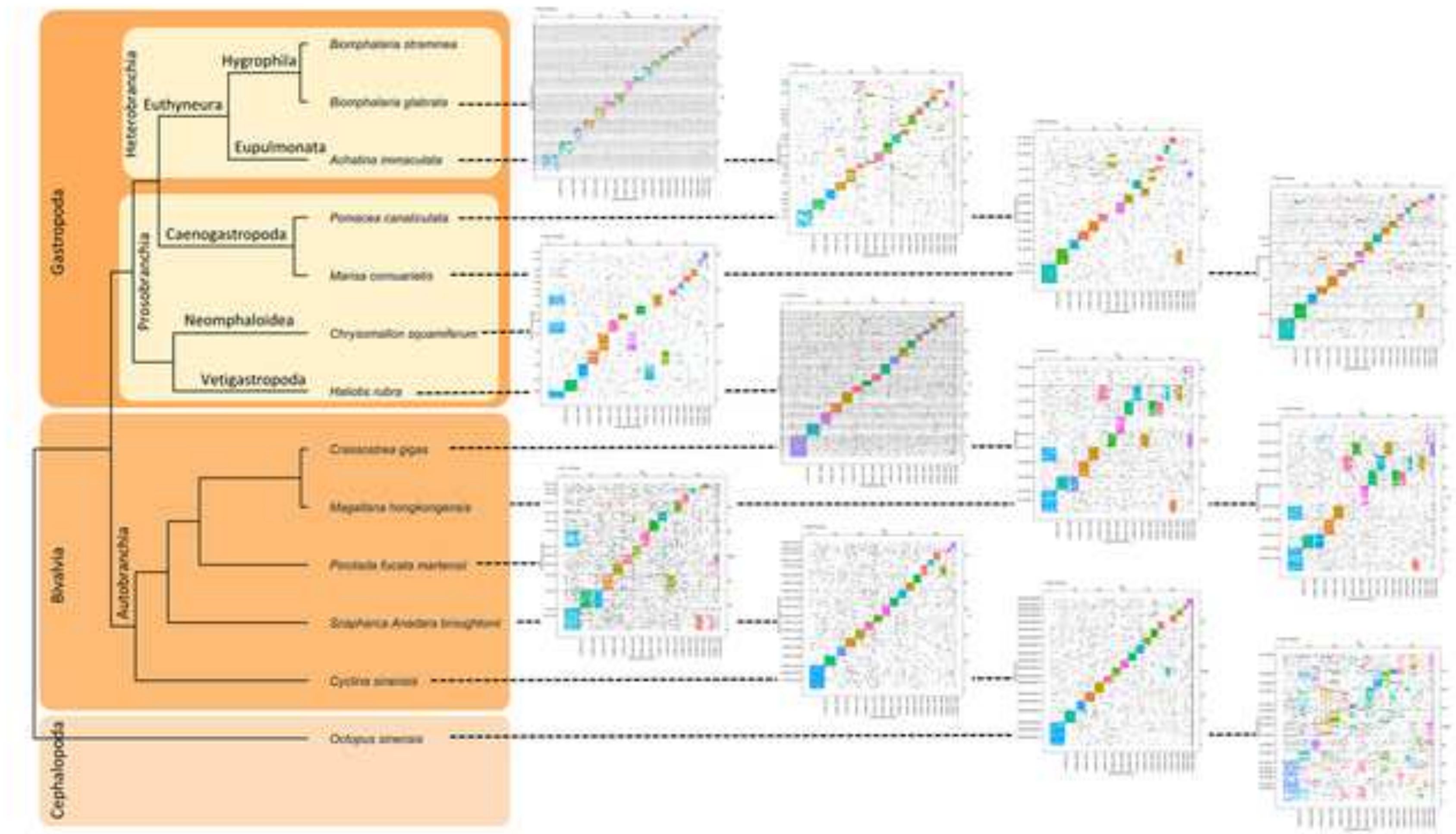

Fig 4

Click here to access/download;Figure;Fig5.TIF

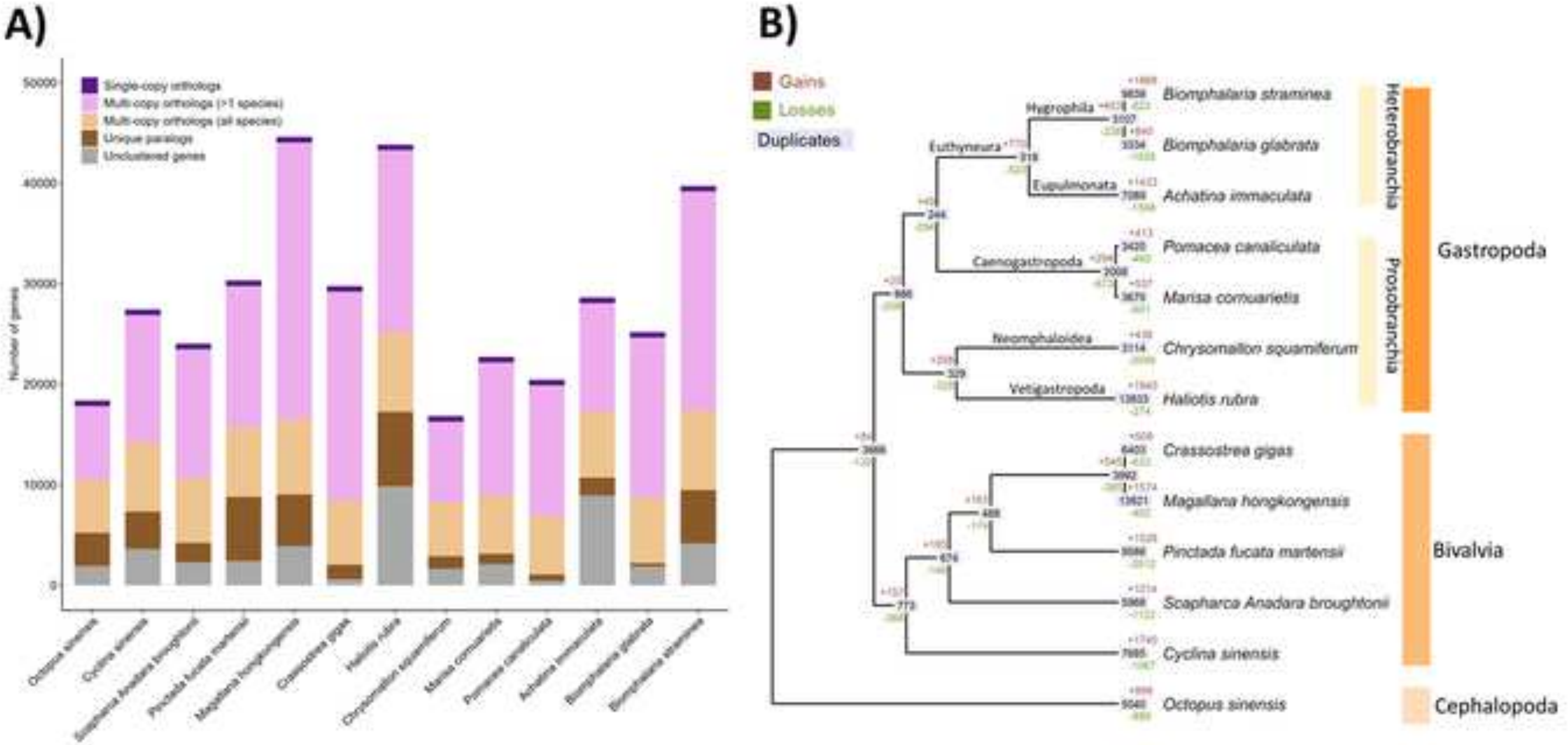

Fig 5

Figure 6 Click here to access/download;Figure;Fig6.TIF

A)

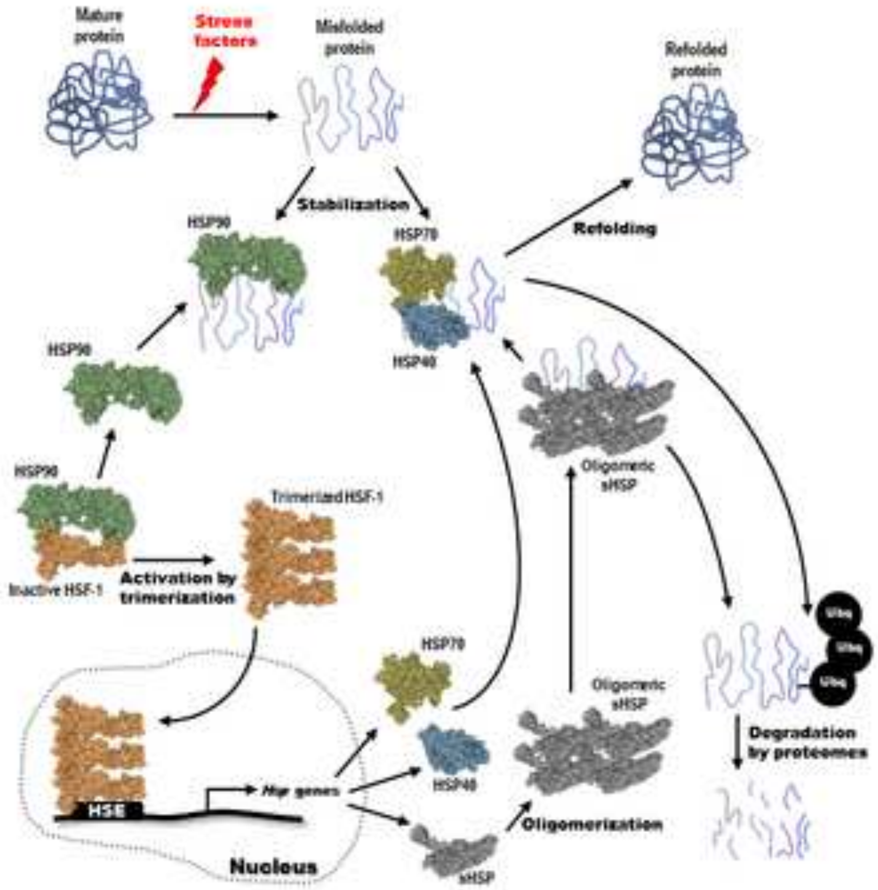

B)

Order	Family	Species	HSPE*	HSPB	HSP40	HSP70	HSPC (HSP90)	HSP110
Gastropoda	Planorbidae	*Biomphalaria straminea*	2	7	44	41	5	6
	Planorbidae	*Biomphalaria glabrata*	4	5	32	11	2	1
	Haliotidae	*Haliotis discus*	1	12	26	18	1	0
	Ampullariidae	*Lottia gigantea*	2	11	33	15	4	2
	Ampullariidae	*Lanistes nyassanus*	2	9	35	13	3	1
	Ampullariidae	*Marisa cornuarietis*	2	12	35	13	3	2
	Ampullariidae	*Pomacea canaliculata*	2	13	36	12	3	2
	Ampullariidae	*Pomacea maculata*	2	12	36	10	3	2
	Lymnaeidae	*Radix auricularia*	2	9	27	24	3	2
	Peltospiridae	*Chrysomallon squamiferum*	2	10	32	16	3	1
	Achatinidae	*Achatina fulica*	3	14	37	26	5	3
	Achatinidae	*Achatina immaculata*	4	14	33	22	5	4
Bivalvia	Ostreida	*Magallana hongkongensis*	2	20	50	123	5	2
	Ostreida	*Crassostrea gigas*	2	12	39	137	4	3
	Ostreida	*Crassostrea virginica*	3	18	50	142	4	2
	Ostreida	*Saccostrea glomerata*	2	13	40	110	3	1
	Mytilida	*Bathymodiolus platifrons*	2	7	40	103	3	2
	Mytilida	*Modiolus philippinarum*	2	14	38	87	3	2
	Pterriida	*Pinctada fucata*	2	12	35	89	3	3
	Pterriida	*P. f. martensii*	0	10	40	95	3	2
	Pectinida	*Mizuhopecten yessoensis*	2	8	36	61	3	2
	Venerida	*Cyclina sinensis*	4	12	42	77	5	2
	Arcida	*Scapharca (Anadara) broughtonii*	2	11	41	81	4	2
Cephalopoda	Octopodidae	*Octopus bimaculoides*	2	8	32	9	4	3
	/	*Architeuthis dux(squid)*	2	8	28	16	4	3
	/	*Octopus sinensis*	3	8	34	16	11	3
Annelida	Clitellata	*Helobdella robusta*	2	15	37	10	3	2
	Polychaete	*Capitella teleta*	2	23	34	34	4	2
Plathelminthes	Cestoda	*Echinococcus multilocularis*	2	4	25	49	6	6
	Trematoda	*Schistosoma mansoni*	2	11	25	6	3	2

Fig 6

Figure 7

Click here to access/download;Figure;Fig7.TIF

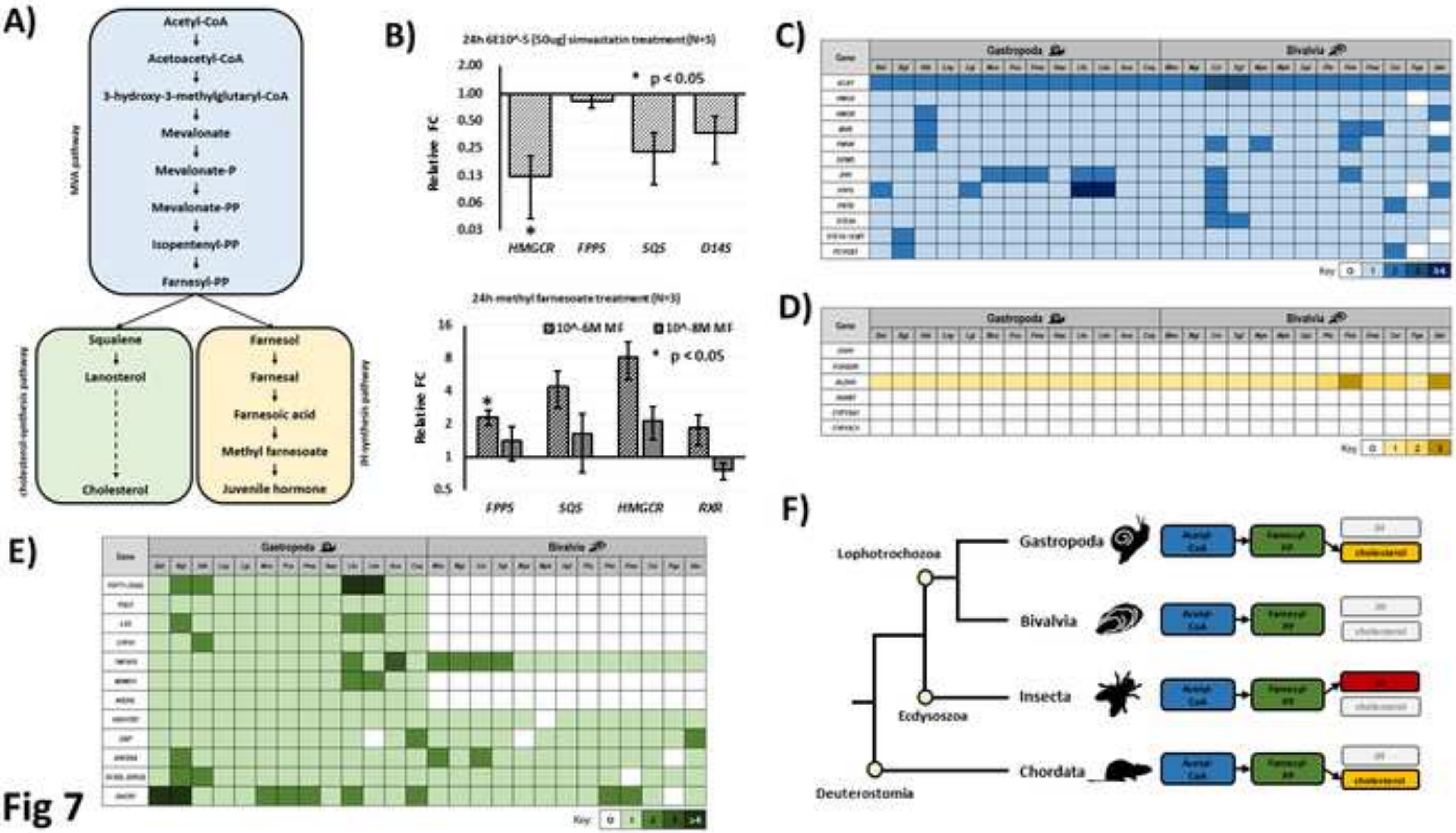

Supplementary S1

Click here to access/download
Supplementary Material
S1. Sequencing data.xls

Supplementary S2a

Click here to access/download
Supplementary Material
S2a_HboxSeqsSyntenyParaHox.xlsx

Supplementary S2b

Click here to access/download
Supplementary Material
S2b_B.glabrata_Hboxes.jpg

Supplementary S3

Click here to access/download
Supplementary Material
S3. Ecdysteroid.xlsx

Supplementary S4

Click here to access/download
Supplementary Material
S4. Insulin.xlsx

Supplementary S5

Click here to access/download
Supplementary Material
S5. Synteny.pptx

Click here to access/download
Supplementary Material
S6. Gene gain and loss.xlsx

Supplementary S7

Click here to access/download
Supplementary Material
S7. HSP.xlsx

Supplementary S8

Click here to access/download
Supplementary Material
S8. Cholesterol and sesquiterpenoid.xlsx

Supplementary S9

Click here to access/download

Supplementary Material

S9. Phylogentic trees.pptx

Click here to access/download
Supplementary Material
S10. Tables.pptx

Response to reviewers Click here to access/download;Personal Cover;Response.docx

Reviewer reports

Reviewer #1

This paper describes a new genome assembly for the snail *Biomphalaria straminea*. Reported contiguity is much higher even than the existing assemblies for the model snail in the genus, *B. glabrata*. This assembly will be a useful resource for research on *B. straminea* and for studies of the genus as a whole, including *B. glabrata* and the African snail vectors which have the largest impact on human health. The descriptions of gene families and synteny are insightful. Inclusion of experimental data (drug and hormone treatment) is unusual for a genome paper but not inappropriate. However these results are somewhat buried in the paper; there is no indication in the abstract that there is any RNA data, even though the authors sequenced the entire transcriptome and conducted RT-qPCR on candidate genes. The authors may wish to emphasize this aspect of the paper more strongly. In general, there is a lack of genomic resources for mollusks given the importance of the phylum (see Davison & Neiman 2021, Philos Trans R Soc Lond B Biol Sci), and this genome assembly is an important step toward meeting this need.

Response 1:

We thank the reviewer for the positive comments. The following sentence is now added in the Abstract-Findings (page 2, line 14):

"Transcriptomes from adults were also obtained".

And page 2, line 20-22:

"In addition, hormone treatment together with RT-qPCR assay reveal a sesquiterpenoid hormone responsive system in *B. straminea*, illustrating this renowned insect hormonal system is also present in the lophotrochozoan lineage."

Further, we have also added the following sentence to point out the lack of genomic resources of molluscs given the importance of this phylum as suggested (page 4, line 18-22):

"Whole genome sequences are valuable resources for obtaining deeper understanding of the biology of any organism. Despite the importance of the phylum Mollusca, there is a lack of genomic resources (Davison and Neiman 2021). In the case of *B. straminea*, such a resource will impact questions of how they may interact with *S. mansoni* and how similar the genetic mechanisms are between different *Biomphalaria* species, with possible implications for how treatments and management strategies might be transferable."

Minor comments:

Discussion of the African clade is confusing, as the authors don't explain that *B. glabrata* is neotropical but a phylogenetic sister to the African *Biomphalaria*, and no actual African snails were analyzed here. The point is simply that *B. straminea* and *B. glabrata* represent two major clades in the genus, and thus the conserved synteny between them suggests it

is conserved across the genus. It would be good to cite Campbell again in this section or another phylogenetic study (e.g. DeJong et al. 2001 Mol Biol Evol).

Response 2:

We have now included the references as suggested in the relevant sections, including page 4, line 3-4:

"The native range of *Biomphalaria* snails is South America and Africa (Campbell et al 2000; DeJong 2001)."

And page 16, line 15-17:

"Previous phylogenetic tree constructions for different *Biomphalaria* species suggested a monophyletic clade of African species with the remaining lineages being neotropical species (Campbell et al 2000; DeJong 2001)."

The statement "Perhaps more thorough sequencing of *B. glabrata* could determine if this is shared in the genus" is correct, but it would be even more useful to have genomes from additional *Biomphalaria* species, not just more *B. glabrata*.

Response 3:

We have now revised the sentence as suggested, as shown in page 15, line 19-21:

"Perhaps more thorough sequencing of *B. glabrata* or assemblies of additional *Biomphalaria* species could determine if this is shared in the genus, or if it is a novelty of *B. straminea*."

Reviewer #2:

This characterization of the genome of *Biomphalaria straminea*, invasive from South America to Hong Kong and main land China, provides a valuable contribtion to interpret biology of freshwater snail species of the genus Biomphalaria that vector the infectious disease human schistosomiasis. Caused by trematode parasites of the genus *Schistosoma*, the impact of schistosomiasis on global human health is second only to malaria. The data complement the previously reported *Biomphalaria* glabrata genome and is highly likely to drive extensive comparative genome analyses to inform disease vector biology and biology of molluscs in general.

The study performs logical analyses of the genome involving repetitive content, Hox gene complement and organization, synteny with available molluscan assemblies, gene expansions, presence of genes of metabolic/hormonal pathways. The latter includes an experimental work component.

Although several sequencing approaches are applied, the work did not yield a chromosome level assembly. The authors should discuss why not, and identify likely challenges to chromosome-level assembly for this snail species (repetitive content/genome

size?). The reviewer does not perceive this as weakness, rather an indication of complexity of gastropod (molluscan?) genomes.

Response 4:

We thank the reviewer for the careful reading. Given we had used different individuals for 10X Genomics and Hi-C to assemble this genome, we expect that some scaffolds cannot be mapped to the main pseudomolecules.

Also, comparison with other molluscan genomes is valuable but the presentation of the results is less effective because it does not clarify the taxonomic relations of the molluscs included in the study. It is recommended to provide a figure to clarify the taxonomy of Cephalopoda, Bivalvia and Gastropoda in this study. Within Gastropoda, also identify prosobranchs, heterobranchs, eupulmonates and hygrophila.

Response 5:

We have now modified the main figures (Figure 4 and 5) and incorporated the taxonomy as suggested.

Consistent use of deuterostomes, exdysozoan plus lophotrochozoan protostomes and prebilateria would improve clarity.

Response 6:

We have now amended the uses of these terms throughout the manuscript.

Finally, the description of the relevant parasitology is incorrect in places, see specific comments for corrections.

Overall, the methods appropriate to the aims of the study, additional details are needed to describe the experimental work with live snails.

Response 7:

The methodology for experimental work with live snails are now included, as shown in page 22, line 3-6:

"One week prior to the experiment, approximately 100 ramshore snails were collected in a freshwater stream in Tai Po New Territories, Hong Kong (GPS: 22.50206300747975, 114.15354682258841). The collected animals were kept and maintained in the laboratory aquarium and fed with lettuce three days a week."

And page 28, line 1-2:

“Experimental adult animals of ~1cm with reproductive capability were isolated from the culture and were rinsed in double-distilled water to remove any contaminants.”

The conclusions are adequately supported by the data, although the use of a single stimulus for gene expression of ecdysteroid pathway genes does not seem to equate systemic analysis, also considering the lack of DE.

Response 8:

We agree with the reviewer, and have taken out the figure accordingly.

Quality of language is generally acceptable, specific comments follow below for editing of language in the manuscript.

No specific methods section is provided but this reviewer is not concerned.

specific comments

P1 Title, intermediate vector is incorrect, change to (obligate) intermediate host or vector

Response 9:

The title is now changed to “Genome of the ramshorn snail *Biomphalaria straminea* - an obligate intermediate host of schistosomiasis”

P2 Abstract L6 B. stramina IS native to South America

Response 10:

The word “was” is now corrected to “is”.

L7 Reservoir is a parasitological term used for alternative host species, other than the main hosts for a parasite. It is not appropriate for the snail host. Suggest replace "reservoir" by "vector host"

Response 11:

The word “reservoir” is now changed to “vector host” as suggested.

L9 suggest edit hinders to limits

Response 12:

The word “hinders” is now changed to “limits” as suggested.

L15-16, indicate range of "other molluscs", does it include the classes gastropoda, bivalvia and cephalopoda?

Response 13:

The sentence is now changed as suggested, and shown as follows:

“Comparisons with other mollusc genomes (including Gastropoda, Bivalvia and Cephalopoda) revealed syntenic conservation, patterns of homeobox gene linkage indicative of evolutionary changes to gene clusters, expansion of heat shock protein genes, and the presence of sesquiterpenoid and cholesterol metabolic pathway genes in Gastropoda.”

L18, again specify certain mollusc lineages

Response 14:

The sentence is now changed as suggested, and shown as follows:

“Comparisons with other mollusc genomes (including Gastropoda, Bivalvia and Cephalopoda) revealed syntenic conservation, patterns of homeobox gene linkage indicative of evolutionary changes to gene clusters, expansion of heat shock protein genes, and the presence of sesquiterpenoid and cholesterol metabolic pathway genes in Gastropoda.”

L21 suggest edit to biology related to snail vectors of schistosomiasis

Response 15:

The sentence is now changed to:

“This study provides the first genome assembly for the snail *B. straminea* and offers an unprecedented opportunity to address a variety of biology related to snail vectors of schistosomiasis, as well as evolutionary and genomics questions related to molluscs more widely.”

P3 L2 240 million are actually infected, these would require treatment, not preventive treatment

Response 16:

The word “preventive” is now deleted.

L9-10 these eggs do NOT circulate to the liver and leave the body. Eggs are deposited in blood venules and cross the intestinal wall to leave the body with faeces. Eggs that fail to do so are called reflux eggs and these circulate to the liver where they cause pathology. Please correct.

Response 17:

The biology and life cycle of *Schistosoma mansoni* is now corrected in page 3, line 13-16:

"Once inside the human body, adult worms lay eggs, which are deposited in the blood venules and will cross the intestinal wall to leave the body in the faeces. In the meantime, eggs that fail to cross the wall (named "reflux eggs") circulate to the liver where they grow, emerge, and cause pathology."

L10 delete contaminated

Response 18:

The word "contaminated" has been deleted.

L12 delete further

Response 19:

The word "further" is deleted.

L14-15 edit infected by to potential vectors for

Response 20:

The phrase "infected by" is now changed to "the potential vectors for" as suggested.

L17-18, For native range also cite "DeJong RJ, et al. Evolutionary relationships and biogeography of Biomphalaria (Gastropoda: Planorbidae) with implications regarding its role as host of the human bloodfluke, Schistosoma mansoni. Mol Biol Evol. 2001 Dec;18(12):2225-39. doi: 10.1093/oxfordjournals.molbev.a003769. PMID: 11719572."

Response 21:

We have now included the reference as suggested in page 4, line 3-4:

“The native range of *Biomphalaria* snails is South America and Africa (Campbell et al 2000; DeJong 2001).”

P4 L4--- Indicate whether parasite was introduced by infected snails, humans or other reservoir hosts.

Response 22:

The information is now added in page 4, line 12-17 as follows:

“According to the records from the database of the National Notifiable Disease Report System (NNDRS), 355 cases of imported schistosomiasis cases had been reported in 15 provinces in China between 1979 and 2019, including 78 cases infected with *S. mansoni*, 262 cases with *S. haematobia*, and 15 cases with unidentified *Schistosoma*. Since *B. straminea* had already been discovered in Guangdong province in southern China, it is believed that the imported *S. mansoni* increases the risk of its transmission in China. (Zhu and Xu 2014; Wang et al 2020).

P5 L3 Unclear with methods(P19,L13-18) indicate how many snails were used in total, only 1 or more because of using 1 adult snail for each of the sequencing methods. Also indicate source of snails; field isolate (date, latitude longitude of collection), wild type or lab isolate (inbred, outbred, kept for how many generations?).

Response 23:

The following details are now included in the Methods section.

“One week prior to the experiment, approximately 100 ramshore snails were collected in a freshwater stream in Tai Po New Territories, Hong Kong (GPS: 22.50206300747975, 114.15354682258841). The collected animals were kept and maintained in the laboratory aquarium and fed with lettuce three days a week.”

L5 is the compete mitogenome included as one of the scaffolds

Response 24:

The mitochondrial genome is not included as one of the scaffolds, and is now stated:

“The genome assembly (without the mitochondrial genome) is 1.005 Gbp with a scaffold N50 of 25.3 Mbp (Figure 1B).”

P6 L3, here and in figure, explain "spike in activity", is this a large number of sequences with only few lethal mutations, how is this shown in figure 1C?

Response 25:

The explanation is now added and shown as follows:

“The recent spike is evident from the relatively large percentage of repeats in the genome that are separated from their family consensus sequences by short distances, while the long tail of increasing divergence from consensus is suggestive of a gradual increase in activity of a relatively long time period (Figure 1C).”

P7 L1 "genes coopted to novel structures", explain, are these genes coding for proteins with novel structures or proteins with novel functions?

Response 26:

The sentence is now changed to:

“Hox genes have been co-opted to the development of novel morphological structures in cephalopods (Lee et al., 2003), and this corresponds to a breakdown of the Hox cluster across several chromosomes, and the loss of a few genes (Albertin et al., 2015).”

L5 "another oyster" provide Genus and species

Response 27:

Another oyster is the *Magallana hongkongensis*, which is now stated in the text as follows:

“Other mollusc genomes show a breakdown of homeobox clustering overall, like the Pacific oyster (*Crassostrea gigas*; Paps et al., 2015), while a more recent chromosome-level assembly reveals large-scale patterns of linkage in *Magallana hongkongensis* (Li et al., 2020).”

L6, indicate that Mollusca belong to lophotrochozoa, a main lineage of protostomes along with ecdysozoa and indicate that the vertebrates belong to deuterostomia.

Response 28:

The following sentences are now added:

“This genome assembly of *B. straminea* improves our understanding of homeobox gene linkage in comparison to other molluscs, which are lophotrochozoans and, alongside and well-studied ecdysozoans like flies, provide a more thorough protostome comparison to vertebrates, which are within the Deuterostomia.”

L10, and elsewhere (e.g. P9, L3-6) , define clustered and linked, what is the difference?

Response 29:

The sentences are now amended to clarify the situation:

"Many of these genes are clustered (situated on the same chromosome with no or very few non-homeobox genes in between) or linked (on the same chromosome, but with intervening non-homeobox genes) in the genome (Figure 2)."

L12 identify L. gigantea as gastropod

Response 30:

The sentence is now changed as follows:

"In an ordered cluster as seen in the gastropod, *L. gigantea*, for instance, the Hox genes are situated in the genome in the ancestral bilaterian order from anterior-acting *Hox1* to posterior-acting *Post1*, and no other non-Hox genes are found amongst the Hox genes (Simakov et al., 2013)."

L13 indicate level of ancestor (gastropod, molluscan or lophotrochozoan…)

Response 31:

The indication is now added.

P10 L19 indicate classes for limpet and oyster (Gastropoda and Bivalvia, respectively

Response 32:

They are now clearly indicated in the text and shown as follows:

"Perhaps surprisingly, none of the *Biomphalaria Irx* genes, *Irx1* (*a* and *b*), *Irx2*, *Irx3*, and *Irx4*, show clear orthology to specific gastropod (limpet) or bivalve (oyster) genes in a phylogenetic tree (Supplementary information S2c)."

P11 L14 define widely found, perhaps as broadly distributed?

Response 33:

The phrase "widely found" is now changed to "broadly distributed".

P14 L13 suggest include "or assemblies of additional Biomphalaria species"

Response 34:

The phrase “or assemblies of additional *Biomphalaria* species” is now added in the text.

L14 what are potential impacts?

Response 35:

The potential impact of the identified Hox rearrangement is that “the cluster rearrangements could indicate the loss of shared regulatory elements that constrain Hox clusters in other lineages and may reflect changes to Hox gene expression, perhaps underpinning developmental changes in these snails.”, which this is now added in the text.

L16 "good markers", to what extent?

Response 36:

The reason that homeobox gene is a “good marker” for the analyses of genome organization is because of its nature of clustering, linkage, and rearrangement. This concept is now added in the text.

L18-21. This interesting comparative genome analyses may deserve a figure?

Response 37:

A figure for *B. straminea* is added in the main Figure 2, while another one for *B. glabrata* is made as a supplementary figure (Supplementary S2b).

L22-23 define "mollusc genomes", perhaps "as available from GenBank for gastropod, bivalve and cephalopod molluscs.

Response 38:

The sentence is now changed as follows:

“To examine the syntenic relationships more generally between *Biomphalaria* and mollusc genomes, we constructed Oxford dot-plots, comparing the chromosomal positions of orthologous genes between published mollusc genomes, as available from GenBank for gastropod, bivalve and cephalopod molluscs.”

P15 L1-2 does the number of pseudochromosomes resemble the number of chromosomes shown by karyotyping B. glabrata (see e.g Adema et al., 2017)

Response 39:

The pseudochromosomes identified in *B. straminea* (2n=36) resembles that in the karyotyping of *B. glabrata*, and is now added in the text as follows:

"As shown in Figure 4, the relationship of pseudo-chromosomes (2n=36, Adema et al 2017) and scaffolds between *B. straminea* and molluscs of other classes were conserved in most cases."

L2 edit genus to genera, and perhaps "of other classes" (see previous comment)

Response 40:

The term "genus" is now changed to "of other classes" as suggested.

L3-4. cite studies by Campbell and DeJong, mentioned above.

Response 41:

The citation is now added accordingly.

L8 identify Achatina as eupulmonata gastropod, also this species of stylommatophora (landsnail) was proposed to have undergone whole genome duplication, does this complicate the synteny comparison?

Response 42:

Achatina is now identified as the eupulmonata gastropod in the text.

We also thank the reviewer for raising the concern on the whole genome duplication (WGD) of this species. In short, we are using the orthologues called by mutual best hit between two species in the analysis. Paralogs from different chromosome were included in the plot (Simakov et al 2020). This allows the identification of WGD or chromosome duplication after separating from the last common ancestor. So the WGD in *A. immaculata* would not complicate the comparison carried out in this study.

Simakov O, Marlétaz F, Yue JX, O'Connell B, Jenkins J, Brandt A, et al.. Deeply conserved synteny resolves early events in vertebrate evolution. Nat Ecol Evol. 2020; doi: 10.1038/s41559-020-1156-z.

L11 indicate that this would be an ancestor of the Hygrophila and Eupulmonata

Response 43:

The sentence is now changed as follows:

"These patterns indicated that some chromosome duplication and alteration occurred from the most recent common ancestor of *B. straminea*, *B. glabrata* and *A. immaculata* (the ancestor of Hygrophila and Eupulmonata)."

L12-14, for accuracy, text actually lists molluscs with greater phylogenetic distance (prosobranch gastropods and Bivalvia), revise accordingly.

Response 44:

Here, we are describing the distance among these four species rather than each one against this snail species. For clarification, we have amended the sentence to avoid misleading information shown as follows:

"Further, species with closer evolutionary distance shared more similar synteny patterns against *B. straminea* (for example, between *Pomacea canaliculata* and *Marisa cornuarietis*, as well as between *Crassostrea gigas* and *M. hongkongensis*, which they shared more similar synteny blocks), suggesting the dynamic changes of chromosomes arrangement in different molluscs."

L16-17 provide citation for divergence and ancestor?

Response 45:

The following citation is now included:

"Smith et al (2011). Resolving the evolutionary relationships of molluscs with phylogenomic tools. Nature, 480(7377), 364-367.".

P16 L7-10. Suggest to remove fig 3C, the text adequately conveys the lack of altered expression. Indicate what other organisms do respond to the stimulus employed, suggesting differences in biology?

Response 46:

Figure 3C is now removed as suggested, and the following text are also added to indicate other organisms that do respond to the stimulus employed:

"Similar hormone treatments have been shown to elicit the downstream genes in insects in previous studies (Hossain et al, 2013; Sekimoto et al 2007; Ji et al 2021)."

Ji, C., Zhang, N., Jiang, H., Meng, X., Ge, H., Yang, X., ... & Wang, J. (2021). 20-hydroxyecdysone regulates expression of methioninesulfoxide reductases through transcription factor FOXO in the red flour beetle, Tribolium castaneum. Insect Biochemistry and Molecular Biology, 131, 103546.

Hossain, M. S., Liu, Y., Zhou, S., Li, K., Tian, L., & Li, S. (2013). 20-Hydroxyecdysone-induced transcriptional activity of FoxO upregulates brummer and acid lipase-1 and promotes lipolysis in Bombyx fat body. Insect biochemistry and molecular biology, 43(9), 829-838.

Sekimoto, T., Iwami, M., & Sakurai, S. (2007). 20- Hydroxyecdysone regulation of two isoforms of the Ets transcription factor E74 gene in programmed cell death in the silkworm anterior silk gland. Insect molecular biology, 16(5), 581-590.

L19 define high memory scores

Response 47:

The phrase “high memory scores” is now changed to “better associative learning behaviour” for a clearer and better description.

P17 L7, Is this pattern similar or both Biomphalaria species?

Response 48:

The following text is now amended to address this concern:

“Gene family analysis among these genomes revealed the expansion of 1,869 orthogroups and contraction of 623 orthogroups in *B. straminea,* while in *B. glabrata*, the expansion of 840 orthogroups and contraction of 1,035 orthogroups (Figure 5). This data highlights the importance of having the *B. straminea* genomic resource, and potentially suggested that specific control strategies might be needed for *B. straminea* rather than treating it as identical to *B. glabrata.*”

L11 suggest edit "in certain" to "among"

Response 49:

The phrase “in certain” is now changed to “among”.

L16 "its adaptation" to what?

Response 50:

We have added extra information as suggested and shown as follows:

"In some mollusc genomes, such as that of the Pacific oyster *Crassostrea gigas*, an expansion of heat shock protein 70 (HSP70) has been observed in the genome and hypothesized to be important to its adaptation to changes in ambient environmental factors or pressures (Zhang et al 2012)."

L17 (also P18, L3-4) Are these HSPs (types and numbers) in accordance with the B. glabrata genome, Adema et al 2017?

Response 51:

The HSPs we identified here is based on our genome analyses, while those identified by Adema et al 2017 is mainly on the established proteome. So the number is not identical.

P18 L15 suggest edit "can change" to "changed"

Response 52:

The phrase "can change" is now edited to "changed".

P19, L3 suggest edit "holding" to "having"

Response 53:

The word is changed accordingly.

L4 "dynamics" is not clear, revise to other word?

Response 54:

The word "dynamics" is now deleted.

L6 edit to ….. glabrata as well as among other molluscan taxa. Or similar

Response 55:

The sentence is now edited as follows:

"Our work provides gene and transposable element annotations, and detailed analyses of a variety of gene families, including the homeobox, ecdysteroid, insulin, heat shock protein, and sesquiterpenoid pathway genes, suggesting extensive molecular differences between *B.*

straminea and *B. glabrata* as well as among other molluscan taxa. More generally, our high-quality *B. straminea* genome provides a useful reference point for further understanding of the biology, ecology and evolution of molluscs."

P23 L1-2 How were the initial sequences used for the searches selected?

Response 56:

The initial sequences were retrieved from NCBI, and now stated in the text:

"Gene family sequences were first obtained from NCBI of neighboring species, including *B. glabrata* and other lophotrochozoans. The sequences were then used to retrieve the corresponding genes from the *B. straminea* genome using the tBLASTn algorithm on a local server, with an E-value smaller of 10^{-3}."

L14-17, add idnetifiers for the species used as deuterostomes, and ecdysozoan or lophotrochozoan protostomes, also indicate oyster is bivalvia, limpet is gastropod.

Response 57:

The identifiers are now added to each of the described animals, shown as follows:

"Alignments of each class were made using MUSCLE (Edgar, 2004), with homeodomain sequences from human (*Homo sapiens*, deuterostome), amphioxus (*Branchiostoma floridae*, a cephalochordate), the ecdysozoans fruitfly (*Drosophila melanogaster*), and red flour beetle (*Tribolium castaneum*), and the lophotrochozoans oyster (*Crassostrea gigas*, bivalve), limpet (*Lottia gigantea*, gastropod),brachiopod (*Lingula anatina*,), andannelids *Platynereis dumerilii* and *Capitella teleta*, where available from other studies (Paps et al., 2015; Barton-Owen et al., 2018) and HomeoDB (Ying-Fu et al., 2011; 2008)."

P25 L9 define adult by shell diameter size, or confirmed by reproductive maturity. How was culture initiated? was this from a multiple individual collected from the field or from a single snail by selfing? For how long was the culture maintained at time of experiments?

Response 58:

The adult snail is defined by their diameter size. The culture was initiated by capturing multiple snails in the wild and they were brought back to the lab for short-term culture. The culture lasts around for half a year during the conduction of experiment. The details are now added in the methodology section as follows:

"One week prior to the experiment, approximately 100 ramshore snails were collected in a freshwater stream in Tai Po New Territories, Hong Kong (GPS: 22.50206300747975,

114.15354682258841). The collected animals were kept and maintained in the laboratory aquarium and fed with lettuce three days a week."

P26 provide primers used for the gene targets. Was target specificity tested? Myoglobin may have sequence similarity with the multi domain sequences of Biomphalaria hemoglobin.

Response 59:

The following information are now added in the text:

"Primer details are listed in Supplementary File S8. The primers were tested by conventional PCR with *B. straminea* cDNA prior to experiments to ensure its specificity. Each sample was analyzed in replicates. The expression of each target gene transcript was normalized to the housekeeping gene, myoglobin (Myo), as adopted in previous studies (Jiang et al 2006; Arican-Goktas et al 2014; Queiroz et al 2017; Pinaud et al 2021). The subsequent fold induction analyses were calculated using the ΔΔCt method."

Figure 4 identify in figure branches of heterobranchs, prosobranchs,gastropods, bivalves and cephalopods. (similar to fig 5b)

Response 60:

The figure branches are now labelled with the corresponding lineages as suggested.

Again can the number of (pseudo)chromosome clusters be equated with chromosome numbers recorded for Biomphalaria sp?

Response 61:

Yes, the pseudochromosome identified by our genome assembly is congruent with *Biomphalaria glabrata* karyotype and genome data. Kindly see Response 39.

Fig 7B indicate branches for lophotrochozoan protostomes, ecdysozoan protostomes and deuterostomes

Response 62:

The figure branches are now labelled with the corresponding lineages as suggested.
