# Supplementary material for: Genome of the ramshorn snail Biomphalaria straminea—an obligate intermediate host of schistosomiasis
Source: Gigascience. 2022 Feb 15;11:giac012. doi: 10.1093/gigascience/giac012 (PMC8848322; doi:10.1093/gigascience/giac012)
Supplement: giac012_Supplemental_Files [file giac012_supplemental_files.zip › S10. Tables.pptx]

## Slide 1
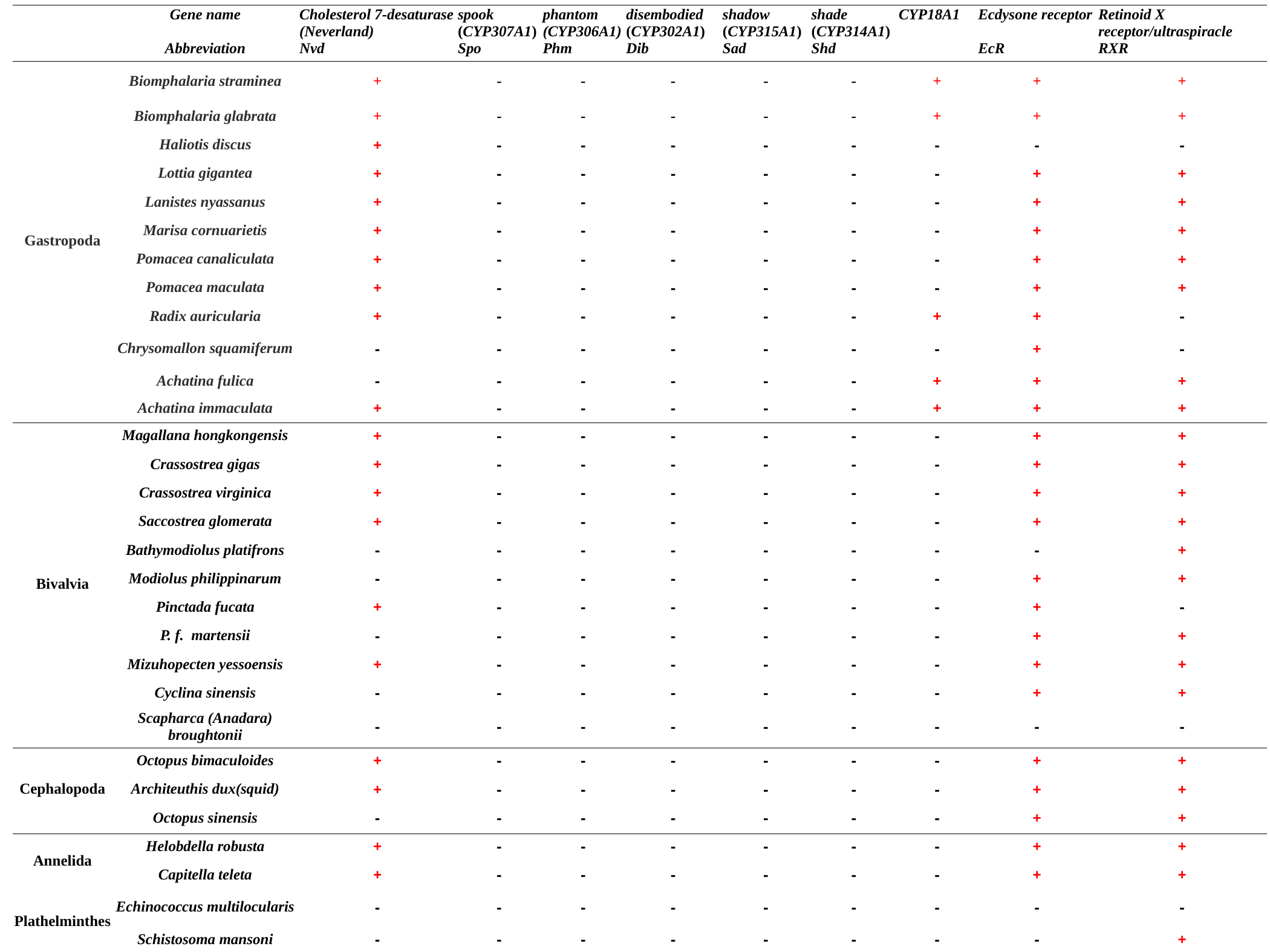

| | Gene name Abbreviation | Cholesterol 7-desaturase (Neverland) Nvd | spook (CYP307A1) Spo | phantom (CYP306A1) Phm | disembodied (CYP302A1) Dib | shadow (CYP315A1) Sad | shade (CYP314A1) Shd | CYP18A1 | Ecdysone receptor EcR | Retinoid X receptor/ultraspiracle RXR |
| --- | --- | --- | --- | --- | --- | --- | --- | --- | --- | --- |
| Gastropoda | Biomphalaria straminea | + | - | - | - | - | - | + | + | + |
| | Biomphalaria glabrata | + | - | - | - | - | - | + | + | + |
| | Haliotis discus | + | - | - | - | - | - | - | - | - |
| | Lottia gigantea | + | - | - | - | - | - | - | + | + |
| | Lanistes nyassanus | + | - | - | - | - | - | - | + | + |
| | Marisa cornuarietis | + | - | - | - | - | - | - | + | + |
| | Pomacea canaliculata | + | - | - | - | - | - | - | + | + |
| | Pomacea maculata | + | - | - | - | - | - | - | + | + |
| | Radix auricularia | + | - | - | - | - | - | + | + | - |
| | Chrysomallon squamiferum | - | - | - | - | - | - | - | + | - |
| | Achatina fulica | - | - | - | - | - | - | + | + | + |
| | Achatina immaculata | + | - | - | - | - | - | + | + | + |
| Bivalvia | Magallana hongkongensis | + | - | - | - | - | - | - | + | + |
| | Crassostrea gigas | + | - | - | - | - | - | - | + | + |
| | Crassostrea virginica | + | - | - | - | - | - | - | + | + |
| | Saccostrea glomerata | + | - | - | - | - | - | - | + | + |
| | Bathymodiolus platifrons | - | - | - | - | - | - | - | - | + |
| | Modiolus philippinarum | - | - | - | - | - | - | - | + | + |
| | Pinctada fucata | + | - | - | - | - | - | - | + | - |
| | P. f. martensii | - | - | - | - | - | - | - | + | + |
| | Mizuhopecten yessoensis | + | - | - | - | - | - | - | + | + |
| | Cyclina sinensis | - | - | - | - | - | - | - | + | + |
| | Scapharca (Anadara) broughtonii | - | - | - | - | - | - | - | - | - |
| Cephalopoda | Octopus bimaculoides | + | - | - | - | - | - | - | + | + |
| | Architeuthis dux(squid) | + | - | - | - | - | - | - | + | + |
| | Octopus sinensis | - | - | - | - | - | - | - | + | + |
| Annelida | Helobdella robusta | + | - | - | - | - | - | - | + | + |
| | Capitella teleta | + | - | - | - | - | - | - | + | + |
| Plathelminthes | Echinococcus multilocularis | - | - | - | - | - | - | - | - | - |
| | Schistosoma mansoni | - | - | - | - | - | - | - | - | + |
