# Supplementary material for: Genome of the ramshorn snail Biomphalaria straminea—an obligate intermediate host of schistosomiasis
Source: Gigascience. 2022 Feb 15;11:giac012. doi: 10.1093/gigascience/giac012 (PMC8848322; doi:10.1093/gigascience/giac012)
Supplement: giac012_Supplemental_Files [file giac012_supplemental_files.zip › S9. Phylogentic trees.pptx]

## Slide 1
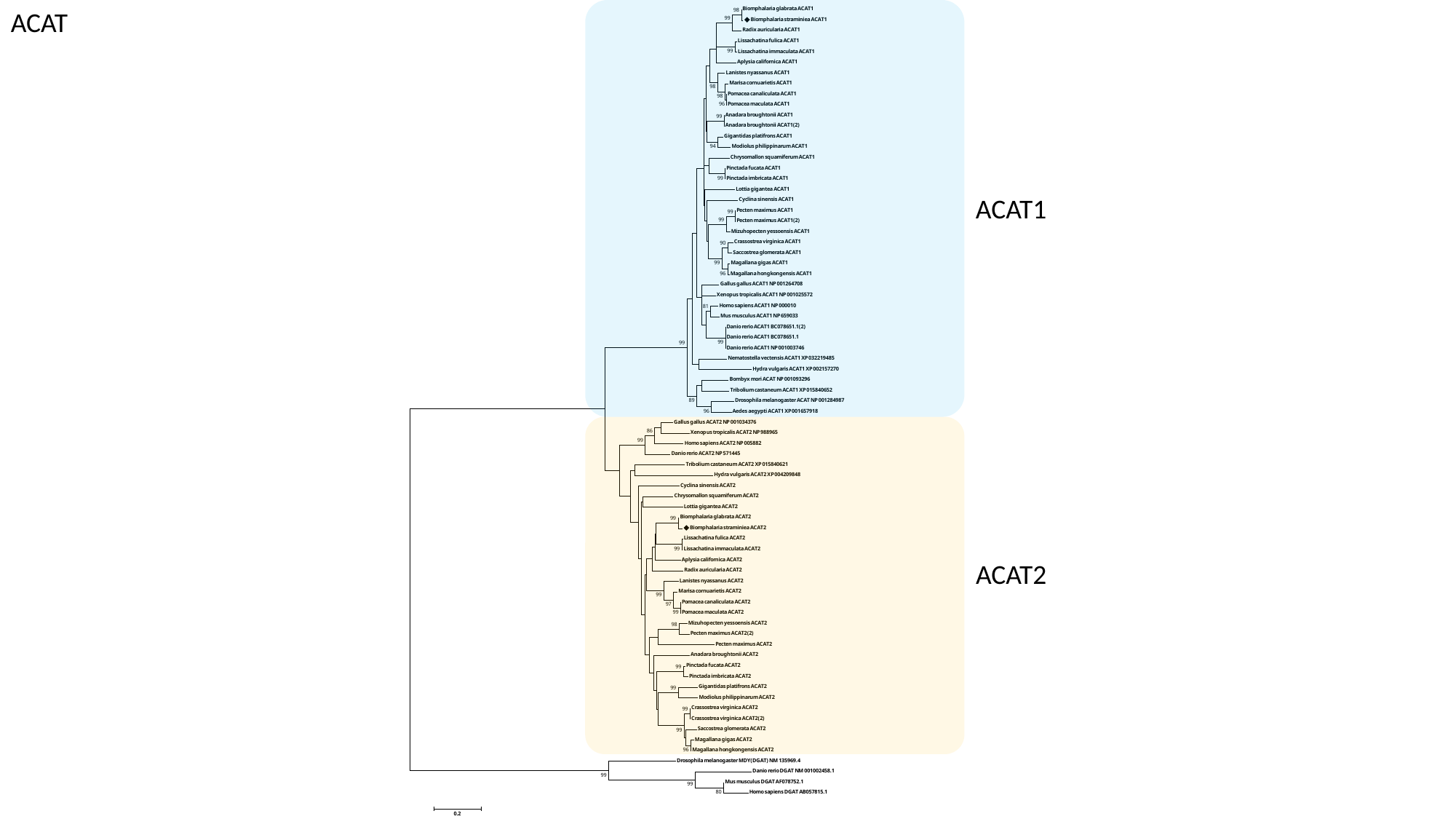

ACAT
ACAT1
ACAT2

## Slide 2
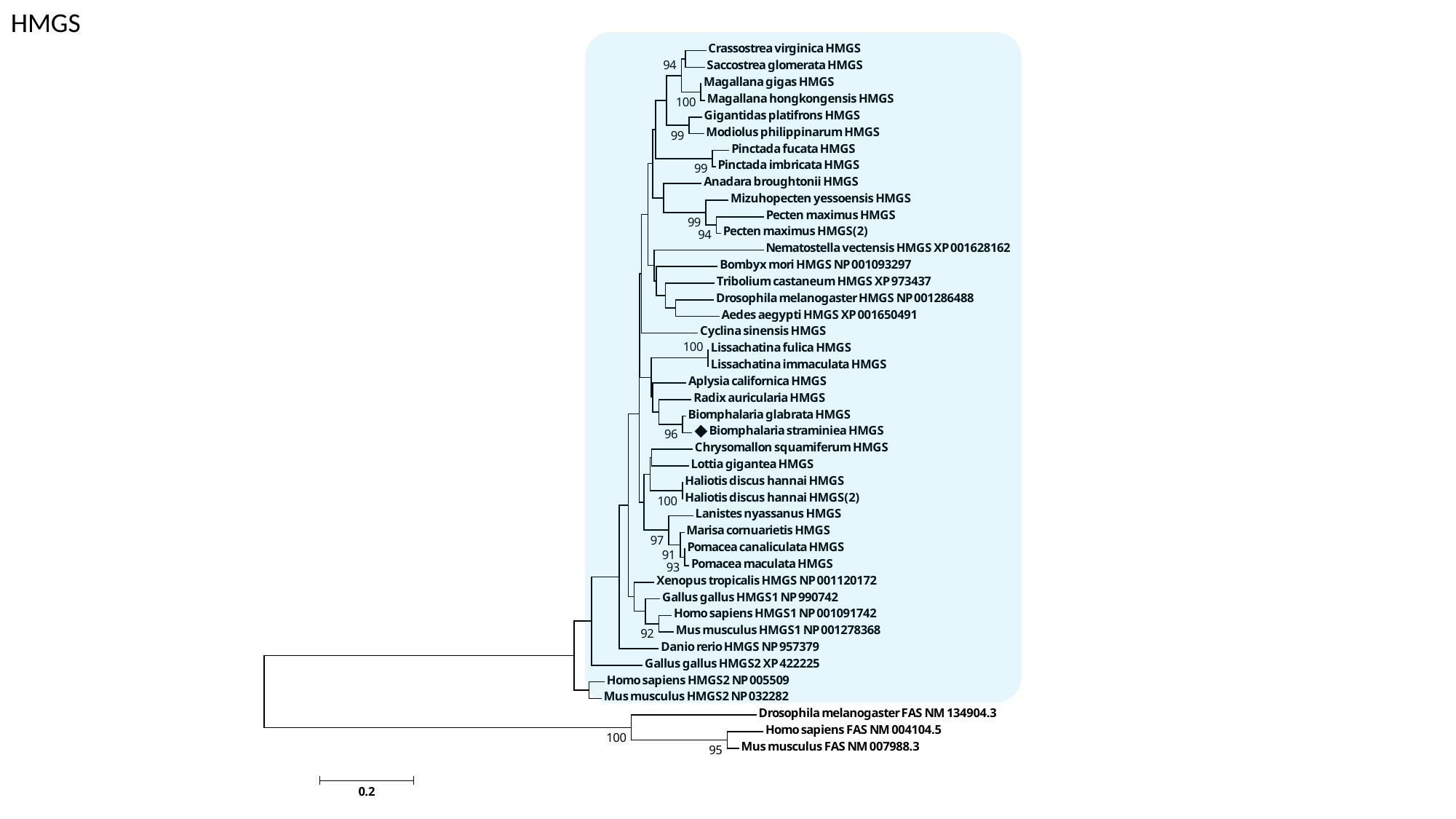

HMGS

## Slide 3
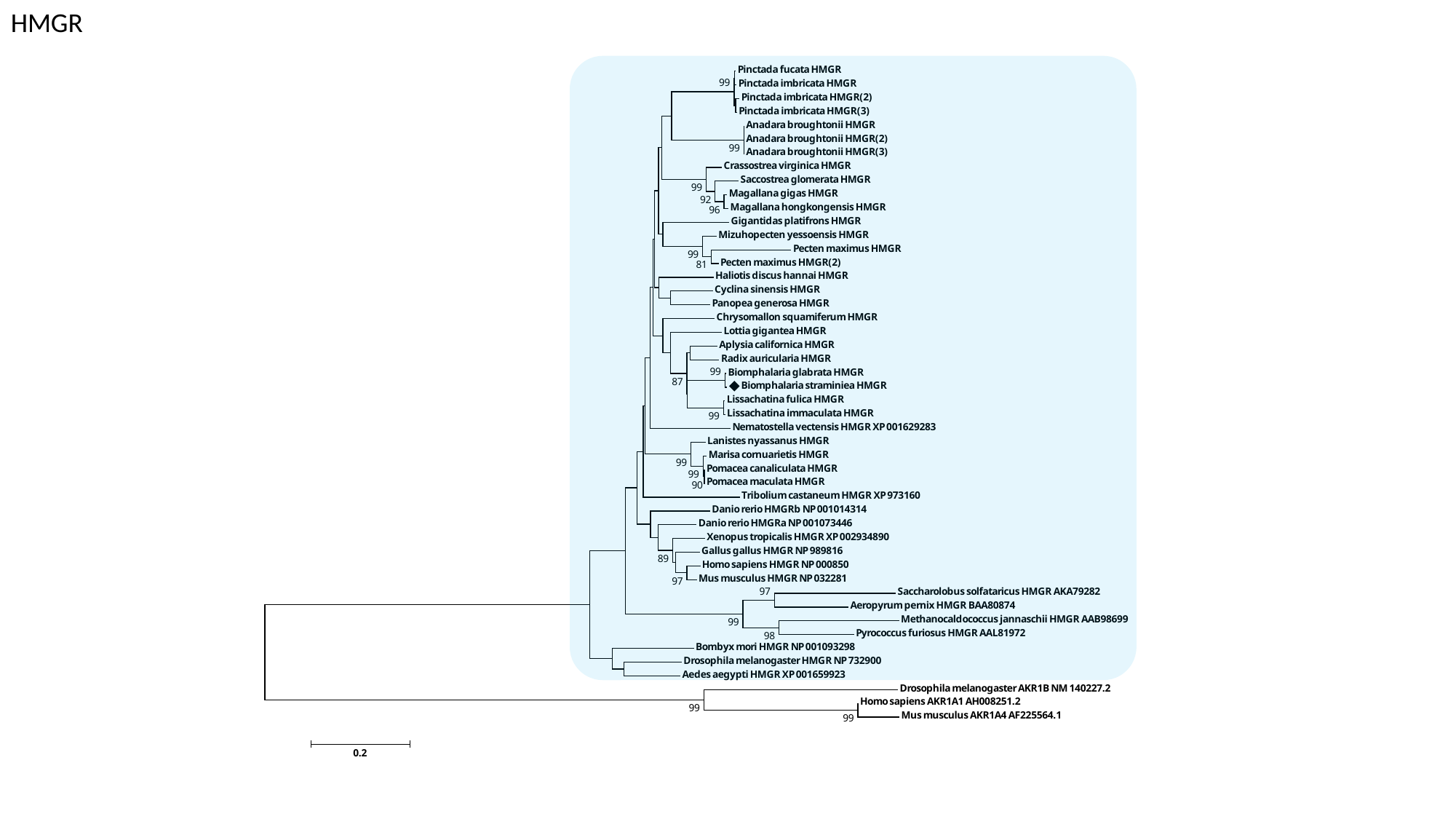

HMGR

## Slide 4
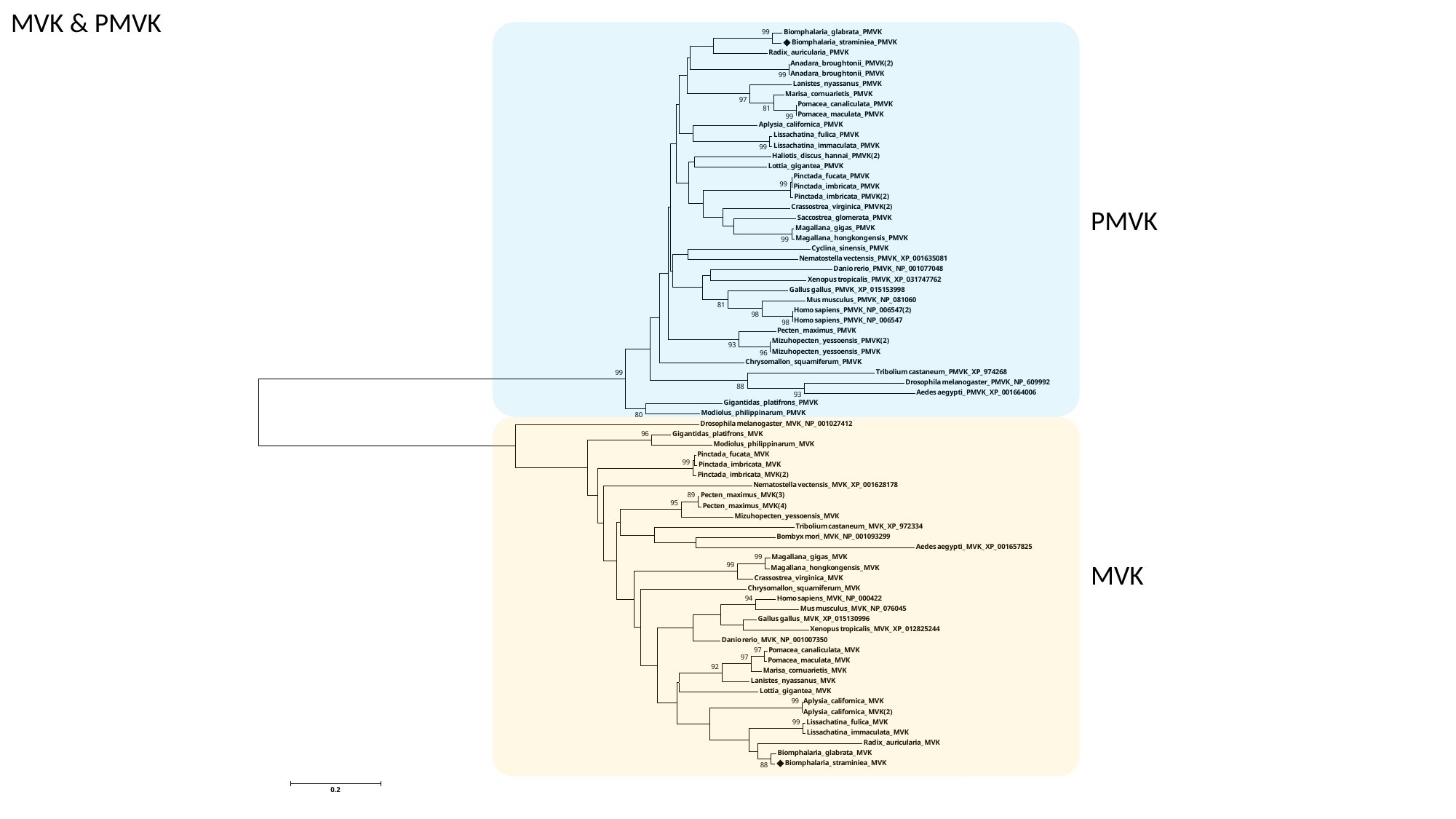

MVK & PMVK
PMVK
MVK

## Slide 5
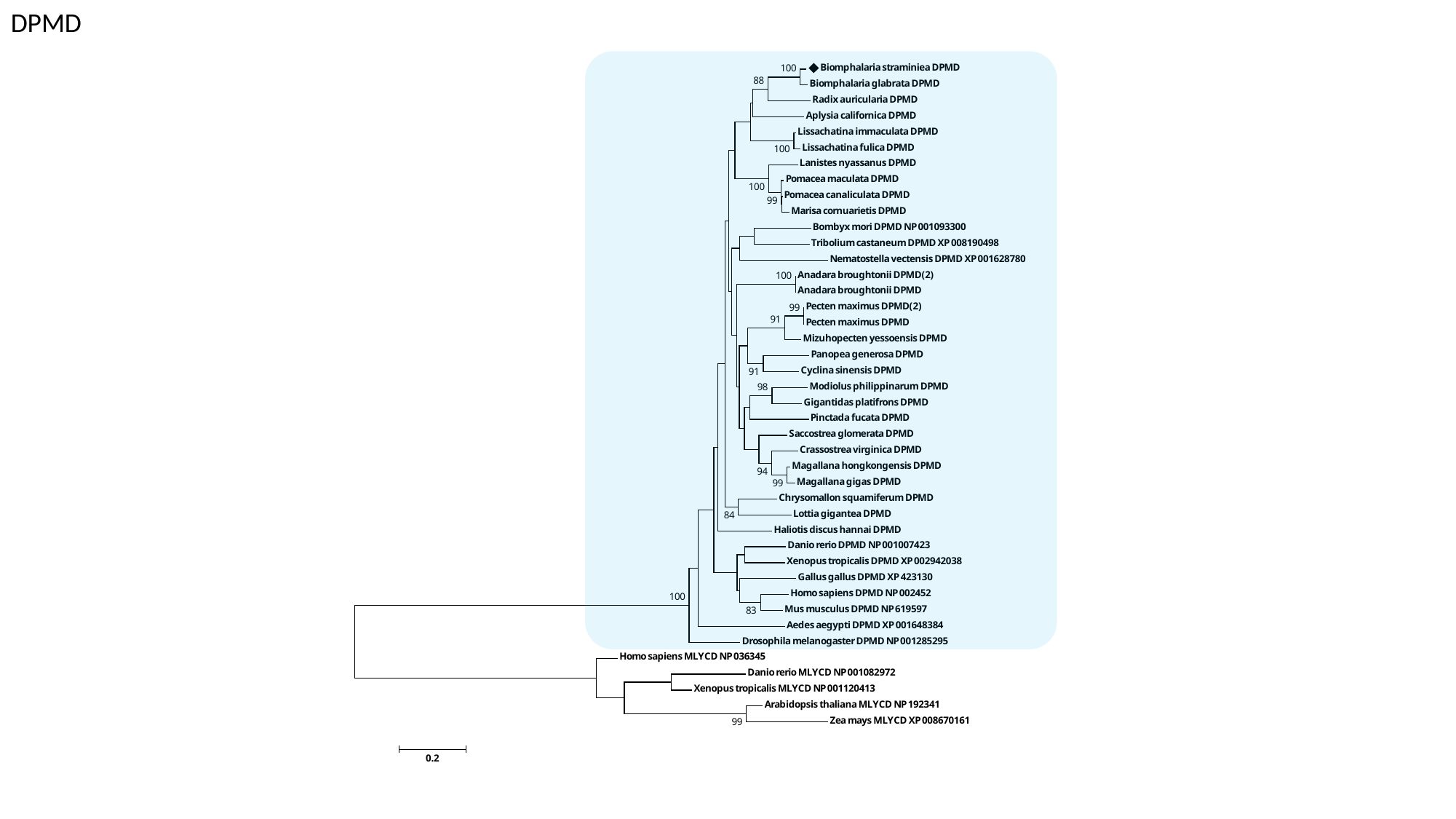

DPMD

## Slide 6
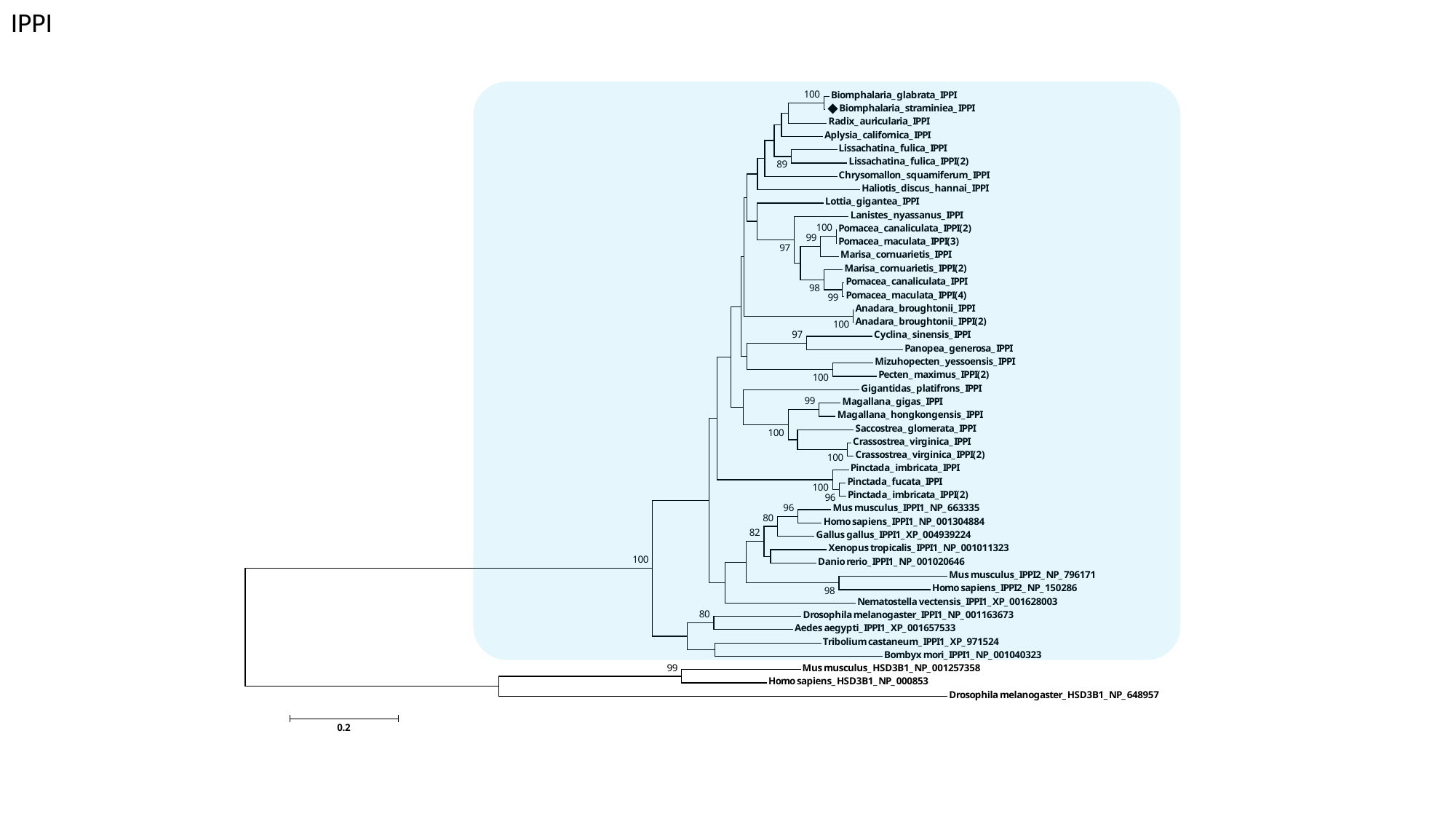

IPPI

## Slide 7
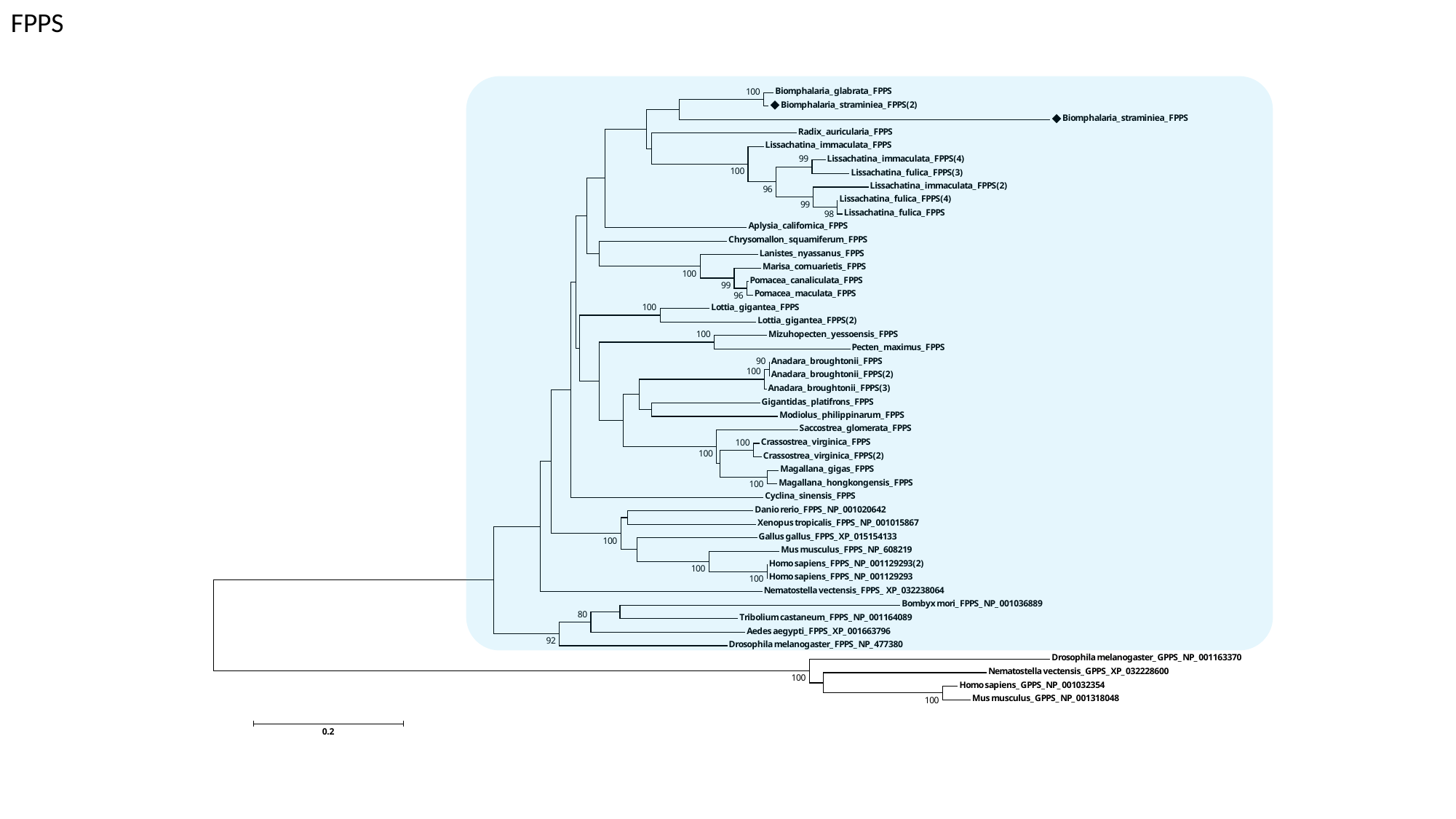

FPPS

## Slide 8
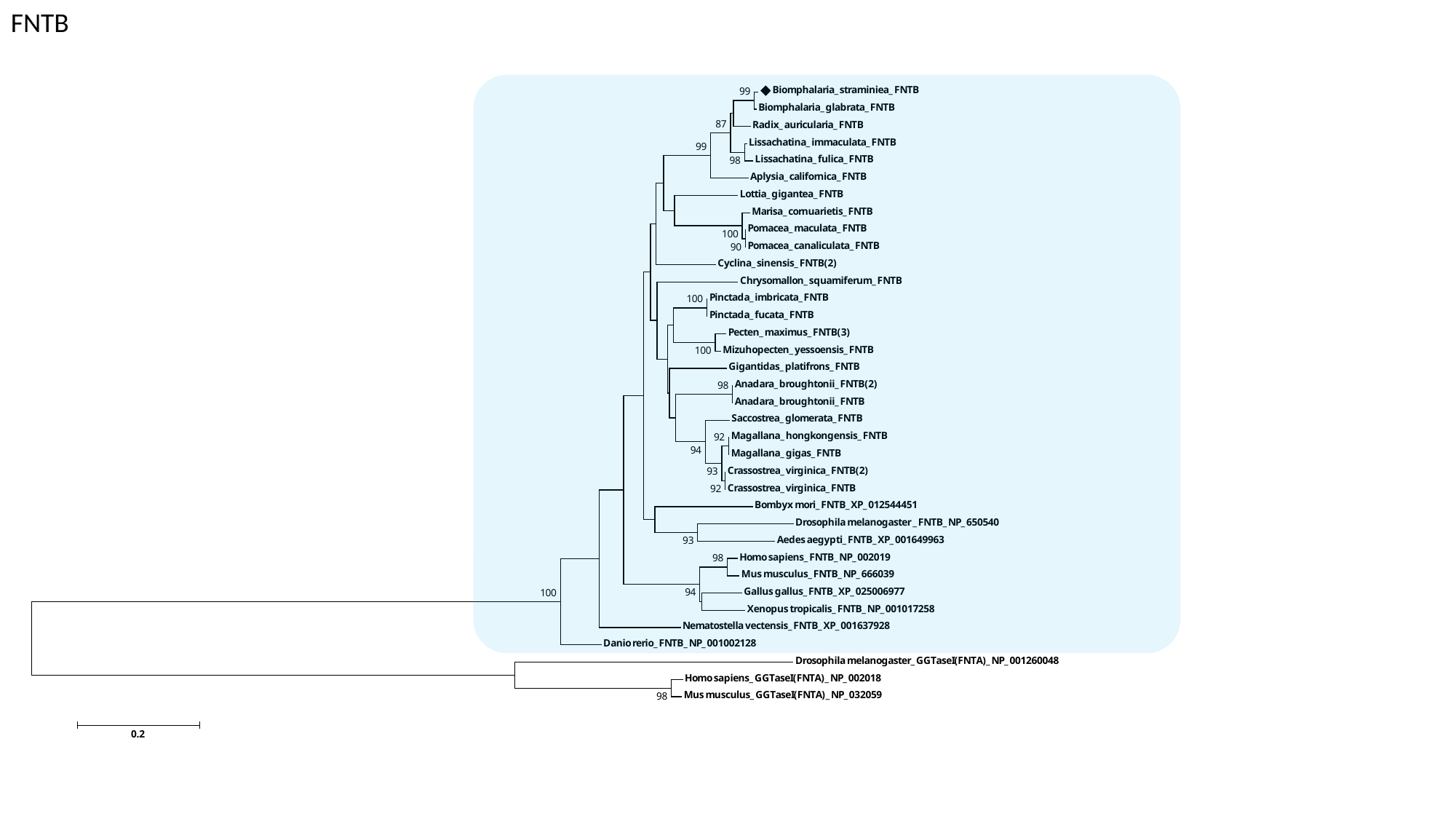

FNTB

## Slide 9
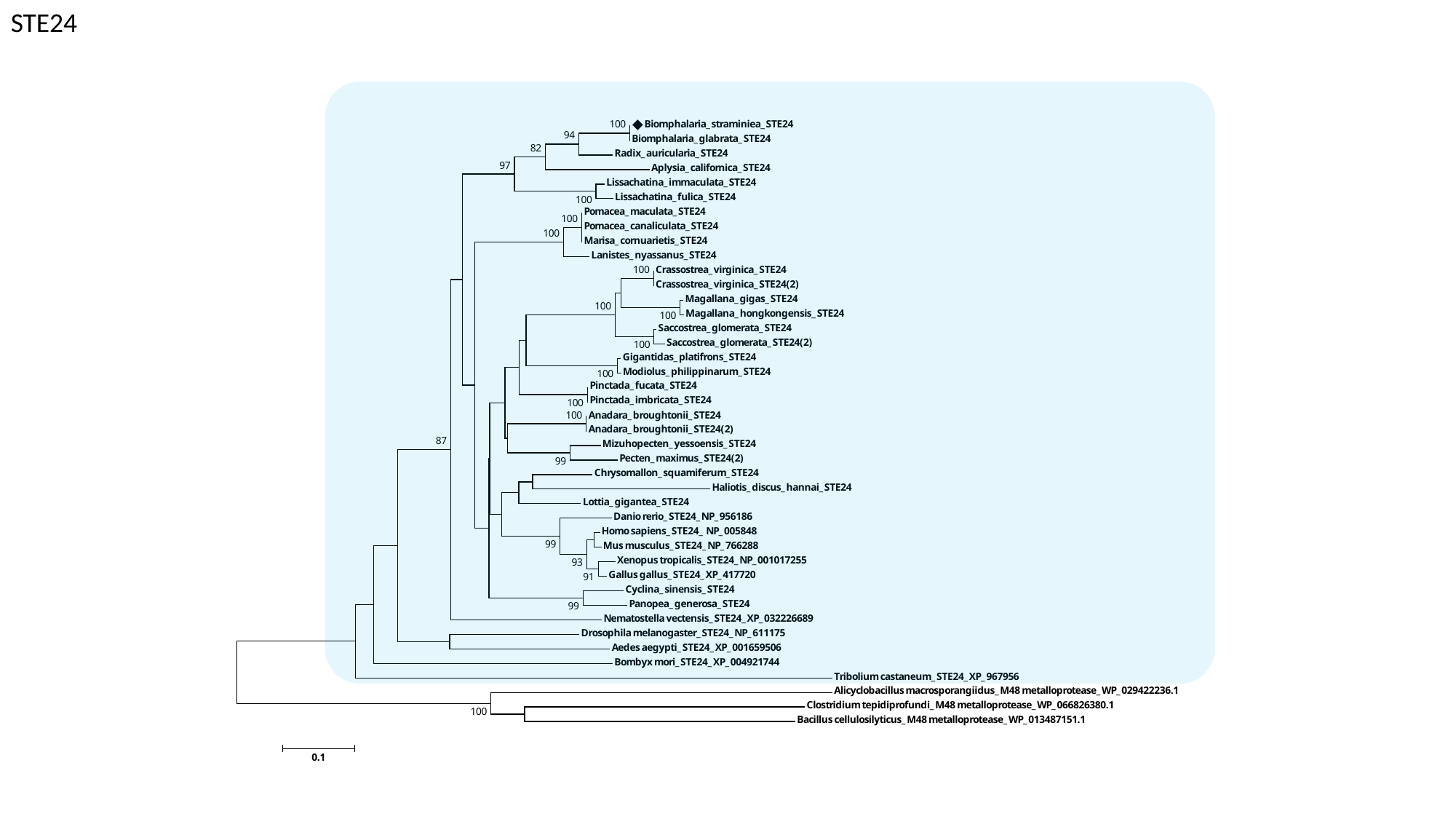

STE24

## Slide 10
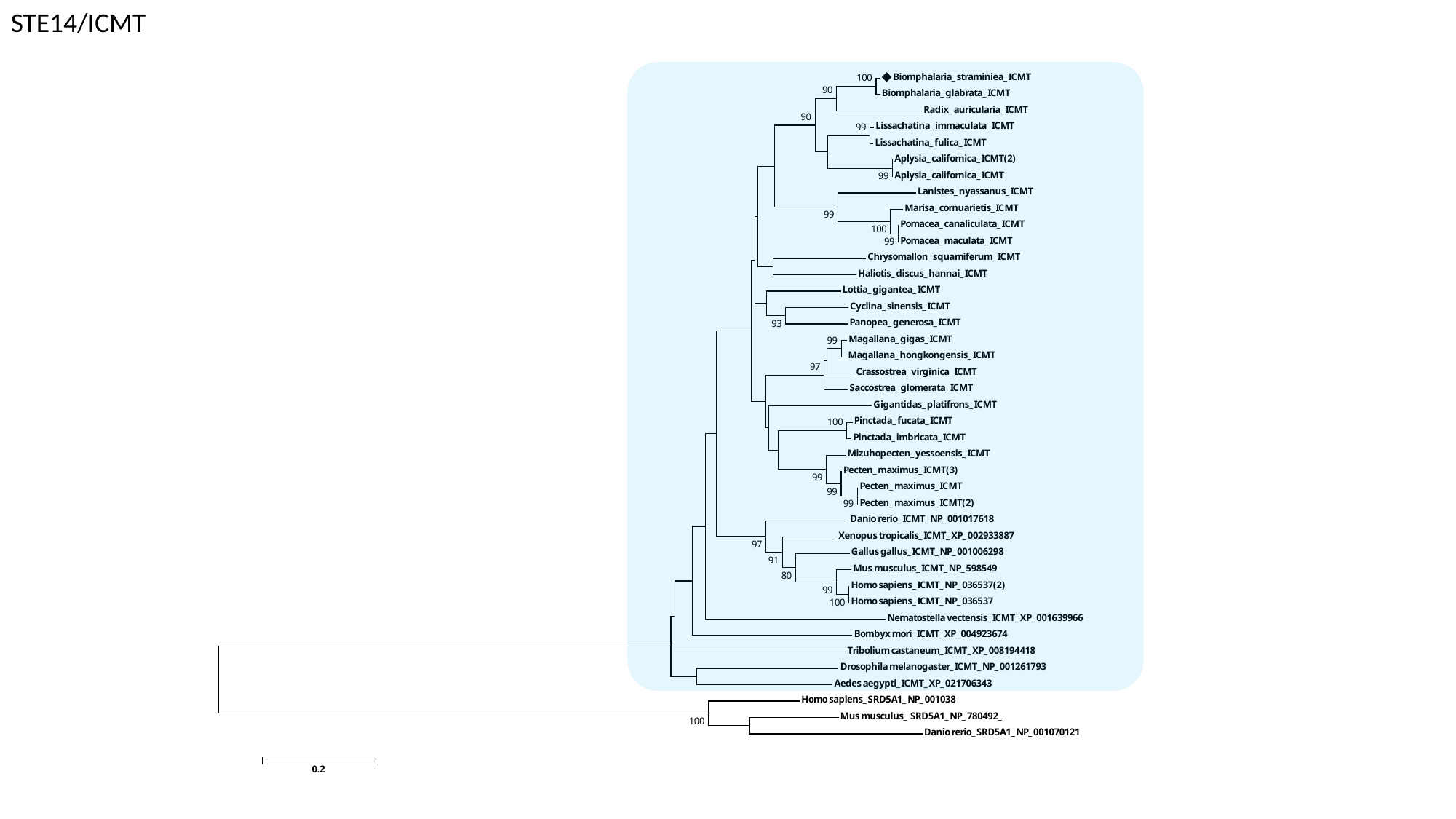

STE14/ICMT

## Slide 11
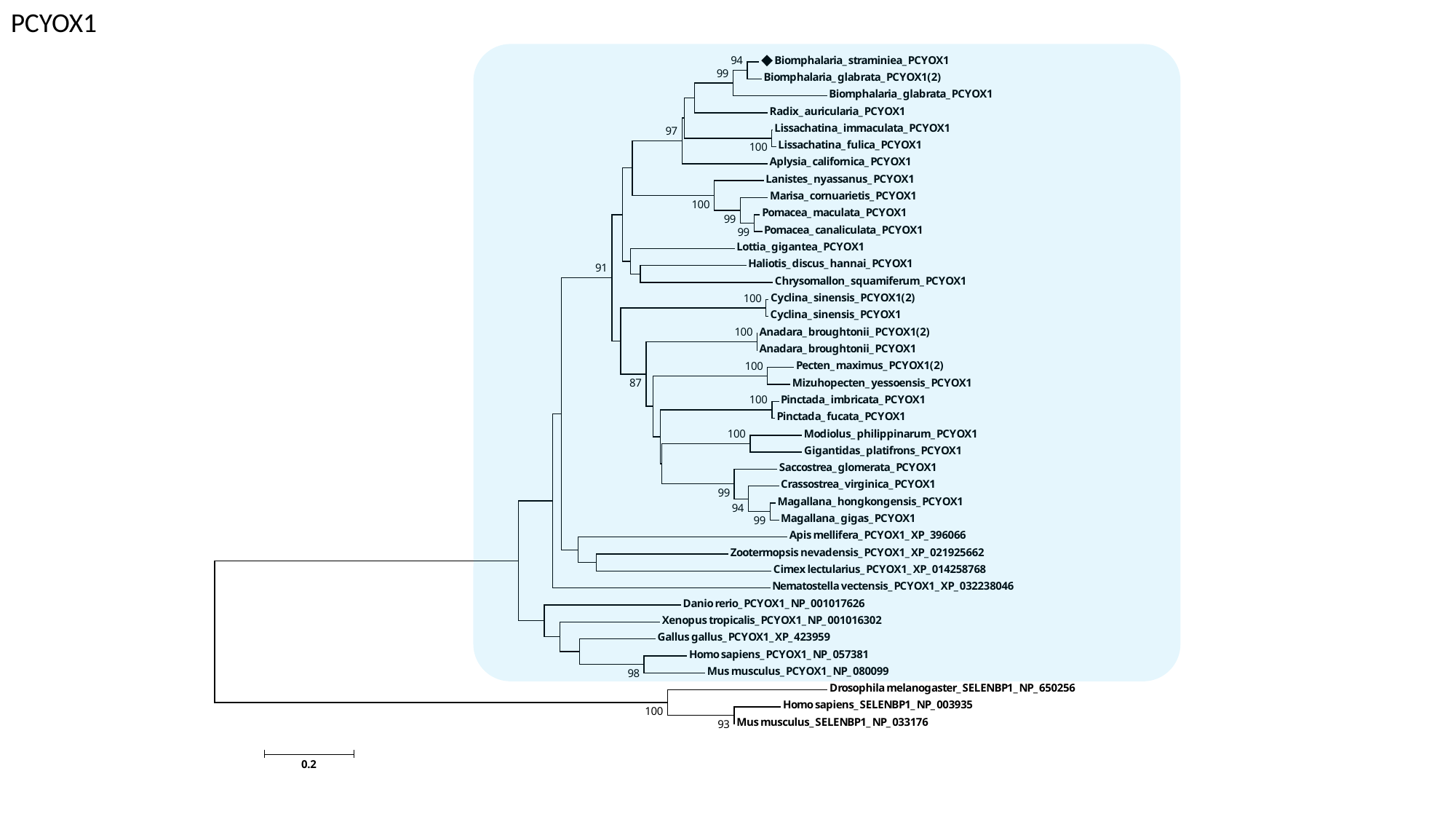

PCYOX1

## Slide 12
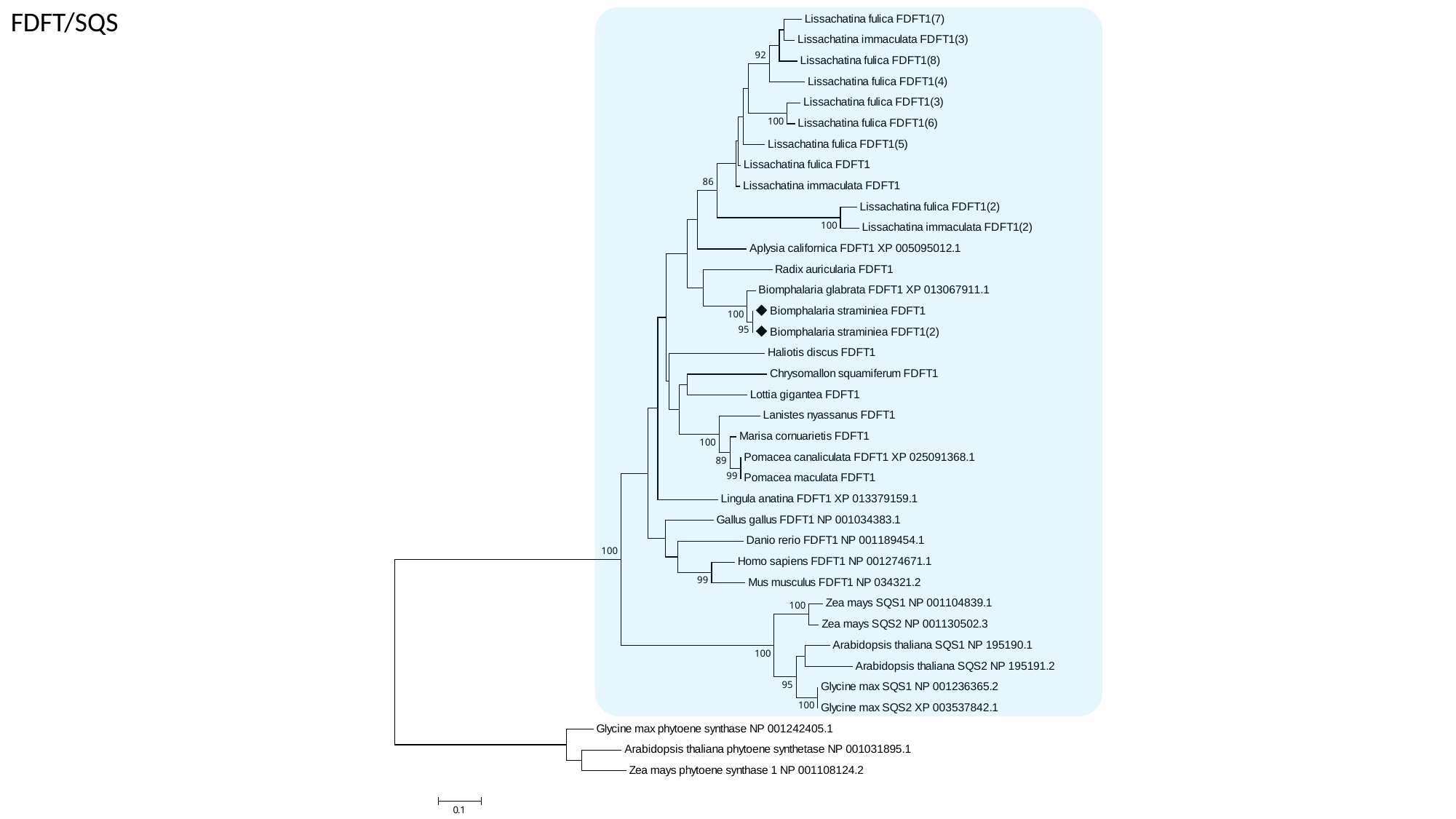

FDFT/SQS

## Slide 13
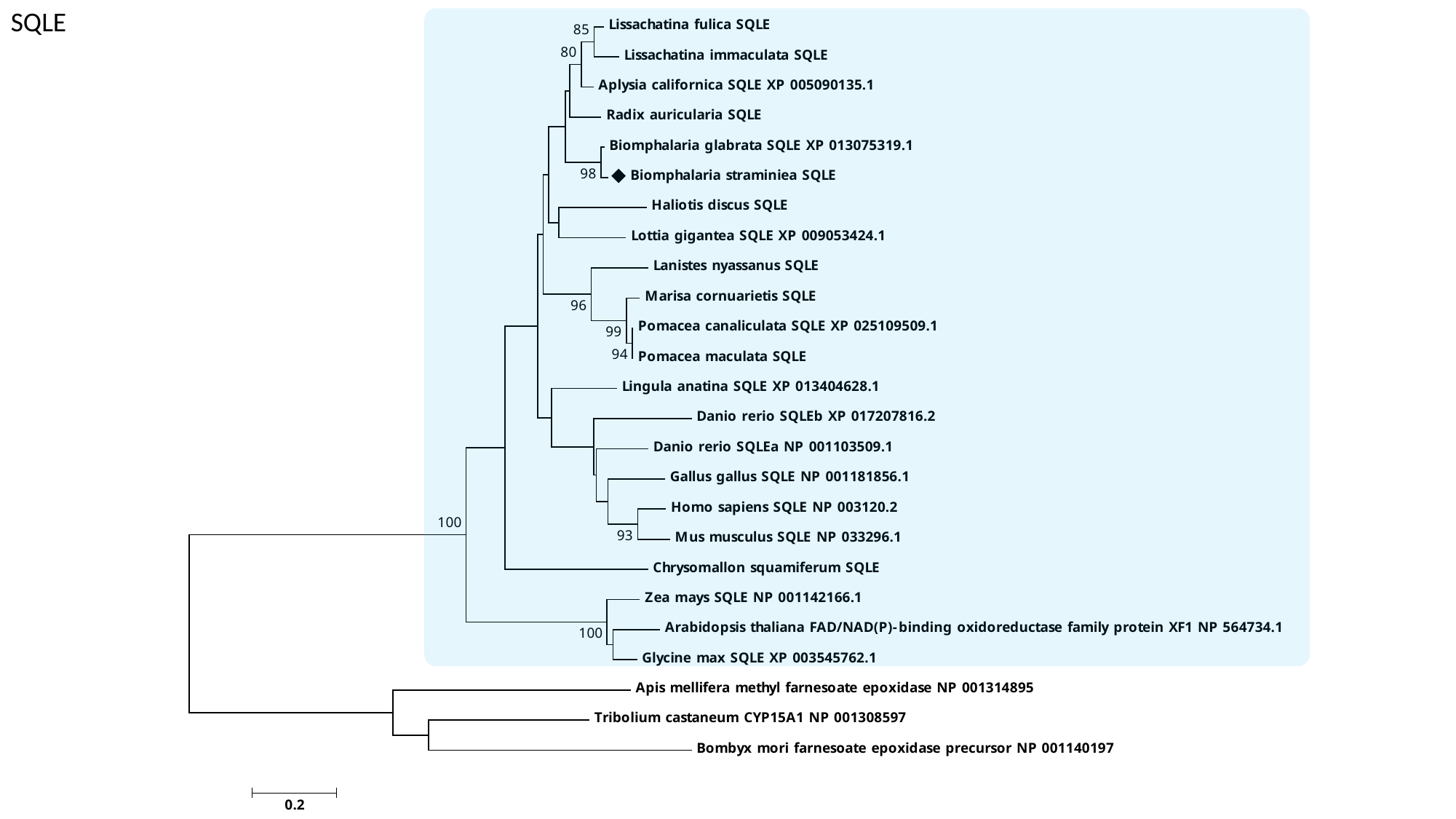

SQLE

## Slide 14
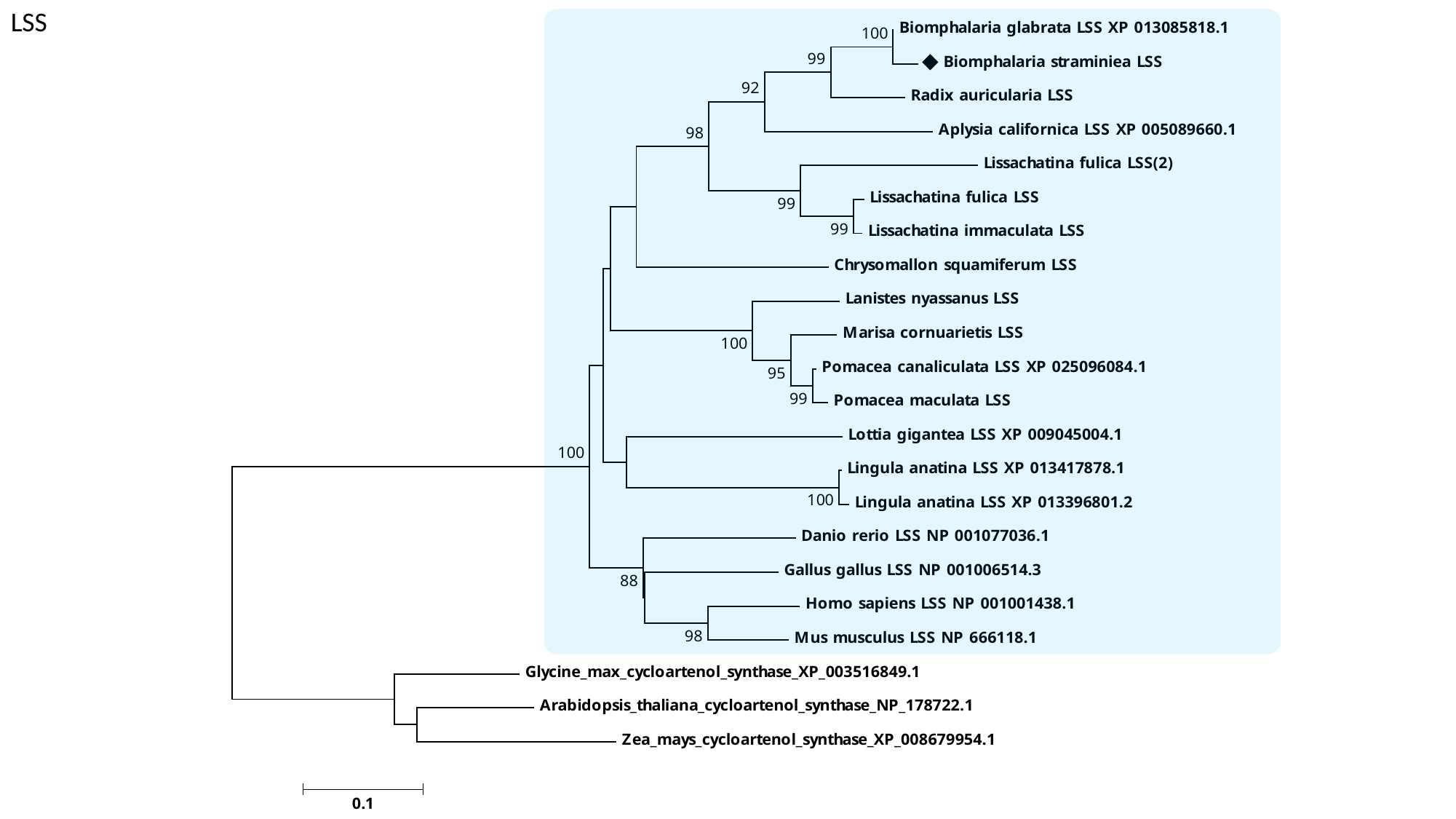

LSS

## Slide 15
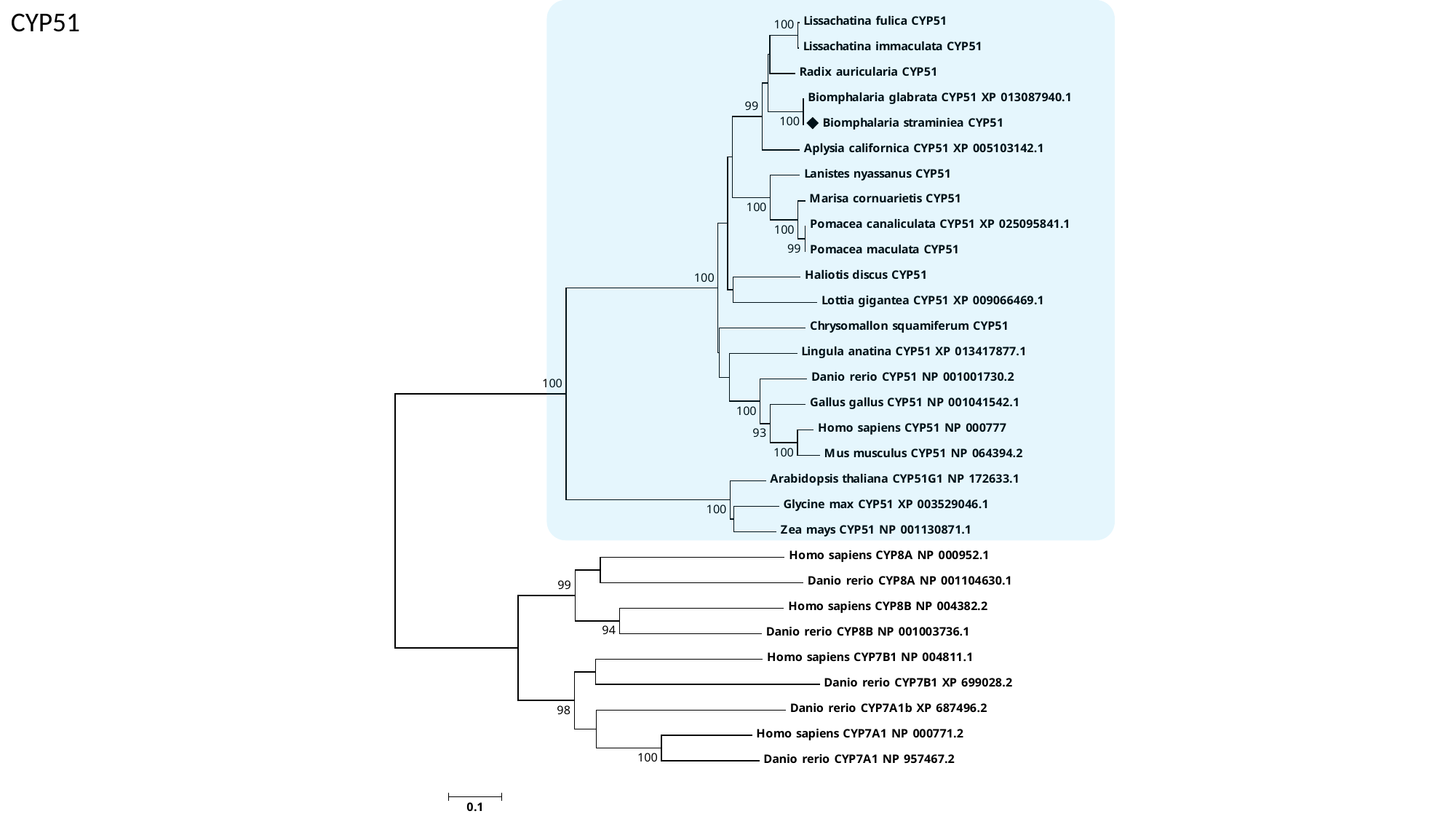

CYP51

## Slide 16
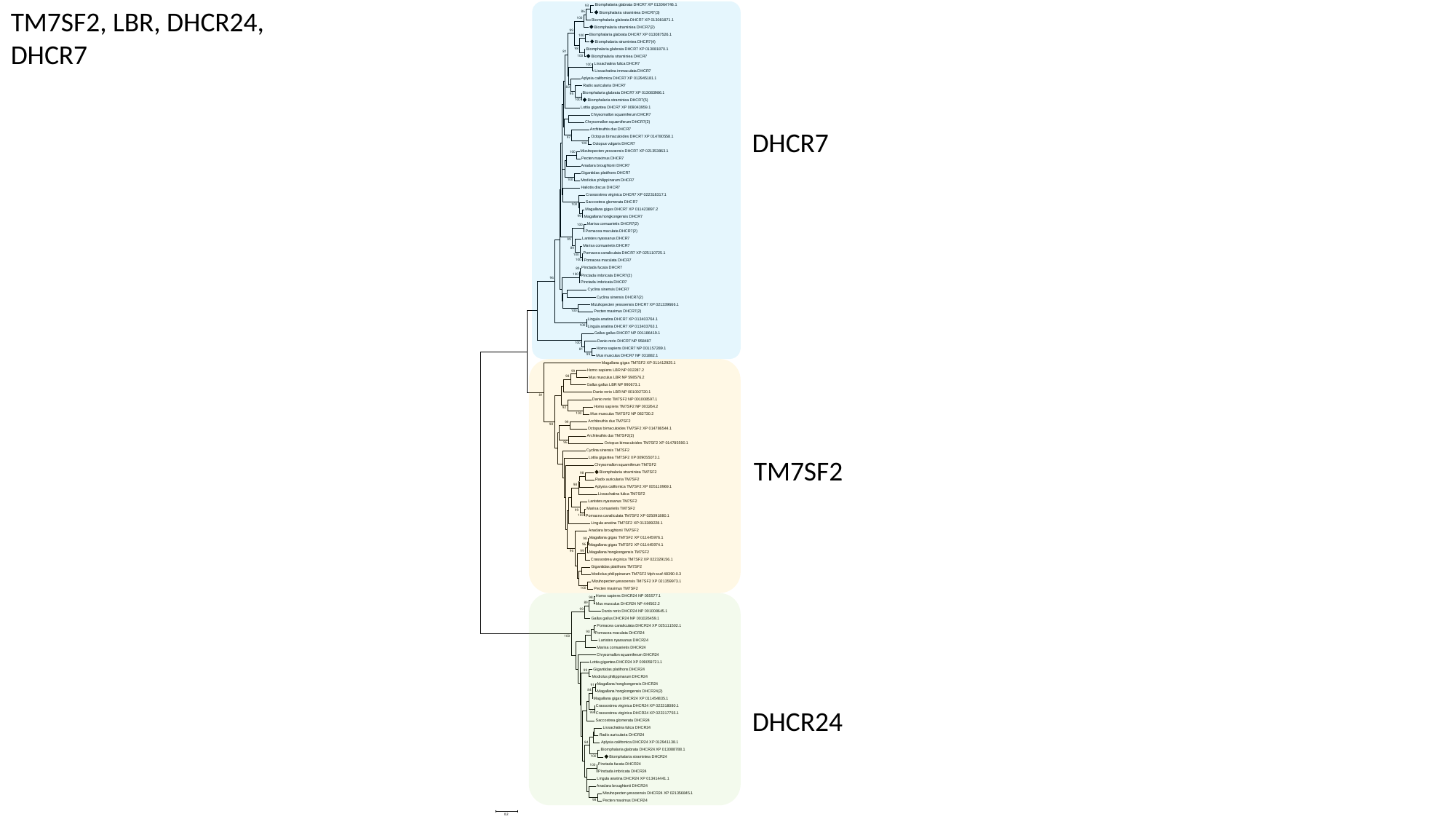

TM7SF2, LBR, DHCR24, DHCR7
DHCR7
TM7SF2
DHCR24

## Slide 17
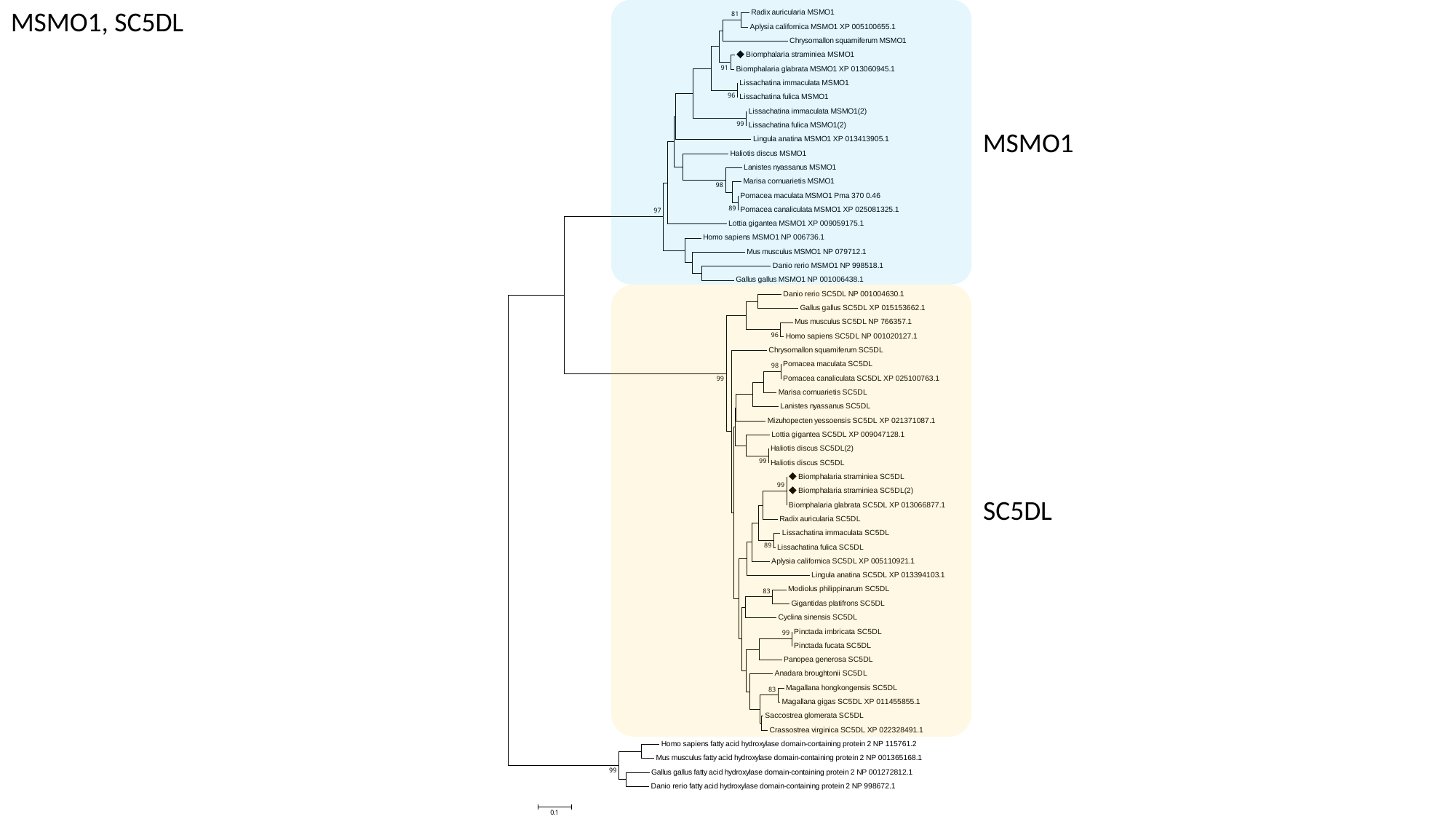

MSMO1, SC5DL
MSMO1
SC5DL

## Slide 18
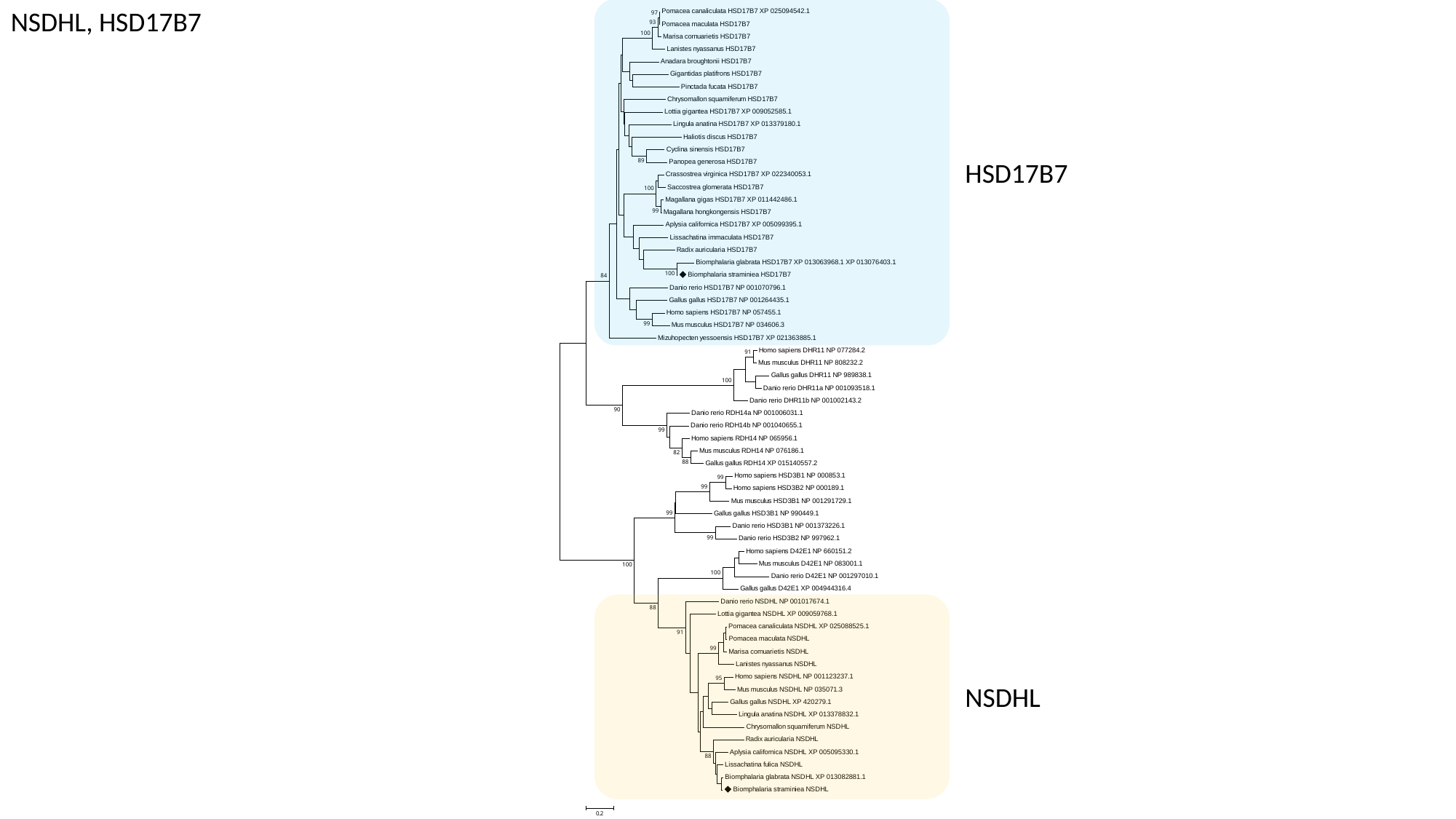

NSDHL, HSD17B7
HSD17B7
NSDHL

## Slide 19
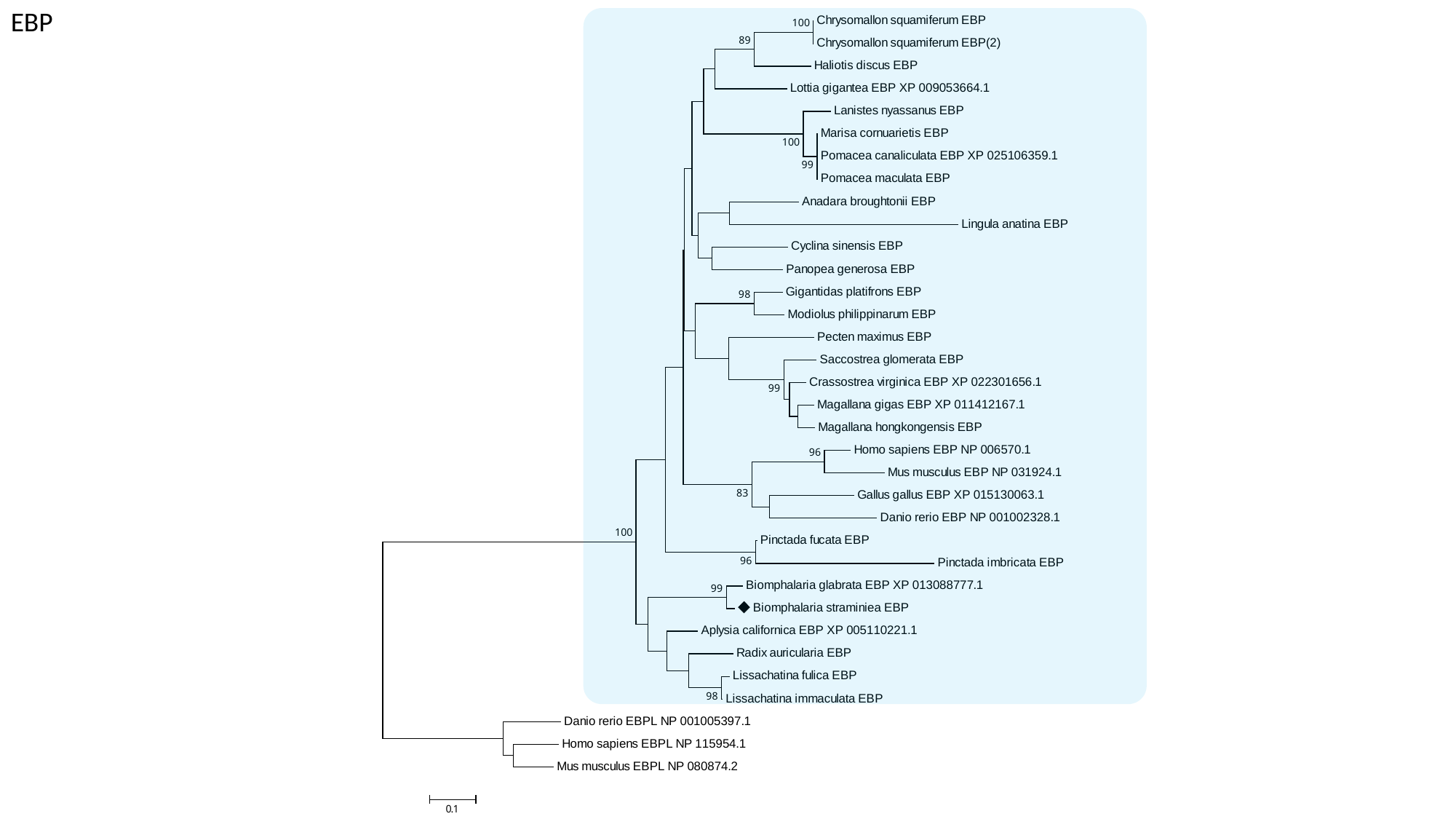

EBP

## Slide 20
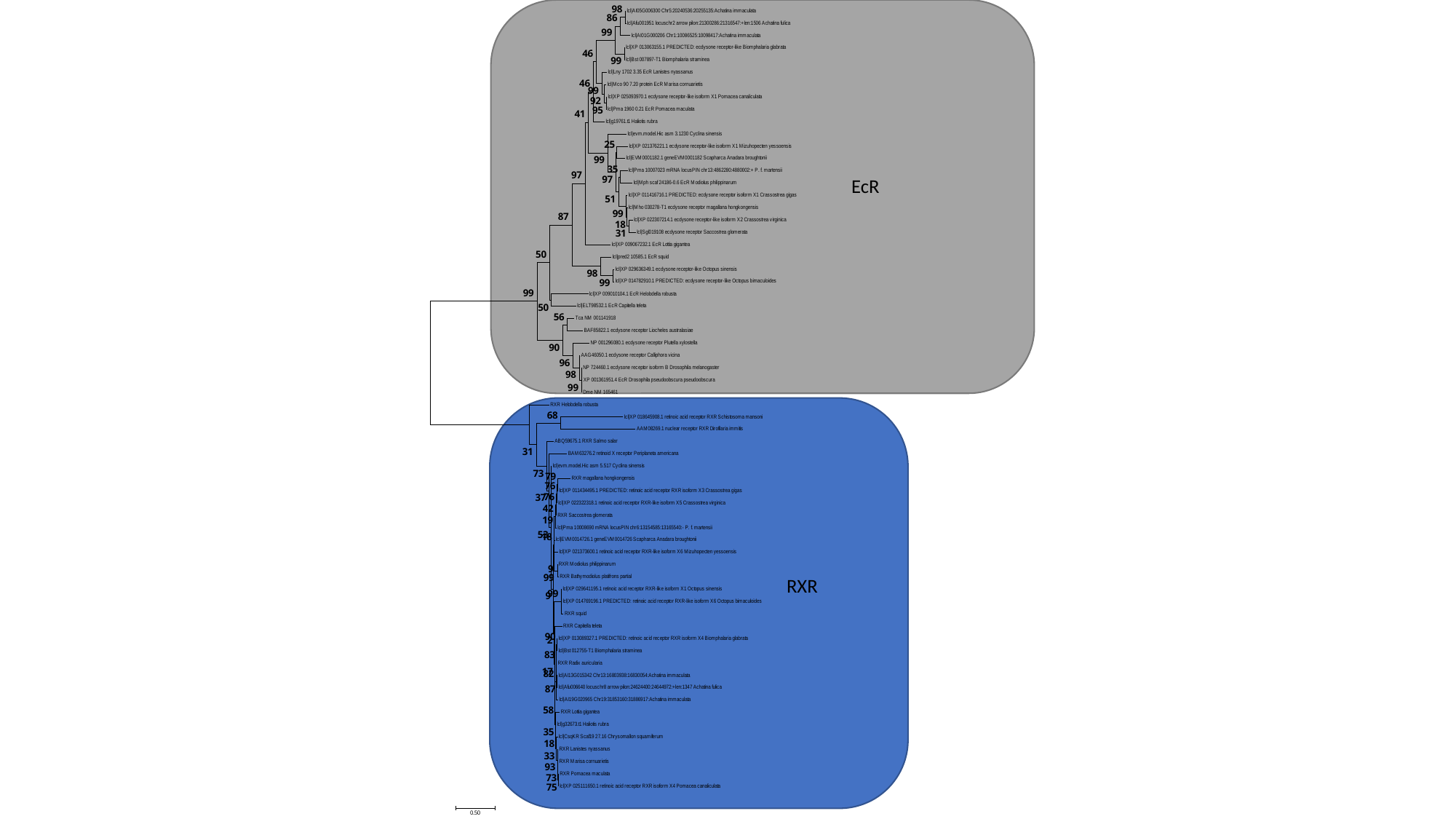

EcR
RXR

## Slide 21
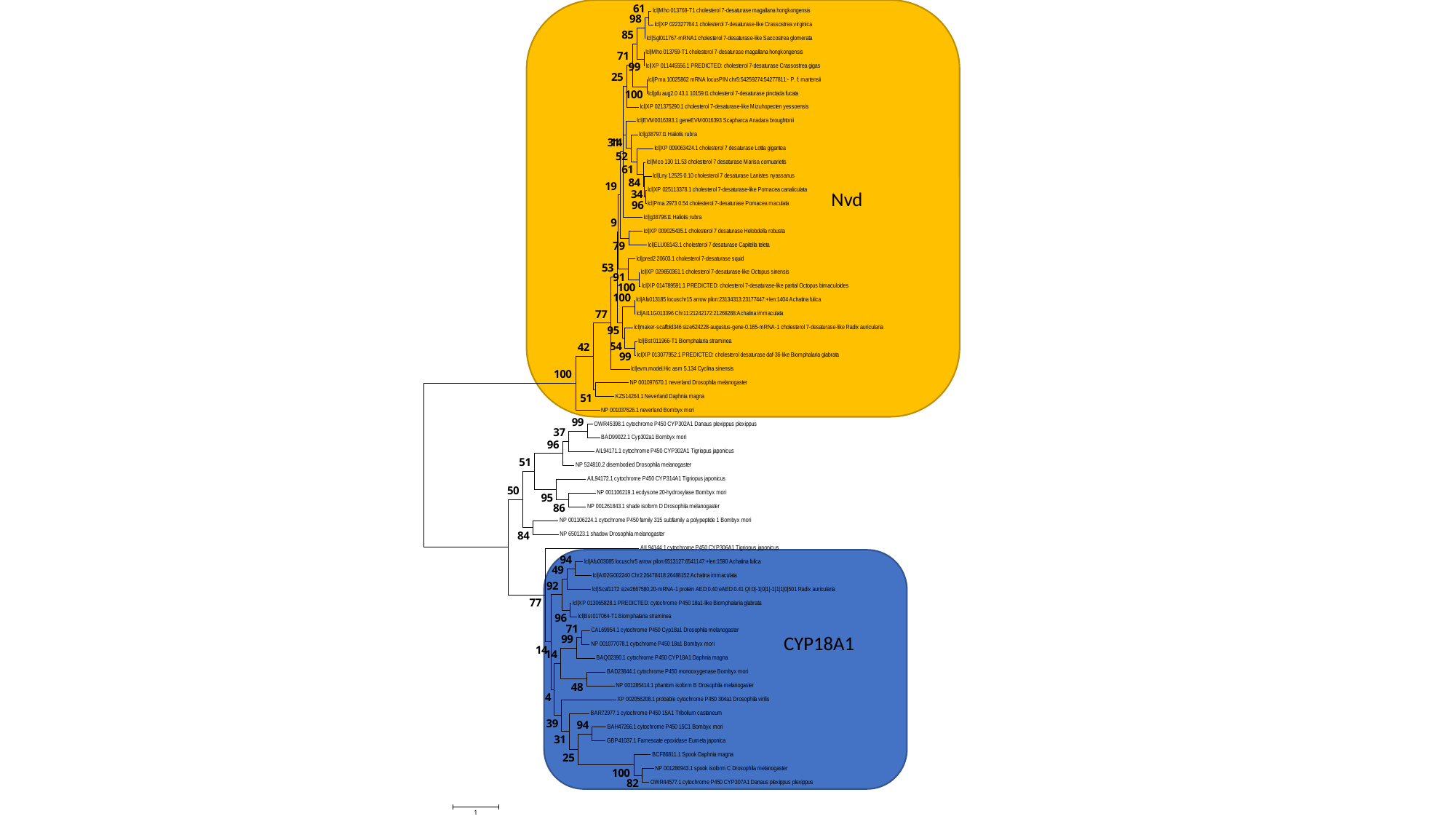

Nvd
CYP18A1
