# Supplementary figures and images for: Genome of the ramshorn snail Biomphalaria straminea—an obligate intermediate host of schistosomiasis
Source: Gigascience. 2022 Feb 15;11:giac012. doi: 10.1093/gigascience/giac012 (PMC8848322; doi:10.1093/gigascience/giac012)

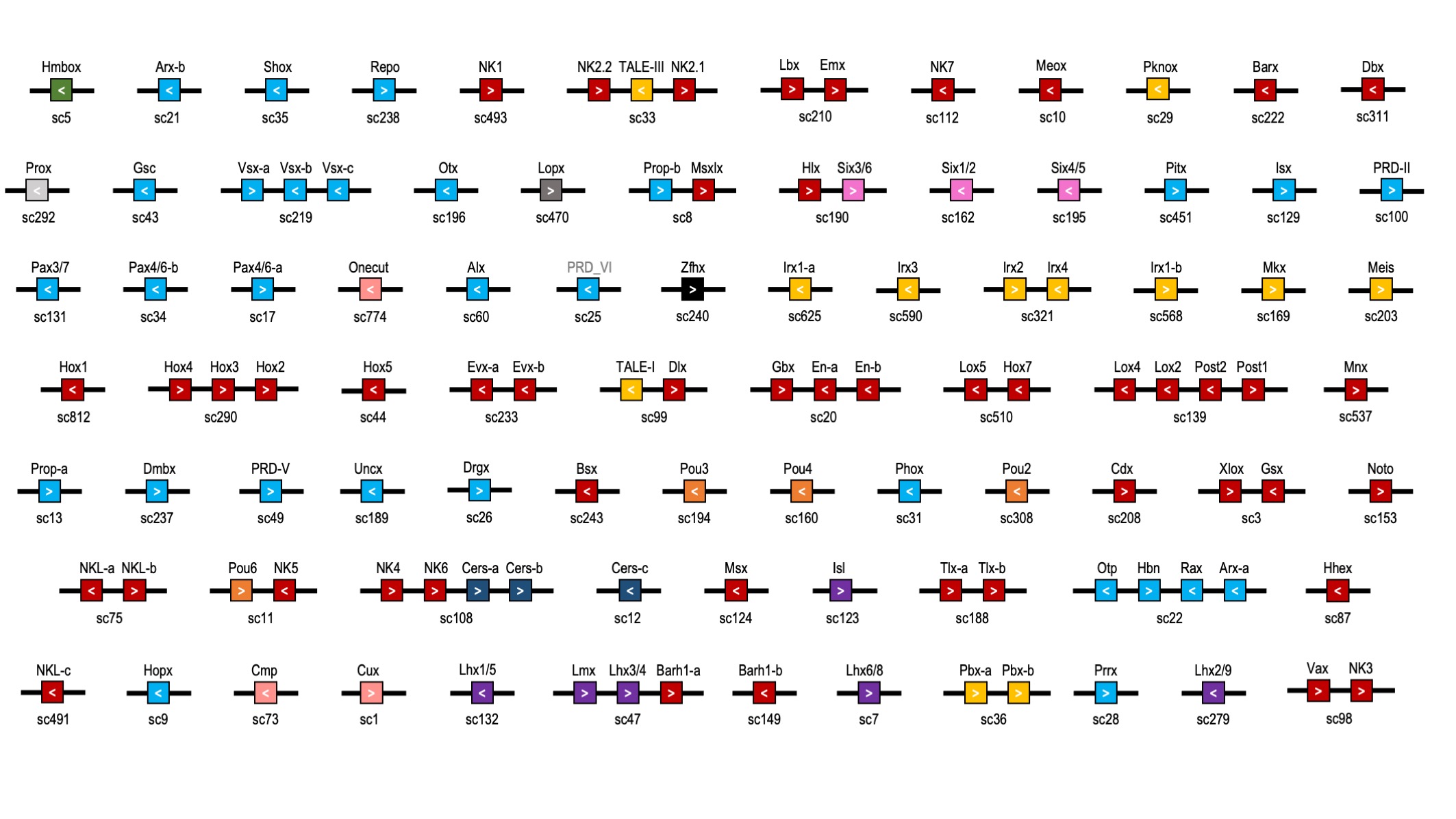

Supplement: giac012_Supplemental_Files [file giac012_supplemental_files.zip › S2b_B.glabrata_Hboxes.jpg]

## Slide 1
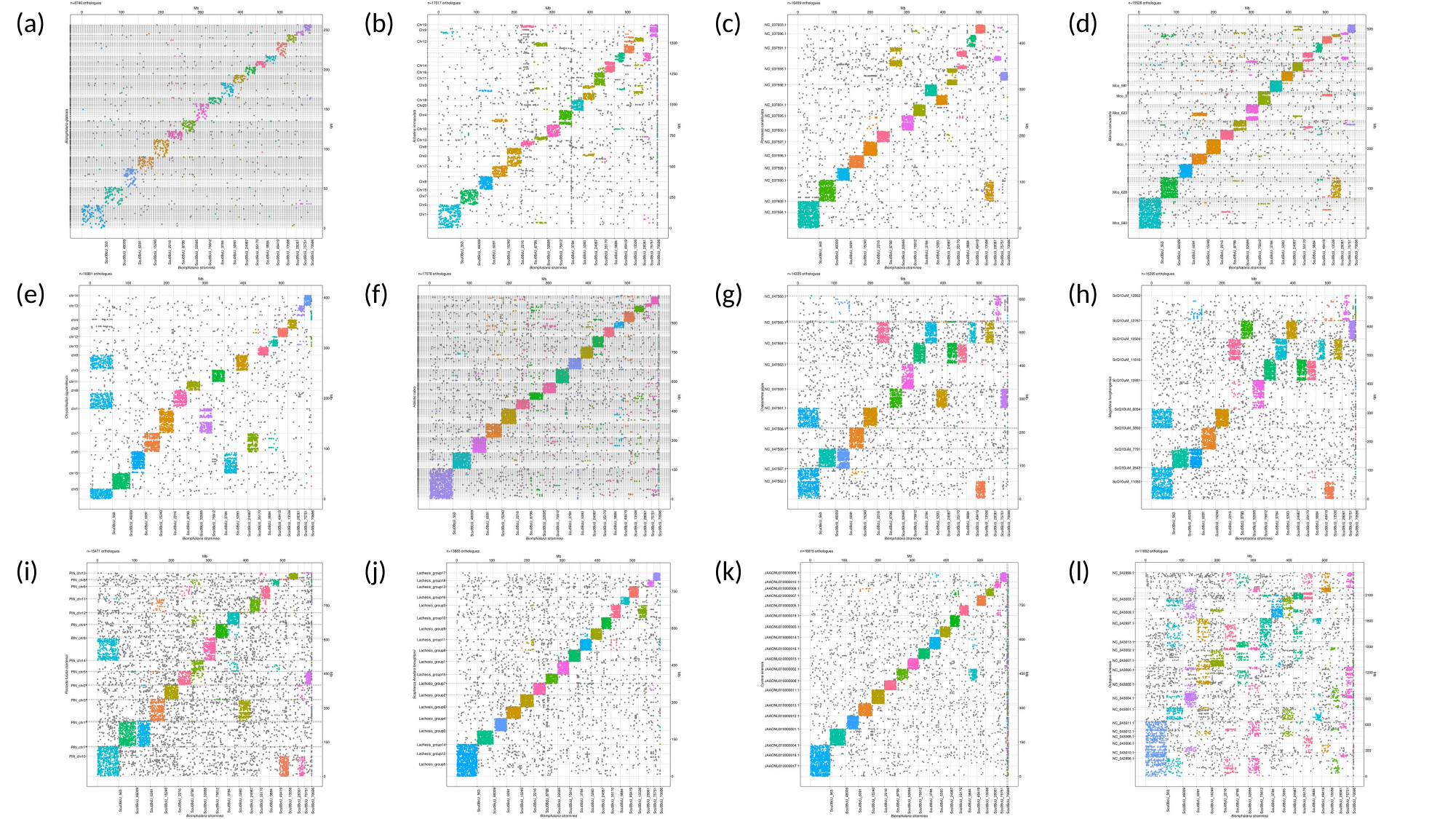

(a)
(b)
(c)
(d)
(e)
(f)
(g)
(h)
(i)
(j)
(k)
(l)

## Slide 2
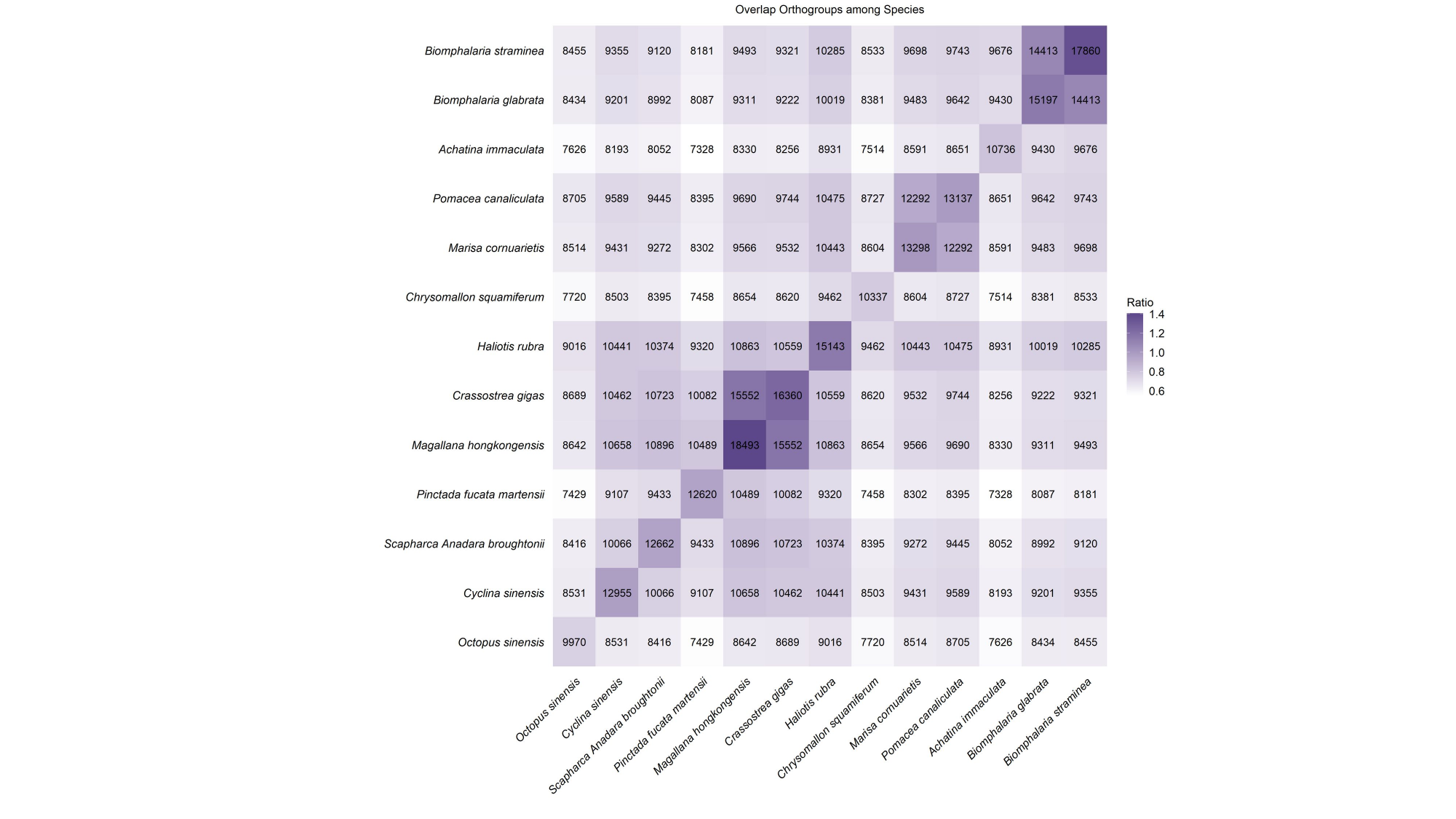

## Slide 3
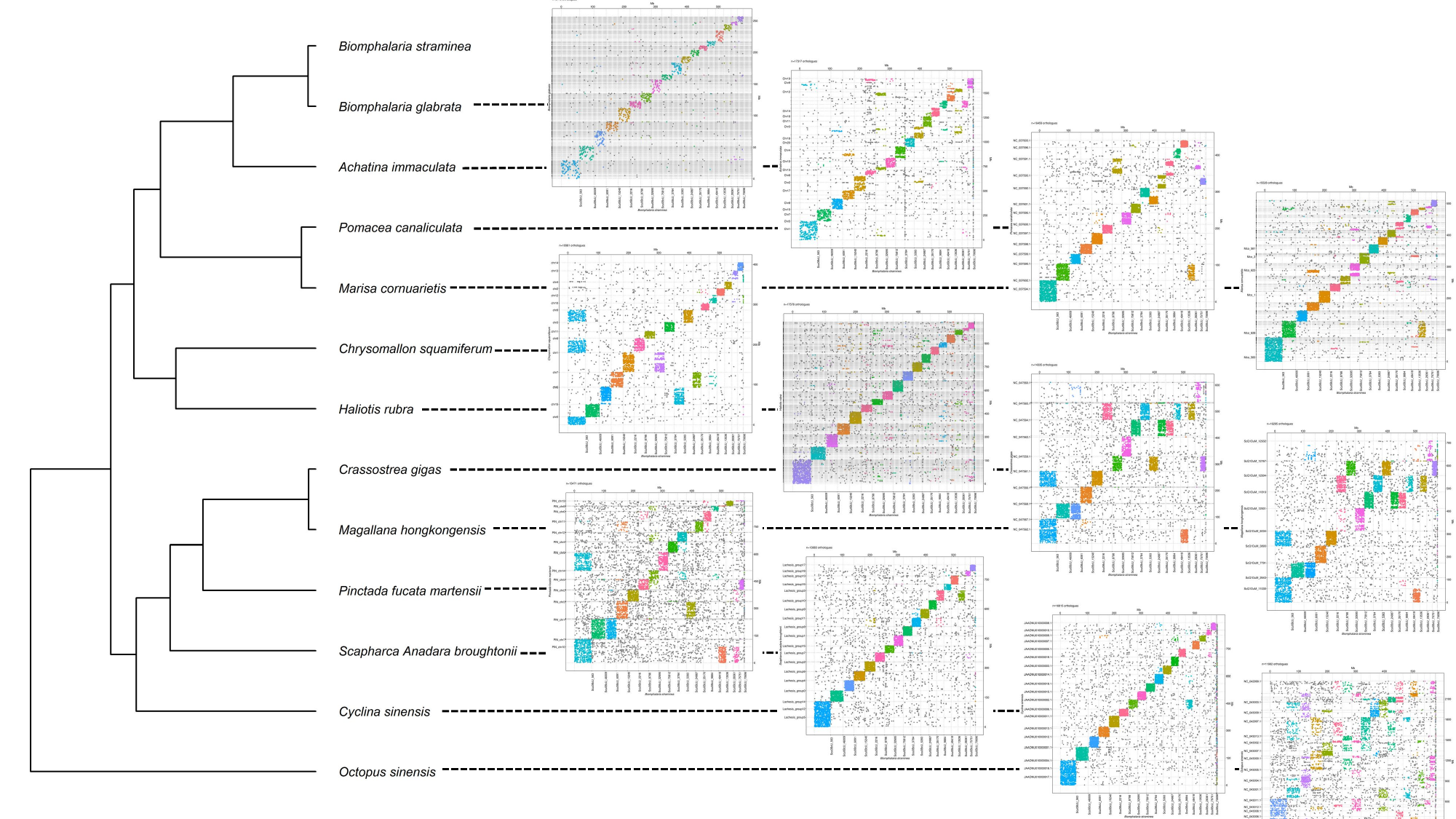

Supplement: giac012_Supplemental_Files [file giac012_supplemental_files.zip › S5. Synteny.pptx]
