# Supplementary material for: Genome of the ramshorn snail Biomphalaria straminea—an obligate intermediate host of schistosomiasis
Source: Gigascience. 2022 Feb 15;11:giac012. doi: 10.1093/gigascience/giac012 (PMC8848322; doi:10.1093/gigascience/giac012)
Supplement: giac012_Response_to_Reviewer_Comments_Revision_1 [file giac012_response_to_reviewer_comments_revision_1.pdf]

Author's Response To Reviewer Comments

Close

The data availability section has now been completed as requested. Thank you.

Close
