# Supplementary material for: Genome of the ramshorn snail Biomphalaria straminea—an obligate intermediate host of schistosomiasis
Source: Gigascience. 2022 Feb 15;11:giac012. doi: 10.1093/gigascience/giac012 (PMC8848322; doi:10.1093/gigascience/giac012)
Supplement: giac012_Reviewer_1_Report_Original_Submission — Jacob Tennessen -- 8/28/2021 Reviewed [file giac012_reviewer_1_report_original_submission.pdf]

Reviewer Report

Title: Genome of the ramshorn snail Biomphalaria straminea - an obligate intermediate host of schistosomiasis

Version: Original Submission **Date:** 8/28/2021

Reviewer name: Jacob Tennessen

Reviewer Comments to Author:

This paper describes a new genome assembly for the snail Biomphalaria straminea. Reported contiguity is much higher even than the existing assemblies for the model snail in the genus, B. glabrata. This assembly will be a useful resource for research on B. straminea and for studies of the genus as a whole, including B. glabrata and the African snail vectors which have the largest impact on human health. The descriptions of gene families and synteny are insightful. Inclusion of experimental data (drug and hormone treatment) is unusual for a genome paper but not inappropriate. However these results are somewhat buried in the paper; there is no indication in the abstract that there is any RNA data, even though the authors sequenced the entire transcriptome and conducted RT-qPCR on candidate genes. The authors may wish to emphasize this aspect of the paper more strongly. In general, there is a lack of genomic resources for mollusks given the importance of the phylum (see Davison & Neiman 2021, Philos Trans R Soc Lond B Biol Sci), and this genome assembly is an important step toward meeting this need.

Minor comments:

Discussion of the African clade is confusing, as the authors don't explain that B. glabrata is neotropical but a phylogenetic sister to the African Biomphalaria, and no actual African snails were analyzed here. The point is simply that B. straminea and B. glabrata represent two major clades in the genus, and thus the conserved synteny between them suggests it is conserved across the genus. It would be good to cite Campbell again in this section or another phylogenetic study (e.g. DeJong et al. 2001 Mol Biol Evol).

The statement "Perhaps more thorough sequencing of B. glabrata could determine if this is shared in the genus" is correct, but it would be even more useful to have genomes from additional Biomphalaria species, not just more B. glabrata.

Methods

Are the methods appropriate to the aims of the study, are they well described, and are necessary controls included? Choose an item.

Conclusions

Are the conclusions adequately supported by the data shown? Choose an item.

Reporting Standards

Does the manuscript adhere to the journal's guidelines on [minimum standards of reporting?]() Choose an item.

Choose an item.

Statistics

Are you able to assess all statistics in the manuscript, including the appropriateness of statistical tests used? Choose an item.

Quality of Written English

Please indicate the quality of language in the manuscript: Choose an item.

Declaration of Competing Interests

Please complete a declaration of competing interests, considering the following questions:

- Have you in the past five years received reimbursements, fees, funding, or salary from an organisation that may in any way gain or lose financially from the publication of this manuscript, either now or in the future?
- Do you hold any stocks or shares in an organisation that may in any way gain or lose financially from the publication of this manuscript, either now or in the future?
- Do you hold or are you currently applying for any patents relating to the content of the manuscript?
- Have you received reimbursements, fees, funding, or salary from an organization that holds or has applied for patents relating to the content of the manuscript?
- Do you have any other financial competing interests?
- Do you have any non-financial competing interests in relation to this paper?

If you can answer no to all of the above, write 'I declare that I have no competing interests' below. If your reply is yes to any, please give details below.

I declare that I have no competing interests

I agree to the open peer review policy of the journal. I understand that my name will be included on my report to the authors and, if the manuscript is accepted for publication, my named report including any attachments I upload will be posted on the website along with the authors' responses. I agree for my report to be made available under an Open Access Creative Commons CC-BY license (http://creativecommons.org/licenses/by/4.0/). I understand that any comments which I do not wish to be included in my named report can be included as confidential comments to the editors, which will not be published.

Choose an item.

To further support our reviewers, we have joined with Publons, where you can gain additional credit to further highlight your hard work (see: https://publons.com/journal/530/gigascience). On publication of this paper, your review will be automatically added to Publons, you can then choose whether or not to claim your Publons credit. I understand this statement.

Yes Choose an item.
