# Supplementary material for: Genome of the ramshorn snail Biomphalaria straminea—an obligate intermediate host of schistosomiasis
Source: Gigascience. 2022 Feb 15;11:giac012. doi: 10.1093/gigascience/giac012 (PMC8848322; doi:10.1093/gigascience/giac012)
Supplement: giac012_Reviewer_2_Report_Original_Submission — Coen Adema -- 9/5/2021 Reviewed [file giac012_reviewer_2_report_original_submission.pdf]

Reviewer Report

Title: Genome of the ramshorn snail Biomphalaria straminea - an obligate intermediate host of schistosomiasis

Version: Original Submission **Date:** 9/5/2021

Reviewer name: Coen Adema

Reviewer Comments to Author:

This characterization of the genome of Biomphalaria straminea, invasive from South America to Hong Kong and main land China, provides a valuable contribtion to interpret biology of freshwater snail species of the genus Biomphalaria that vector the infectious disease human schistosomiasis. Caused by trematode parasites of the genus Schistosoma, the impact of schistosomiasis on global human health is second only to malaria. The data complement the previously reported Biomphalaria glabrata genome and is highly likely to drive extensive comparative genome analyses to inform disease vector biology and biology of molluscs in general.
The study performs logical analyses of the genome involving repetitive content, Hox gene complement and organization, synteny with available molluscan assemblies, gene expansions, presence of genes of metabolic/hormonal pathways. The latter includes an experimental work component.
Although several sequencing approaches are applied, the work did not yield a chromosome level assembly. The authors should discuss why not, and identify likely challenges to chromosome-level assembly for this snail species (repetitive content/genome size?). The reviewer does not perceive this as weakness, rather an indication of complexity of gastropod (molluscan?) genomes.
Also, comparison with other molluscan genomes is valuable but the presentation of the results is less effective because it does not clarify the taxonomic relations of the molluscs included in the study. It is recommended to provide a figure to clarify the taxonomy of Cephalopoda, Bivalvia and Gastropoda in this study. Within Gastropoda, also identify prosobranchs, heterobranchs, eupulmonates and hygrophila.
Consistent use of deuterostomes, exdysozoan plus lophotrochozoan protostomes and prebilateria would improve clarity.
Finally, the description of the relevant parasitology is incorrect in places, see specific comments for corrections.
Overall, the methods appropriate to the aims of the study, additional details are needed to describe the experimental work with live snails.
The conclusions are adequately supported by the data, although the use of a single stimulus for gene expression of ecdysteroid pathway genes does not seem to equate systemic analysis, also considering the lack of DE.
Quality of language is generally acceptable, specific comments follow below for editing of language in the manuscript.
No specific methods section is provided but this reviewer is not concerned.
specific comments

P1 Title, intermediate vector is incorrect, change to (obligate) intermediate host or vector
P2 Abstract
L6 B. stramina IS native to South America
L7 Reservoir is a parasitological term used for alternative host species, other than the main hosts for a parasite. It is not appropriate for the snail host. Suggest replace "reservoir" by "vector host"
L9 suggest edit hinders to limits
L15-16, indicate range of "other molluscs", does it include the classes gastropoda, bivalvia and cephalopoda?
L18, again specify certain mollusc lineages
L21 suggest edit to biology related to snail vectors of schistosomiasis
P3
L2 240 million are actually infected, these would require treatment, not preventive treatment
L9-10 these eggs do NOT circulate to the liver and leave the body. Eggs are deposited in blood venules and cross the intestinal wall to leave the body with faeces. Eggs that fail to do so are called reflux eggs and these circulate to the liver where they cause pathology. Please correct.
L10 delete contaminated
L12 delete further
L14-15 edit infected by to potential vectors for
L17-18, For native range also cite "DeJong RJ, et al. Evolutionary relationships and biogeography of Biomphalaria (Gastropoda: Planorbidae) with implications regarding its role as host of the human bloodfluke, Schistosoma mansoni. Mol Biol Evol. 2001 Dec;18(12):2225-39. doi: 10.1093/oxfordjournals.molbev.a003769. PMID: 11719572."
P4
L4--- Indicate whether parasite was introduced by infected snails, humans or other reservoir hosts.
P5
L3 Unclear with methods(P19,L13-18) indicate how many snails were used in total, only 1 or more because of using 1 adult snail for each of the sequencing methods. Also indicate source of snails; field isolate (date, latitude longitude of collection), wild type or lab isolate (inbred, outbred, kept for how many generations?).
L5 is the compete mitogenome included as one of the scaffolds
P6
L3, here and in figure, explain "spike in activity", is this a large number of sequences with only few lethal mutations, how is this shown in figure 1C?
P7
L1 "genes coopted to novel structures", explain, are these genes coding for proteins with novel structures or proteins with novel functions?
L5 "another oyster" provide Genus and species
L6, indicate that Mollusca belong to lophotrochozoa, a main lineage of protostomes along with ecdysozoa and indicate that the vertebrates belong to deuterostomia.
L10, and elsewhere (e.g. P9, L3-6) , define clustered and linked, what is the difference?
L12 identify L. gigantea as gastropod
L13 indicate level of ancestor (gastropod, molluscan or lophotrochozoan…)

P10
L19 indicate classes for limpet and oyster (Gastropoda and Bivalvia, respectivelyP11
L14 define widely found, perhaps as broadly distributed?
P14
L13 suggest include "or assemblies of additional Biomphalaria species"
L14 what are potential impacts?
L16 "good markers", to what extent?
L18-21. This interesting comparative genome analyses may deserve a figure?
L22-23 define "mollusc genomes", perhaps "as available from GenBank for gastropod, bivalve and cephalopod molluscs.
P15
L1-2 does the number of pseudochromosomes resemble the number of chromosomes shown by karyotyping B. glabrata (see e.g Adema et al., 2017)
L2 edit genus to genera, and perhaps "of other classes" (see previous comment)
L3-4. cite studies by Campbell and DeJong, mentioned above.
L8 identify Achatina as eupulmonata gastropod, also this species of stylommatophora (landsnail) was proposed to have undergone whole genome duplication, does this complicate the synteny comparison?
L11 indicate that this would be an ancestor of the Hygrophila and Eupulmonata
L12-14, for accuracy, text actually lists molluscs with greater phylogenetic distance (prosobranch gastropods and Bivalvia), revise accordingly.
L16-17 provide citation for divergence and ancestor?
P16
L7-10. Suggest to remove fig 3C, the text adequately conveys the lack of altered expression. Indicate what other organisms do respond to the stimulus employed, suggesting differences in biology?
L19 define high memory scores
P17
L7, Is this pattern similar or both Biomphalaria species?
L11 suggest edit "in certain" to "among"
L16 "its adaptation" to what?
L17 (also P18, L3-4) Are these HSPs (types and numbers) in accordance with the B. glabrata genome, Adema et al 2017?
P18
L15 suggest edit "can change" to "changed"
P19,
L3 suggest edit "holding" to "having"
L4 "dynamics" is not clear, revise to other word?
L6 edit to ….. glabrata as well as among other molluscan taxa. Or similar
P23
L1-2 How were the initial sequences used for the searches selected?
L14-17, add idnetifiers for the species used as deuterostomes, and ecdysozoan or lophotrochozoan protostomes, also indicate oyster is bivalvia, limpet is gastropod.
P25

L9 define adult by shell diameter size, or confirmed by reproductive maturity. How was culture initiated? was this from a multiple individual collected from the field or from a single snail by selfing? For how long was the culture maintained at time of experiments?
P26
provide primers used for the gene targets. Was target specificity tested? Myoglobin may have sequence similarity with the multi domain sequences of Biomphalaria hemoglobin.
Figure 4
identify in figure branches of heterobranchs, prosobranchs,gastropods, bivalves and cephalopods. (similar to fig 5b)
Again can the number of (pseudo)chromosome clusters be equated with chromosome numbers recorded for Biomphalaria sp?
Fig 7B indicate branches for lophotrochozoan protostomes, ecdysozoan protostomes and deuterostomes

Methods

Are the methods appropriate to the aims of the study, are they well described, and are necessary controls included? Choose an item.

Conclusions

Are the conclusions adequately supported by the data shown? Choose an item.

Reporting Standards

Does the manuscript adhere to the journal's guidelines on [minimum standards of reporting?]() Choose an item.

Choose an item.

Statistics

Are you able to assess all statistics in the manuscript, including the appropriateness of statistical tests used? Choose an item.

Quality of Written English

Please indicate the quality of language in the manuscript: Choose an item.

Declaration of Competing Interests

Please complete a declaration of competing interests, considering the following questions:

- Have you in the past five years received reimbursements, fees, funding, or salary from an organisation that may in any way gain or lose financially from the publication of this manuscript, either now or in the future?
- Do you hold any stocks or shares in an organisation that may in any way gain or lose financially from the publication of this manuscript, either now or in the future?
- Do you hold or are you currently applying for any patents relating to the content of the manuscript?
- Have you received reimbursements, fees, funding, or salary from an organization that holds or has applied for patents relating to the content of the manuscript?
- Do you have any other financial competing interests?
- Do you have any non-financial competing interests in relation to this paper?

If you can answer no to all of the above, write 'I declare that I have no competing interests' below. If your reply is yes to any, please give details below.

I declare that I have no competing interests

I agree to the open peer review policy of the journal. I understand that my name will be included on my report to the authors and, if the manuscript is accepted for publication, my named report including any attachments I upload will be posted on the website along with the authors' responses. I agree for my report to be made available under an Open Access Creative Commons CC-BY license (http://creativecommons.org/licenses/by/4.0/). I understand that any comments which I do not wish to be included in my named report can be included as confidential comments to the editors, which will not be published.

Choose an item.

To further support our reviewers, we have joined with Publons, where you can gain additional credit to further highlight your hard work (see: https://publons.com/journal/530/gigascience). On publication of this paper, your review will be automatically added to Publons, you can then choose whether or not to claim your Publons credit. I understand this statement.

Yes Choose an item.
